# Enhanced visible-light photocatalysis by Au and Ag decorated ZnO for the simultaneous degradation of tetracycline and methylene blue

**DOI:** 10.1039/d5na00878f

**Published:** 2026-01-06

**Authors:** Asha Kumawat, Sunil Chhichholiya, Mamta Devi Sharma, Poonam Kumari, Rajesh Kumar Meena, Pragati Fageria

**Affiliations:** a Centre of Advanced Studies, Department of Chemistry, University of Rajasthan Jaipur 332004 India pragati.fageria@gmail.com; b Department of Clean Energy and Fuel Cell, Dr Bansi Dhar Institute Gurugram Haryana 122003 India; c Department of Chemistry, Kalindi College, University of Delhi India

## Abstract

Pristine and noble metal-decorated ZnO nanostructures were synthesized *via* a simple chemical reduction approach using hydrazine hydrate to deposit silver (Ag) and gold (Au) nanoparticles. Comprehensive characterization using PXRD, FESEM, HRTEM, XPS, FTIR, Raman, BET, and UV-vis spectroscopy revealed high-surface-area nanostructures with enhanced optical properties. Photocatalytic evaluation demonstrated that Au- and Ag-decorated ZnO exhibited significantly improved degradation efficiencies compared to bare ZnO under visible-light irradiation, attributed to improved charge carrier separation and extended visible-light absorption *via* plasmonic resonance effects. Notably, the catalysts showed excellent reusability over multiple cycles. Most significantly, the as-synthesized nanocomposite exhibited remarkable capability for the simultaneous co-degradation of two structurally and chemically distinct pollutants: tetracycline (TC, pharmaceutical antibiotic) and methylene blue (MB, textile dye). Under identical visible-light conditions at pH 7, Au-ZnO achieved 95% degradation of TC (2.0 × 10^−3^ M) and 80% degradation of MB (1.0 × 10^−5^ M) within 120 min using only 20 mg catalyst. This simultaneous removal of pharmaceuticals and dyes in a single photocatalytic process demonstrates the potential of dual-pollutant degradation. The single-platform capability for degrading structurally diverse pollutants suggests that noble metal-modified ZnO warrants further investigation as a multifunctional photocatalyst for treating complex wastewater containing mixed organic contaminants.

## Abstract 2

Au and Ag-decorated ZnO NSs were synthesized and demonstrated as the first dual-target photocatalyst capable of simultaneously degrading pharmaceutical contaminants (TC) and textile dyes (MB) under visible light.

## Graphical abstract

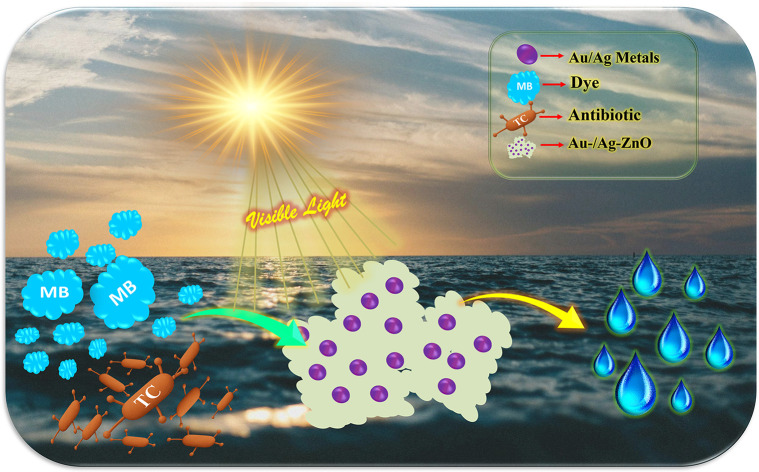

## 1. Introduction

The rapid pace of industrialization, urban expansion, and pharmaceutical consumption has led to the widespread contamination of aquatic environments by persistent organic pollutants (POPs), posing significant risks to environmental sustainability and public health.^1–3^ Among these, pharmaceutical residues such as tetracycline (TC) and synthetic dyes like methylene blue (MB) are particularly alarming due to their chemical stability, low biodegradability, and resistance to conventional water treatment technologies.^4,5^

Tetracycline, a broad-spectrum antibiotic frequently utilized in veterinary and human medicine, is frequently detected in wastewater effluents from domestic, hospital, and agricultural sources.^4,6^ Because of its incomplete metabolism, substantial quantities of TC are released into the environment in a biologically active form. Its persistence in aquatic ecosystems not only exerts toxic effects on non-target organisms but also accelerates the rise of genes and bacteria resistant to antibiotics (ARBs), which can propagate through food chains and water systems, posing a severe global health crisis.^7,8^ Even at low concentrations, TC has been shown to induce cytotoxicity, genotoxicity, and endocrine disruption in aquatic life. Similarly, methylene blue is a versatile cationic dye with widespread applications in the textile and paper industries, as well as significant uses in the pharmaceutical field. Although MB has found historical utility in medical applications, its inherent physicochemical properties present significant environmental challenges. MB exhibits exceptional water solubility, photochemical stability, and pronounced recalcitrance toward microbial biodegradation.^5,9^ The discharge of MB into aquatic ecosystems triggers multiple detrimental effects, including aesthetic deterioration, severe inhibition of photosynthetic processes through light attenuation, and consequent oxygen depletion that disrupts ecological equilibrium. From a toxicological perspective, chronic MB exposure in humans has been linked to dermatological irritation, neurotoxic manifestations, and respiratory dysfunction.^10,11^

Given the ineffectiveness of conventional treatment methods-such as filtration, flocculation, and biological oxidation-against such pollutants, there is a critical need for advanced water purification technologies that can achieve complete degradation and mineralization of these contaminants.^12–14^ In this context, Advanced Oxidation Processes (AOPs) have emerged as highly promising due to their capability to generate reactive oxygen species (ROS) capable of oxidizing a wide range of organic molecules into environmentally benign products like CO_2_ and H_2_O.^15,16^ Among various AOPs, heterogeneous photocatalysis has attracted particular interest for being solar-driven, cost-effective, and environmentally friendly. Traditional photocatalysts such as zinc oxide (ZnO), titanium dioxide (TiO_2_), zinc sulphide (ZnS), cadmium sulphide (CdS), tin oxide (SnO_2_), and tungsten trioxide (WO_3_) have been extensively studied owing to their chemical stability, strong oxidative capability, and wide availability.^3,5^ However, their photoactivity is hindered by sluggish surface reactions that lead to rapid recombination of photogenerated charge carriers. Additionally, ZnO suffers from limited light-harvesting ability, as it primarily responds to the UV region, which constitutes only about 4% of the solar spectrum.^17–19^ Despite this limitation, ZnO has been widely employed in photocatalytic applications over the past few decades.

To overcome this intrinsic limitation, extensive research efforts have concentrated on enhancing visible-light photocatalytic activity through strategic bandgap engineering and surface modification approaches. Among these methodologies, the deposition of noble metal nanoparticles, particularly gold (Au) and silver (Ag), has emerged as a highly effective strategy.^19–22^ These plasmonic nanoparticles exhibit intense Surface Plasmon Resonance (SPR) under visible light irradiation, facilitating the generation of high-energy “hot electrons”.^22,23^ These energetic electrons can be efficiently transferred into the conduction band of adjacent semiconductor materials, thereby enhancing charge carrier separation, suppressing electron–hole recombination processes, and amplifying the production of reactive oxygen species (ROS), including superoxide (˙O_2_^−^) and hydroxyl (˙OH) radicals, which serve as the primary oxidizing agents for pollutant degradation.^16,19,24^ Previous investigations have demonstrated the potential of metal–semiconductor composites for dye degradation. Agalya *et al.* reported photocatalytic degradation efficiencies of 68.7% for MB and 70.1% for tetracycline within 120 min using CuO nanoparticles, while Shkir *et al.* achieved superior performance with Ni/ZnO nanocomposites, demonstrating 94% MB degradation and 78% tetracycline degradation over 240 min.^25,26^ Despite these promising outcomes, there remains considerable scope for the development of more efficient visible-light-responsive photocatalysts capable of achieving faster pollutant degradation with improved reaction kinetics.

Building upon these findings and motivated by the potential of plasmonic enhancement, the present investigation focuses on the rational design, synthesis, and comprehensive photocatalytic evaluation of ZnO nanostructures decorated with Au and Ag nanoparticles for visible-light-driven degradation of TC and MB. ZnO nanostructures were specifically selected owing to their favorable two-dimensional morphology, which provides high surface area, shortened diffusion pathways for photogenerated charge carriers, and abundant catalytically active sites. The ZnO nanostructures were synthesized through a controlled two-step thermal treatment process employing glucose and urea as bio-derived structure-directing agents, followed by the strategic deposition of Au and Ag nanoparticles *via* a wet chemical reduction methodology using hydrazine hydrate as the reducing agent.

The resulting nanocomposites were systematically characterized using complementary analytical techniques, including powder X-ray diffraction (PXRD), X-ray photoelectron spectroscopy (XPS), field-emission scanning electron microscopy (FESEM), high-resolution transmission electron microscopy (HR-TEM), UV-vis spectroscopy, and Brunauer–Emmett–Teller (BET) surface area analysis to elucidate their structural, compositional, and morphological properties. The photocatalytic activity of the synthesized ZnO-based nanocomposites was systematically evaluated under visible-light irradiation through both individual and co-degradation experiments using TC and MB as model pharmaceutical and dye pollutants, respectively. In individual degradation studies, Au-ZnO achieved 98% MB degradation within 50 minutes and 99% TC degradation within 120 minutes, while Ag-ZnO showed comparable activity with 97% degradation in 120 minutes. In the co-degradation system, Au-ZnO exhibited superior dual removal efficiency, achieving 95% TC and 80% MB degradation within 120 minutes. Mass analysis confirmed the degradation pathways and the formation of key intermediates involved in the photocatalytic decomposition of both TC and MB. Radical scavenger studies confirmed that superoxide (˙O_2_^−^) and hydroxyl (˙OH) radicals are the primary reactive species driving the degradation process. This study establishes a versatile and multifunctional photocatalytic platform capable of efficiently degrading both pharmaceutical effluent and dye pollutants, individually as well as concurrently, thereby revealing a robust correlation between structure, properties, and photocatalytic performance in noble metal decorated ZnO nanostructures for sustainable wastewater remediation.

## 2. Experimental procedure

### 2.1. Synthesis of ZnO nanostructures (NSs)

ZnO NSs were synthesized *via* a facile, two-step calcination process.^27^ In a typical synthesis, a precursor solution was prepared by thoroughly stirring 5 g of glucose and 1 g of urea into 5 mL of a 0.1 M aqueous solution of zinc nitrate hexahydrate [Zn(NO_3_)_2_·6H_2_O] until a transparent solution was obtained. The mixture was then pre-calcined in a hot air oven at 160 °C for 6 h. This step facilitates the polymerization of glucose and the decomposition of urea, which releases ammonia gas and promotes the formation of a carbon foam intermediate.

The calcination protocol, particularly the pre-calcination temperature, was found to be a critical factor in controlling the final morphology of the product. While pre-calcination at 120 °C failed to form the desired carbon foam, successful foaming was observed at 140 °C and 160 °C (Fig. S1). The final calcination step was then tailored to the desired outcome. For instance, pre-calcination at 140 °C for 6 h followed by a final calcination at 500 °C for 10 h yielded a dense nanostructure (Fig. S2). Conversely, increasing the pre-calcination temperature to 160 °C for 6 h and the final calcination temperature to 600 °C for 10 h successfully generated a porous nanostructure (Fig. S3). We also found that direct calcination at 600 °C without the initial pre-calcination step failed to produce well-defined ZnO NSs, which is consistent with the findings of Zhou *et al.*^27^ After the final calcination, the resulting white product was cooled, washed with distilled water to remove impurities, and air-dried to yield the final porous ZnO nanostructures.

### 2.2. Synthesis of Ag- and Au-decorated ZnO nanocomposites (NCs)

Decoration of Ag and Au nanoparticles onto the ZnO nanostructure surfaces was accomplished through a solution-phase chemical reduction methodology. For Ag-ZnO synthesis, 50 mg of the as-synthesized ZnO NSs were dispersed in 12 mL of deionized water and subjected to ultrasonication for 45 min to ensure complete dispersion. Subsequently, 8 mL of 0.01 M AgNO_3_ solution was introduced dropwise under continuous magnetic stirring to achieve a final AgNO_3_ concentration of 0.004 M, without employing additional surfactants or capping agents. The resulting suspension was maintained under stirring for approximately 2.5 h to facilitate uniform adsorption of Ag^+^ ions onto the ZnO surface. The Ag^+^-loaded ZnO was subsequently recovered by centrifugation, washed twice with deionized water to remove excess AgNO_3_, and redispersed in 20 mL of deionized water for the reduction step.

Subsequently, 0.2 mL of 1 M hydrazine hydrate (N_2_H_4_·H_2_O, 98%) was added dropwise to the suspension under vigorous stirring at ambient temperature, initiating the *in situ* reduction of adsorbed metal ions to their metallic states. The formation of metallic silver was evidenced by the appearance of a light brown-colored precipitate, characteristic of Ag nanoparticle formation. The synthesis of Au-ZnO NCs followed an analogous protocol, with HAuCl_4_ substituted for AgNO_3_ as the noble metal precursor while maintaining identical concentrations and reaction conditions. The hydrazine hydrate reduction of Au^3+^ ions resulted in the formation of a characteristic violet-colored precipitate, confirming successful Au NP deposition on the ZnO surface.

**Scheme 1:**
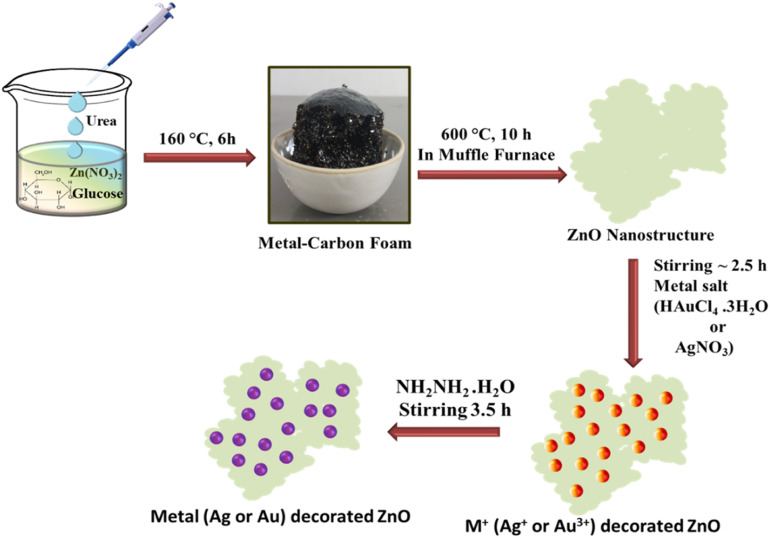
The synthesis procedure of ZnO NSs and Au-ZnO and Ag-ZnO nanocomposites (NCs).

The Au-ZnO NC was subjected to the same washing and drying procedures as described for the Ag-ZnO system. The complete synthetic methodology is illustrated in Scheme 1.

Hydrazine hydrate is highly toxic and volatile; therefore, all procedures were conducted in a well-ventilated fume hood with appropriate personal protective equipment (gloves, lab coat, and safety goggles). Any residual hydrazine in the filtrate was neutralized with dilute hydrogen peroxide solution (H_2_O_2_) before disposal, in accordance with standard laboratory safety protocols.

The obtained nanocomposite was collected by centrifugation and washed thoroughly with deionized water and ethanol (five to six times) to remove unreacted precursors and other by-products. The purified solid was then air-dried on a hot plate (∼60 °C) for 6 h to yield the final Au- or Ag-decorated ZnO nanocomposite, which was stored in an airtight container for subsequent characterization and photocatalytic studies.

### 2.3. Photocatalytic activity evaluation

The photocatalytic performance of pristine ZnO NSs and their noble metal-decorated analogues (Au-ZnO and Ag-ZnO) was systematically evaluated through the degradation of two representative contaminants: methylene blue (MB) and tetracycline (TC). Stock solutions of TC (2.0 × 10^−3^ M, 962 mg L^−1^) and MB (1.0 × 10^−5^ M, 3.2 mg L^−1^) were prepared in deionized water (50 mL) at room temperature, and their initial UV-vis absorption spectra were recorded to establish baseline absorbance values (*C*_0_) for kinetic analysis.

For each photocatalytic trial, predetermined catalyst loadings of 20 mg for TC and 50 mg for MB were introduced into the respective pollutant solutions. The solution pH and reaction time were varied during the experiments, as detailed in the corresponding sections. The suspensions were subjected to ultrasonication for 30 min to achieve homogeneous catalyst dispersion, followed by magnetic stirring in darkness for an additional 30 min to establish adsorption–desorption equilibrium. UV-vis spectra were acquired immediately after the dark equilibration to serve as the “dark” reference for monitoring photocatalytic degradation. The suspensions were then exposed to visible light irradiation using a 100 W tungsten filament lamp as the illumination source, while being continuously stirred. The irradiance at the reactor surface could not be directly measured due to instrumental limitations, which is acknowledged as a limitation when interpreting the photocatalytic performance. At predetermined time intervals, aliquots were withdrawn from the reaction mixture. These samples were centrifuged at 6000 rpm for 5 minutes to separate the photocatalyst from the liquid phase. The supernatants were analyzed using UV-vis spectroscopy to determine the residual concentration of the pollutant (*C*_*t*_). To maintain consistent reaction conditions, the separated photocatalyst was promptly reintroduced into the reaction vessel, followed by brief ultrasonication (2 min) to restore dispersion before resuming irradiation. The photocatalytic degradation was monitored until a plateau in absorbance was observed, indicating near-complete degradation of the pollutant.

The photocatalytic degradation efficiency (*D*%) was calculated using eqn (1):1
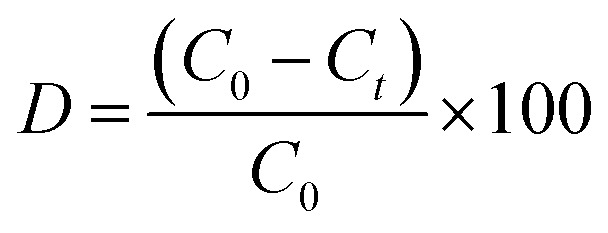
where *C*_0_ is the initial concentration of dye or antibiotic (mg L^−1^), and *C*_*t*_ is the concentration of dye or antibiotic at a given time *t* (in minutes).

All photocatalytic degradation experiments were performed in triplicate (*n* = 3), and the kinetic values reported correspond to mean ± standard deviation.

### 2.4. Regeneration and reusability of the catalyst

After each adsorption–photocatalysis cycle, the catalyst was recovered by centrifugation and thoroughly washed with deionized water to remove residual adsorbed dye molecules. The solid was repeatedly rinsed until a neutral pH was achieved, followed by drying at 60 °C for 6 h prior to reuse. This regeneration procedure was repeated for four successive cycles. The regenerated catalysts retained approximately 85–90% of their initial photocatalytic activity, demonstrating that the recovery process effectively restores accessible active sites and preserves the structural integrity of the photocatalysts.

## 3. Results and discussion

This study details the successful synthesis of pristine zinc oxide NSs and their subsequent surface decoration with Au and Ag NPs to create metal-ZnO nanocomposites. The precise synthesis of these hybrid nanomaterials aims to harness synergistic effects for enhanced performance. To comprehensively characterize these materials and elucidate the impact of noble metal incorporation on their physicochemical properties, the following analytical techniques have been meticulously performed and are presented in the subsequent sections.

### 3.1. X-ray diffraction (XRD) analysis of ZnO and metal-decorated ZnO NCs

Powder X-ray diffraction (PXRD) analysis was employed to investigate the crystallinity, phase purity, and structural modifications in ZnO and its noble metal-decorated nanocomposites.

#### 3.1.1. Crystallinity and phase purity

The diffraction pattern of pure ZnO NSs (Fig. S4) revealed well-defined peaks at 2*θ* values of 31.86°, 34.51°, 36.30°, 47.67°, 56.71°, 62.97°, 66.48°, 68.15°, 69.26°, 72.67°, and 77.04°. These correspond to the (100), (002), (101), (102), (110), (103), (200), (112), (201), (004), and (202) planes, respectively. These reflections align well with the hexagonal wurtzite ZnO phase (JCPDS card no. 79-0205), confirming the successful formation of phase-pure ZnO NSs without the presence of secondary phases.^19,28,29^

**Fig. 1:**
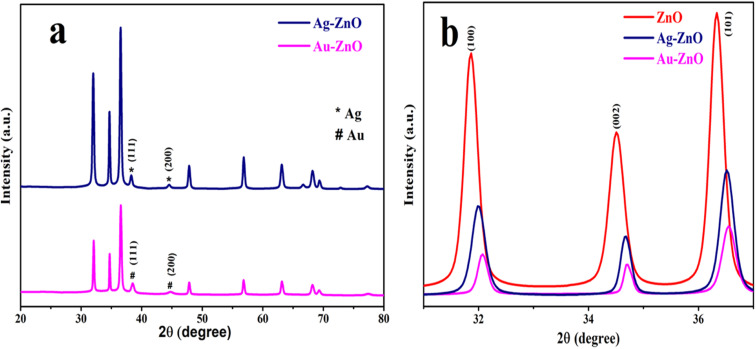
PXRD pattern of (a) Au-ZnO and Ag-ZnO and (b) enlarged view of peaks of the (100), (002), and (101) peaks.

Upon decoration with noble metals, the PXRD patterns of Au-ZnO and Ag-ZnO NCs (Fig. 1a) retained the characteristic ZnO wurtzite phase, while additional peaks emerged due to the presence of metallic nanoparticles. For the Au-ZnO nanocomposite, distinct diffraction peaks were observed at 38.50° and 44.76°, which are indexed to the (111) and (200) planes of face-centered cubic (fcc) metallic gold (JCPDS card no. 65-2870). This observation confirms the successful *in situ* reduction and deposition of metallic Au^0^.^29,30^ Similarly, the Ag-ZnO NCs exhibited peaks at 38.27° and 44.53°, corresponding to the (111) and (200) planes of metallic silver (JCPDS card no. 01-1167), thereby verifying the successful deposition of Ag^0^ onto the ZnO matrix.^28,31^

#### 3.1.2. Structural modifications and lattice strain

Notably, slight shifts in the ZnO diffraction peaks were observed in both Au-ZnO and Ag-ZnO samples compared to pristine ZnO, as illustrated in the magnified region of Fig. 1b. These peak shifts toward higher 2*θ* values indicate lattice contraction, attributed to interfacial strain effects arising from the strong adhesion and electronic interactions between the surface-deposited noble metal nanoparticles and the underlying ZnO lattice. The observed shifts reflect modifications in *d*-spacing and localized lattice distortion at the metal–semiconductor interfaces, consistent with the formation of intimate heterojunctions rather than bulk lattice incorporation.^18,26^

Furthermore, noticeable reductions in the peak intensity and systematic peak broadening (increased FWHM) were observed for the noble metal-decorated samples relative to pristine ZnO. As confirmed by quantitative analysis using the Debye–Scherrer and Williamson–Hall methods, this peak broadening arises from reduced crystallite size (from 19.89 nm to 11.06 nm for Au-ZnO and 17.07 nm for Ag-ZnO) and enhanced microstrain induced by surface stress at the metal-ZnO interfaces.

#### 3.1.3. Crystallite size, microstrain, and dislocation density analysis

The average crystallite sizes were determined using the Debye–Scherrer eqn (2):^13,18^

2 *D* = *kλ*/(*β* cos *θ*)

where *λ* is the X-ray wavelength (1.5406 Å for Cu Kα radiation), *β* is the full width at half maximum (FWHM) in radians, *θ* is the Bragg angle, and *k* is the shape factor (0.9). The calculated crystallite sizes were 19.89 nm for pristine ZnO, 11.06 nm for Au-ZnO, and 17.07 nm for Ag-ZnO, demonstrating a systematic reduction in crystallite dimensions upon noble metal decoration.

To account for microstrain contributions and validate these findings, crystallite sizes were additionally determined using the Williamson–Hall (W–H) method (3):^5^3
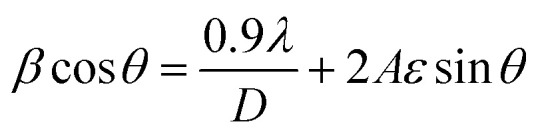
where *D* represents the crystallite size and *ε* denotes the microstrain. Linear plots of *β* cos *θ versus* 4 sin *θ* (Fig. S5a–c) yielded crystallite sizes of 31.51 nm (ZnO), 39.50 nm (Ag-ZnO), and 29.37 nm (Au-ZnO). These values exceed those from the Scherrer equation due to strain decoupling, confirming significant microstrain contributions to peak broadening.^18^

Microstrain values were calculated using the following eqn (4):^15^

4 *ε* = *β*/(4 tan *θ*)

The strain values (Table 1) increased substantially for both noble metal-decorated samples relative to pristine ZnO, consistent with interfacial stress and lattice distortion induced by metal nanoparticle deposition.^16^ Correspondingly, the dislocation density (*δ*), representing crystallographic defect concentration, was estimated using the following eqn (5):^18^5
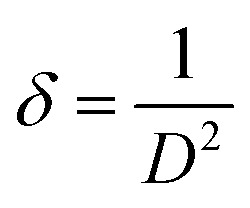


**Table 1:** Physical parameters of pure ZnO nanostructures and Au- and Ag-decorated ZnO

Sample	Particle size Debye Scherrer's equation (nm)	Particle size W–H formula (nm)	Microstrain (*ε*) × 10^−3^	Dislocation density (*δ*) × 10^−3^ (nm^−2^)
ZnO	19.89	31.51	3.4101	2.885
Ag-ZnO	17.065	39.50	3.6941	3.2925
Au-ZnO	11.064	29.37	4.1014	4.273

Enhanced dislocation densities were observed for both Au-ZnO and Ag-ZnO samples (Table 1), indicating increased structural defects associated with noble metal incorporation.^13,32^ These structural modifications collectively suggest successful metal–semiconductor interface formation with concomitant lattice perturbations that may influence electronic properties and photocatalytic activity.

Collectively, the PXRD analysis confirms the successful synthesis of pure ZnO nanostructures and the subsequent deposition of Au and Ag nanoparticles in their metallic form on the ZnO surface. The observed structural changes, such as peak shifting, reduction in the crystallite size, increased lattice strain, and higher dislocation density, highlight the significant influence of noble metal deposition on the ZnO lattice. These modifications are expected to significantly enhance the photocatalytic performance of the resulting nanocomposites.

PXRD patterns recorded for ZnO NSs synthesized *via* calcination at 500 °C are shown in Fig. S6. Both temperatures yielded similar diffraction features, confirming the formation of crystalline ZnO nanostructures. However, synthesis at 600 °C was adopted for further studies, as higher calcination temperature improved the nanostructural porosity without altering the phase composition. This enhancement in porosity at elevated temperature is consistent with the trend reported by Zhou *et al.*^27^

### 3.2. Raman analysis

**Fig. 2:**
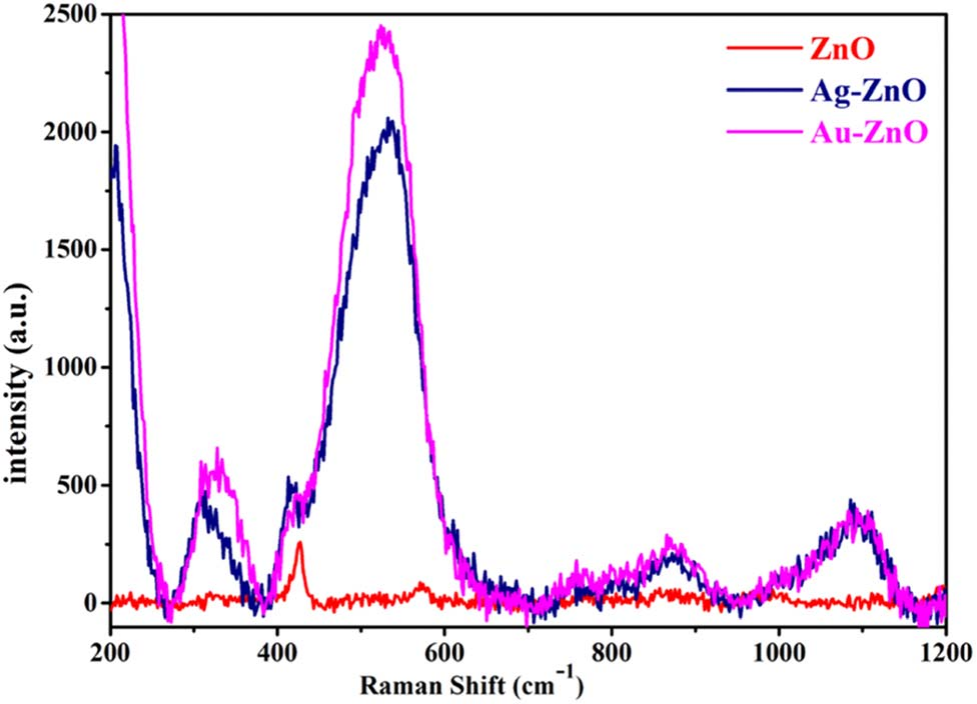
Raman spectra of ZnO NSs and Au-ZnO and Ag-ZnO NCs.

Raman spectroscopy was employed to investigate the vibrational characteristics and structural modifications of pristine ZnO and noble metal-decorated composites (Au-ZnO and Ag-ZnO) (Fig. 2). The spectra exhibit characteristic phonon modes of the hexagonal wurtzite ZnO structure, with the E_2_(low) mode at ∼329 cm^−1^ and the E_2_(high) mode at 424 cm^−1^ for pristine ZnO, confirming excellent phase purity and crystalline quality.^33^ The peak at ∼511 cm^−1^, assigned to the A_1_(TO) mode, is associated with intrinsic lattice defects including zinc interstitials and oxygen vacancies.^34^

Upon noble metal decoration, the A_1_(TO) mode exhibited systematic blue shifts to 526 cm^−1^ (Au-ZnO) and 532 cm^−1^ (Ag-ZnO), indicating localized compressive strain and enhanced metal–oxygen bonding interactions at the heterojunction interfaces.^32,34^ Conversely, the E_2_(high) mode demonstrated red shifts to 419 cm^−1^ (Au-ZnO) and 413 cm^−1^ (Ag-ZnO), consistent with tensile strain development and phonon softening in the vicinity of the metal–semiconductor interfaces.^33^ Both the A_1_(TO) and E_2_(high) modes displayed pronounced broadening in the decorated samples, reflecting increased lattice distortion, enhanced defect-phonon coupling mechanisms, and reduced phonon coherence lengths. These complementary spectral modifications provide direct evidence for successful noble metal NP anchoring and the establishment of intimate metal-ZnO interfacial interactions.

### 3.3. Optical properties and band gap analysis

**Fig. 3:**
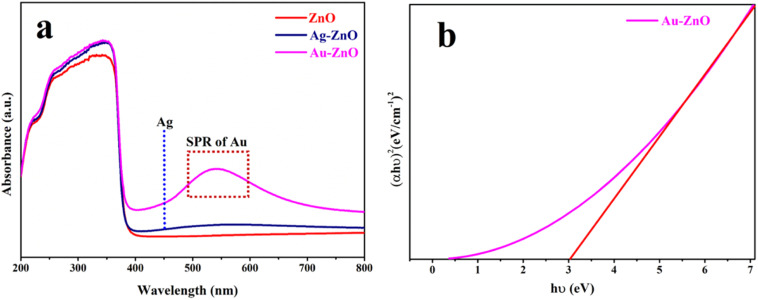
(a) UV-vis diffuse reflectance spectra of ZnO, Au-ZnO, and Ag-ZnO and (b) Tauc plot for estimating the optical band gap of Au-ZnO.

The optical absorption characteristics of the synthesized materials were examined using UV-visible diffuse reflectance spectroscopy (UV-vis DRS), as presented in Fig. 3a. Pristine ZnO displayed intense absorption in the ultraviolet region (250–380 nm), characteristic of intrinsic band-to-band transitions in the wurtzite ZnO structure.^35^ The optical band gap energy (*E*_g_), determined *via* Tauc plot (*αhν vs. hν*) analysis, was calculated to be 3.19 eV (Fig. S7a), consistent with reported literature values.^5^

Noble metal decoration significantly modified the optical absorption behavior, leading to an apparent red shift in the absorption edge, with band-gap values of 3.01 eV for Au-ZnO (Fig. 3b) and 3.08 eV for Ag-ZnO (Fig. S7b). This shift is most likely due to plasmonic absorption tails generated by the noble metal nanoparticles together with defect-related sub-band states, which are well-known influences in DRS-derived Tauc analyses of metal–semiconductor composites.^16,34^ Notably, the spectra revealed distinct surface plasmon resonance (SPR) features: a broad absorption band spanning 400–520 nm for Ag-ZnO and a more pronounced SPR peak centered at approximately 540 nm for Au-ZnO, providing direct evidence for successful metallic nanoparticle decoration.^22^ The more substantial band structure modification and stronger SPR response observed for Au-ZnO can be attributed to superior plasmonic coupling and enhanced interfacial charge transfer between Au nanoparticles and the ZnO matrix. This stronger interaction promotes hot electron injection from plasmonic Au into the ZnO conduction band, thereby improving charge carrier separation efficiency and suppressing recombination processes.^23^

### 3.4. Fourier Transform Infrared (FTIR) spectroscopy analysis

Fourier Transform Infrared (FTIR) spectroscopy, as presented in Fig. S8, was performed to elucidate the vibrational characteristics and surface interactions of the ZnO, Au-ZnO, and Ag-ZnO nanocomposites. All samples exhibited characteristic Zn-O stretching vibrations (450–540 cm^−1^), confirming the wurtzite ZnO structure.^5,36^ Notably, red-shifts in Zn-O modes upon noble metal decoration (*e.g.*, Au-ZnO: 477, 650, 682, and 774 cm^−1^; Ag-ZnO: 442, 542, 615, 778, and 921 cm^−1^) indicated significant electronic and structural interactions at the metal–ZnO interface.^23,32,37^ Subtle shifts in the 1385–1630 cm^−1^ C=O band (residual organics) suggested surface chemical modifications.^5^ The O–H stretching band (adsorbed moisture), shifting from 3431 cm^−1^ (pristine) to 3440 cm^−1^ (Au-ZnO) and 3436 cm^−1^ (Ag-ZnO) further underscored intimate surface interactions and altered hydrogen bonding.^38^ These collective spectral changes provide compelling evidence for successful surface functionalization and profound alterations in the chemical environment.

### 3.5. X-ray Photoelectron Spectroscopy (XPS) analysis

X-ray photoelectron spectroscopy (XPS) was employed to investigate the surface elemental composition, chemical states, and electronic environments of pristine ZnO NSs and their noble metal-decorated counterparts (Au-ZnO and Ag-ZnO). Both survey and high-resolution spectra provided detailed insights into the binding energies and surface chemical modifications induced by the incorporation of Au and Ag NPs. The XPS spectra were calibrated by referencing the C 1s peak corresponding to C–C/C–H bonds to 284.8 eV. For ZnO, a uniform shift of −1.0 eV was applied to correct surface charging, whereas Au-ZnO and Ag-ZnO samples required negligible adjustment due to improved conductivity from noble-metal decoration.

The XPS survey spectrum of pristine ZnO (Fig. S9a) confirmed the presence of Zn and O as the principal elements, along with minor adventitious carbon contamination. The high-resolution Zn 2p spectrum (Fig. S9b) displayed two well-defined peaks at 1022.34 eV (Zn 2p_3/2_) and 1045.44 eV (Zn 2p_1/2_), with a spin–orbit splitting of approximately 23.10 eV, consistent with the Zn^2+^ oxidation state in ZnO lattice sites.^22^ The O 1s region (Fig. S9c) was deconvoluted into two components: a primary peak at 531.18 eV attributed to lattice oxygen (Zn-O), and a secondary feature at 532.94 eV ascribed to surface hydroxyl groups or adsorbed oxygen species.^15^ The presence of these non-lattice oxygen species suggests oxygen vacancies or defect states, which are known to enhance photocatalytic activity by facilitating charge separation and reactive oxygen species (ROS) formation.

**Fig. 4:**
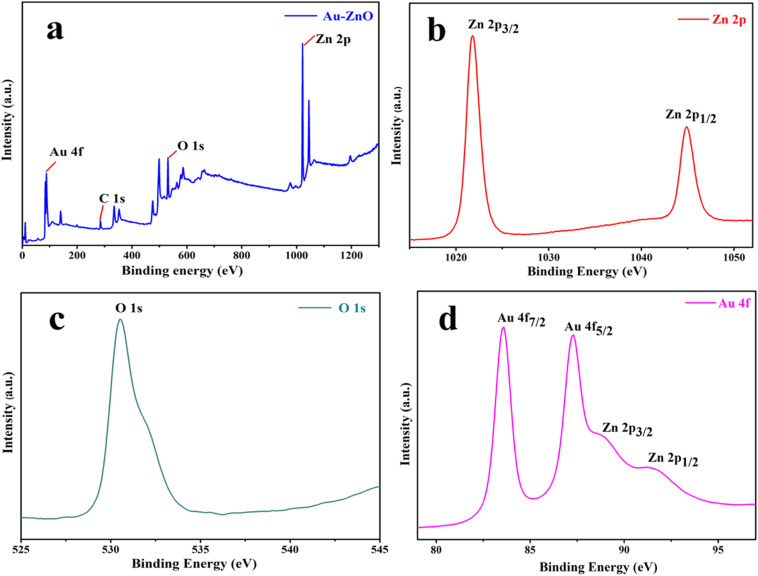
XPS analysis of Au-ZnO NCs: (a) survey spectrum confirming the presence of Zn, Au, O, and C; (b) Zn 2p; (c) O 1s; (d) Au 4f.

For the Au-ZnO NCs, the survey spectrum (Fig. 4a) revealed peaks corresponding to Zn, O, Au, and C, confirming the successful surface loading of gold nanoparticles. The Zn 2p signals (Fig. 4b) were observed at 1021.30 eV and 1044.90 eV, showing a slight shift to lower binding energies compared to pristine ZnO.^28,31^ This shift indicates electronic interaction and charge redistribution at the metal–semiconductor interface, likely due to the formation of a Schottky junction. The O 1s spectrum (Fig. 4c) exhibited peaks at 530.50 eV (lattice oxygen) and 531.88 eV (defect-related oxygen), with an increased relative intensity of the latter, suggesting that Au incorporation promotes surface defect formation.^33^ The high-resolution Au 4f spectrum (Fig. 4d) showed characteristic peaks at 83.54 eV (Au 4f_7/2_) and 87.25 eV (Au 4f_5/2_), confirming the metallic state of Au^0^.^33,39^ No peaks corresponding to oxidized Au^3+^ species were observed, indicating complete reduction of Au ions during synthesis. A minor overlap with the Zn 3p region is acknowledged as a common artifact in XPS measurements.^40^

**Fig. 5:**
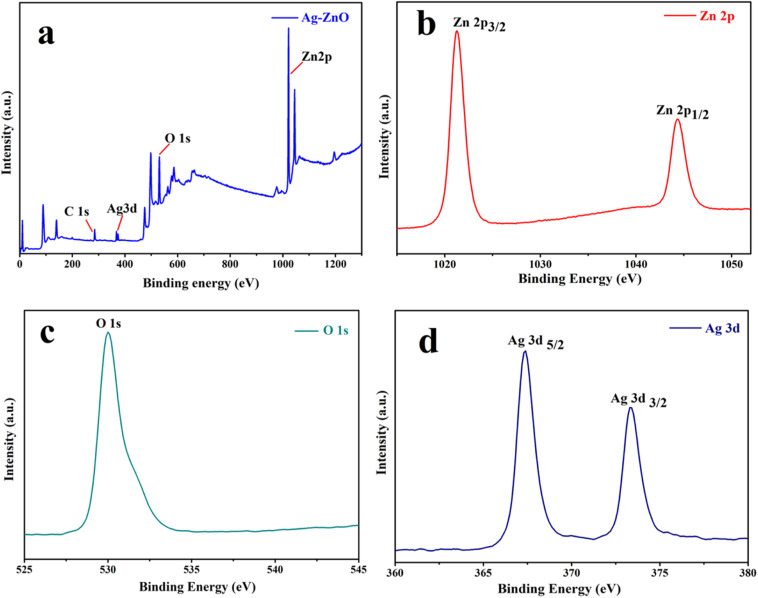
XPS analysis of Ag-ZnO NCs: (a) survey spectrum confirming the presence of Zn, Ag, and O; (b) high-resolution Zn 2p spectrum; (c) O 1s spectrum indicating Zn-O bonds; (d) high-resolution Ag 3d spectrum.

In the case of the Ag-ZnO nanocomposite, the survey spectrum (Fig. 5a) displayed signals from Zn, O, Ag, and C. The Zn 2p doublet (Fig. 5b) appeared at 1021.74 eV and 1044.89 eV, slightly shifted relative to pristine ZnO, again indicating electronic coupling between Ag nanoparticles and the ZnO surface. The O 1s region (Fig. 5c) showed deconvoluted peaks at 530.0 eV (lattice oxygen) and 531.44 eV (surface oxygen species), consistent with the presence of oxygen vacancies or adsorbed molecular species, as seen in the Ag-ZnO sample. The high-resolution Ag 3d spectrum (Fig. 5d) revealed peaks at 367.32 eV (Ag 3d_5/2_) and 373.34 eV (Ag 3d_3/2_), with a spin–orbit separation of ∼6.0 eV, which is characteristic of metallic silver (Ag^0^).^19,28,41^ The absence of any Ag^+^ peaks indicates complete reduction of Ag^+^ ions. A slight negative shift in the binding energy relative to bulk Ag is attributed to nanoscale size effects and interfacial interactions with ZnO.

A summary of the observed binding energies is provided in Table S1. In both Au-ZnO and Ag-ZnO, the small but consistent shifts in Zn 2p binding energies relative to pristine ZnO reflect subtle electronic interactions at the metal–semiconductor interface. Notably, the defect-related O 1s component does not show any significant effect in intensity upon noble-metal decoration, suggesting that the enhanced visible-light photocatalytic performance arises mainly from plasmon-induced charge separation and interfacial electronic effects.

### 3.6. Morphological analysis

**Fig. 6:**
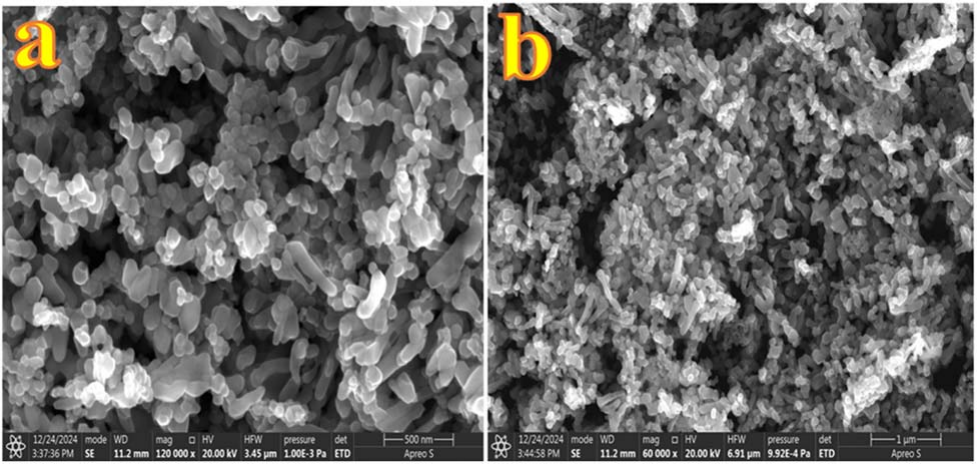
(a and b) FESEM images of ZnO nanostructures at high (a) and low resolutions (b).

To analyse the surface morphology and microstructural features of the ZnO synthesized nanostructures and their metal-decorated nanocomposites, Au-ZnO and Ag-ZnO, Field Emission Scanning Electron Microscopy (FESEM) was employed. Additionally, Energy Dispersive X-ray Spectroscopy (EDS) was utilized to determine the elemental composition and distribution of Zn, O, Au, and Ag within the samples. The FESEM images of pure ZnO (Fig. 6a and b) exhibit a hierarchical lamellar nanostructure morphology. At higher magnification (Fig. 6a), the surface is composed of compactly arranged and interconnected ZnO nanoparticles, which collectively form a layered architecture. The lower magnification image (Fig. 6b) confirms the extended, layered texture of these nanostructures across the surface. To examine whether prolonged synthesis affects the morphology, the reaction time was extended from 10 h to 15 h. FESEM analysis of the sample obtained after 15 h (Fig. S10) showed no appreciable morphological change compared with the standard 10 h sample, indicating that the optimized structure is already achieved within 10 h.

**Fig. 7:**
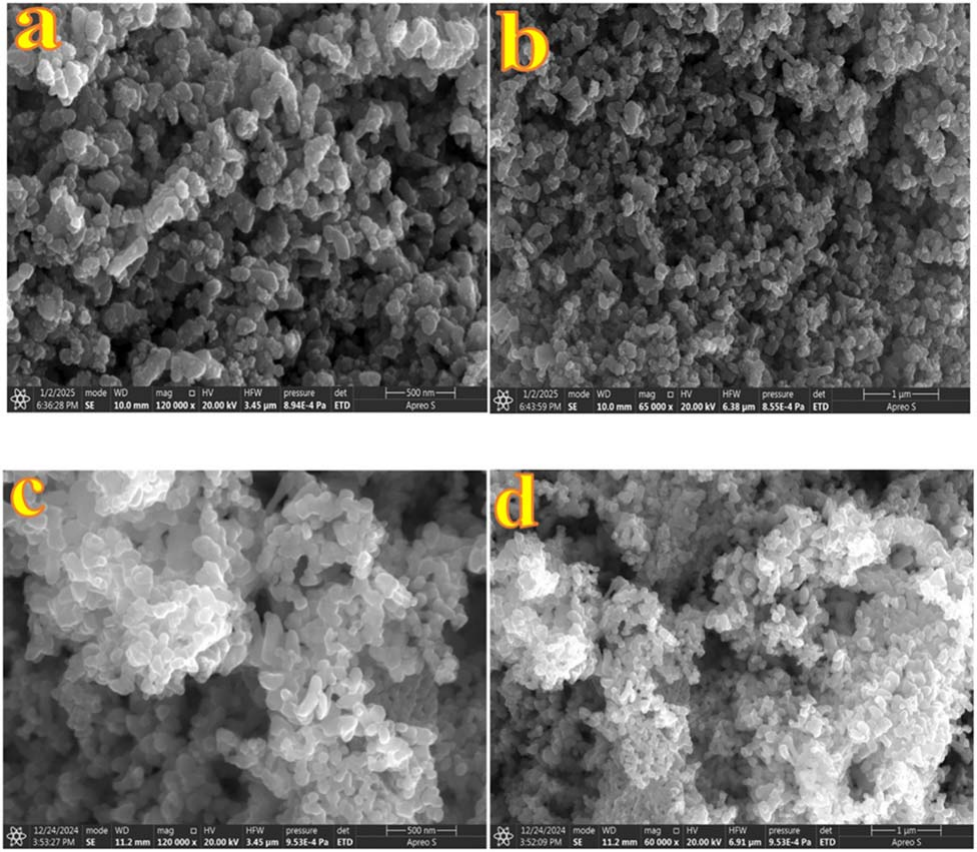
FESEM images of (a and b) Au-ZnO and (c and d) Ag-ZnO nanocomposites at low and high magnifications, respectively.

The FESEM images of Au-decorated ZnO NCs (Fig. 7a and b) clearly demonstrate morphological modifications compared to bare ZnO. The high-magnification image (Fig. 7a) reveals the presence of uniformly distributed spherical Au NPs embedded on the surface of ZnO nanostructures. These Au NPs appear as discrete, brighter contrast features, indicating successful surface decoration. The incorporation of Au induces localized distortion at the sheet edges, leading to a comparatively rougher texture and increased surface heterogeneity. The lower magnification image (Fig. 7b) supports the overall homogeneity of Au distribution across the nanostructure matrix. FESEM images of Ag-decorated ZnO NCs (in Fig. 7c and d) exhibit a denser and more agglomerated nanostructure morphology compared to pure ZnO. Although distinct Ag nanoparticles are not prominently visible, the noticeable textural modifications confirm their successful decoration.

The average crystallite sizes of Au and Ag NPs were estimated to be 11.06 nm and 17.07 nm, respectively, based on PXRD data using the Debye–Scherrer equation. The smaller size and higher dispersion of Au compared to Ag can be attributed to the rapid reduction kinetics of Au^3+^ ions by hydrazine hydrate, leading to a higher nucleation rate and the formation of numerous small nuclei.^42^ In contrast, the comparatively slower reduction of Ag^+^ ions results in fewer nucleation sites, allowing for the growth of comparatively larger particles. These morphological distinctions are anticipated to play a significant role in modulating the catalytic activity, with smaller, more uniformly distributed Au NPs offering enhanced surface reactivity and charge carrier dynamics.

Energy-dispersive X-ray spectroscopy (EDS) was employed to define the elemental composition and spatial distribution of the synthesized nanomaterials. For pure ZnO nanostructures, the elemental mapping (Fig. S11a–c) confirms a uniform distribution of Zn and O elements across the surface, with no observable contamination. The atomic composition was calculated to be Zn (38.81%), O (39.74%), and C (21.45%), where the detected carbon is attributed to residual precursors such as glucose and urea used during synthesis (Fig. S11d). In the case of Au-ZnO NCs (Fig. S12a–d), the EDS elemental maps demonstrate the homogeneous co-distribution of Zn, O, and Au throughout the material.

The EDS spectrum (Fig. S12e) shows additional peaks, corresponding to the Au-Mα, Mβ, and Mγ transitions, along with Zn-K and O-K signals. The atomic percentages were C (32.74%), O (36.22%), Zn (28.87%), and Au (2.17%), ensuring the efficient incorporation of Au nanoparticles into the ZnO matrix. Similarly, for Ag-ZnO NCs (Fig. S13a–d), the elemental maps confirm the presence and co-distribution of Zn, O, and Ag. The EDS spectrum (Fig. S13e) exhibits characteristic Ag-Lα peaks at 3.19 keV along with Zn-K and O-K peaks. The corresponding atomic composition reveals C (67.56%), O (24.53%), Zn (7.45%), and Ag (0.46%). The comparatively lower atomic percentage of Ag relative to Au suggests a reduced deposition density, which correlates well with the morphological differences observed in FESEM images. The observed X-ray energy values for Zn-K, O-K, Au-M, and Ag-L series in all spectra align with standard literature-reported values affirming the accuracy of the EDS analysis.^36,38,43,44^ Additionally, the absence of impurity-related peaks indicates high purity of the synthesized nanomaterials, making them viable candidates for advanced photocatalytic applications.

High-resolution transmission electron microscopy (HRTEM) was employed to investigate the decoration of Ag and Au nanoparticles on the surface of ZnO. This technique enabled direct visualization of the lattice fringes and interplanar spacing, confirming the crystalline nature and successful incorporation of Au and Ag onto the ZnO surface.

**Fig. 8:**
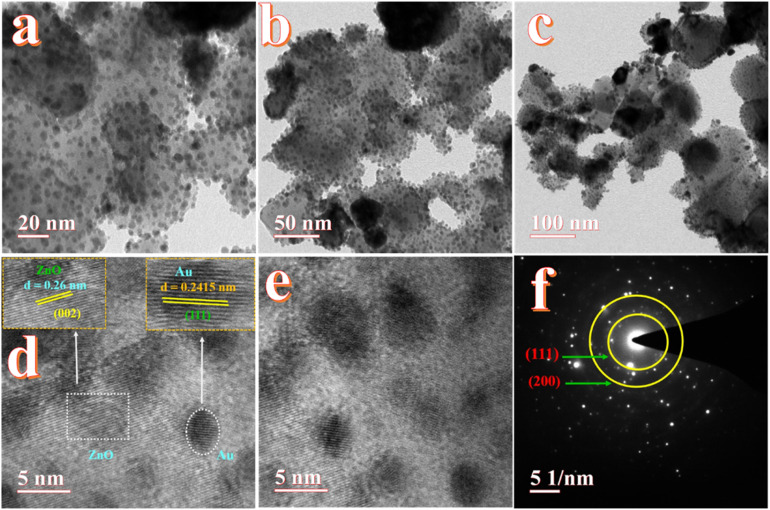
HR-TEM images of Au-ZnO (a–c) high to low resolution, (d and e) *d*-spacing calculation of Au-ZnO and (f) SAED patterns.

The TEM images in Fig. 8a–c illustrate the morphology of the Au-ZnO NCs at progressively decreasing magnifications. As shown in Fig. 8a and b the nanocomposite is composed of aggregated, irregularly shaped primary nanoparticles. Within these aggregates, smaller high-contrast nanoparticles corresponding to Au are observed in close association with the larger, lower-contrast regions attributed to the ZnO matrix. The Au NPs exhibit sizes in the range of 10–30 nm and appear as high contrast features due to their higher atomic number, while the surrounding ZnO matrix appears lighter under identical imaging conditions. Fig. 8c presents a broader view of the agglomerated architecture, confirming a typical nanoscale composite structure with uniformly distributed Au NPs on the ZnO surface.

A representative HRTEM image is shown in Fig. 8d. Well-defined lattice fringes are visible, confirming the crystalline nature of both components. The inset corresponding to the ZnO region (marked by a dashed rectangle) displays a measured interplanar spacing of approximately 0.26 nm, which matches well with the (002) plane of the hexagonal wurtzite ZnO phase.^22,45^ The lattice fringes associated with a single Au nanoparticle (highlighted by a dashed oval) exhibit an interplanar distance of 0.2415 nm, compatible with the (111) plane of face-centered cubic (fcc) Au.^22,44^ The intimate contact observed between the Au nanoparticle and the ZnO matrix suggests the formation of a well-defined heterointerface, which is often a prerequisite for enhanced interfacial charge transfer and synergistic behavior in photocatalytic systems. An additional HRTEM micrograph (Fig. 8e) further corroborates the presence of crystalline Au NPs embedded within or adhered to the ZnO matrix. The contrast difference between the two phases remains evident, supporting the conclusion of successful surface decoration of Au on ZnO. These microstructural features are representative of the entire sample and confirm high crystallinity and well-integrated nature of the composite.

The selected area electron diffraction (SAED) pattern, presented in Fig. 8f, was obtained from an area containing multiple Au-ZnO NCs and provides further confirmation of their polycrystalline nature and phase composition. The pattern displays a set of well-defined, concentric diffraction rings, characteristic of randomly oriented nanocrystals. The prominent rings labeled in the pattern correspond to the (111) and (200) crystallographic planes of FCC Au. The calculated *d*-spacings from these rings are consistent with standard values for metallic gold. The sharpness and continuity of the diffraction rings indicate good crystallinity of both the Au and ZnO phases within the nanocomposite material.^46^

**Fig. 9:**
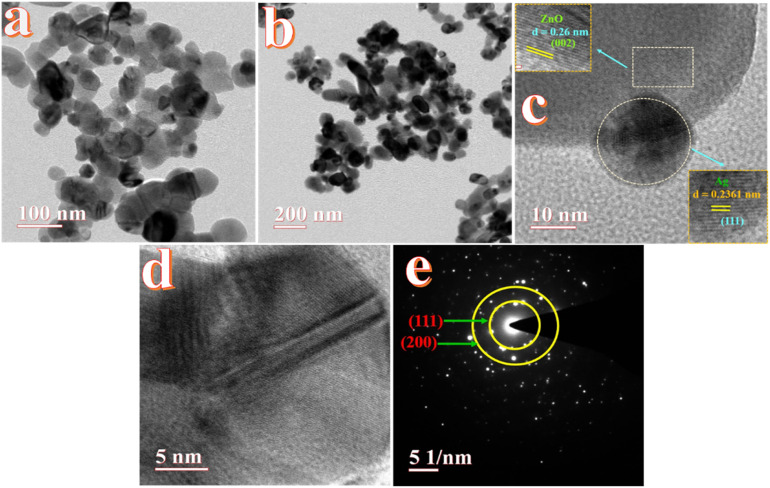
HR-TEM analysis of Ag-decorated ZnO NCs: (a) high and (b) low magnification, (c and d) *d*-spacing calculation of Ag-ZnO and (e) SAED pattern.

TEM images in Fig. 9a and b illustrate the morphology of Ag-ZnO NSs at different magnifications. The micrographs show the presence of spherical Ag nanoparticles randomly distributed across the ZnO surface. These Ag NPs exhibit darker contrast due to their higher atomic number, while the ZnO matrix appears with relatively lower contrast under identical imaging conditions. The observed Ag NPs range in size from 15 to 40 nm, indicating successful surface decoration and relatively uniform dispersion.

High-resolution TEM images (Fig. 9c and d) reveal well-resolved lattice fringes corresponding to both ZnO and Ag phases. The interplanar spacing measured for the ZnO region is approximately 0.26 nm, which matches the (002) plane of hexagonal wurtzite ZnO.^19,22,31^ In contrast, the Ag NPs exhibit a lattice spacing of 0.2361 nm, assigned to the (111) plane of face-centered cubic (fcc) silver.^22,31,47^ The clear visibility of these lattice fringes confirms the high crystallinity of both components and suggests good structural integration between Ag and ZnO. The SAED pattern shown in Fig. 9e further supports these findings. The diffraction rings are indexed to the (111) and (200) planes of fcc Ag, confirming its polycrystalline nature.^48,49^ The absence of additional diffraction rings from impurity phases indicates the structural purity of the synthesized Ag-ZnO NCs.

### 3.7. Brunauer–Emmet–Teller surface analysis

**Fig. 10:**
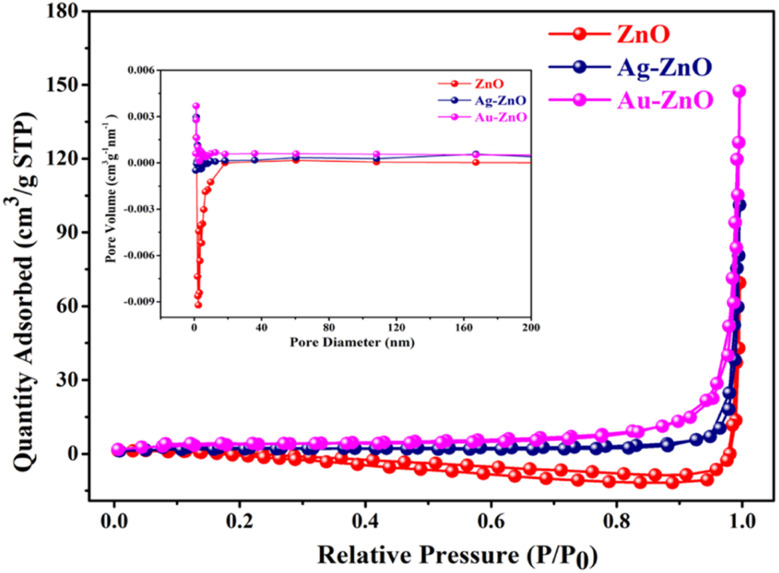
Nitrogen adsorption–desorption isotherms of ZnO NSs and Ag-ZnO and Au-ZnO NCs (inset: pore size distribution profiles, using the Barrett–Joyner–Halenda (BJH) method).

Nitrogen adsorption–desorption isotherms were employed to characterize the surface area and pore structure of the synthesized materials, as depicted in Fig. 10. All samples—bare ZnO, Au-ZnO, and Ag-ZnO NCs—exhibited Type IV isotherms with well-defined H3 hysteresis loops, indicative of mesoporous structures in accordance with IUPAC classification. Among the materials studied, the Au-ZnO NC exhibited the highest nitrogen uptake, corresponding to the largest Brunauer–Emmett–Teller (BET) specific surface area of 13.64 m^2^ g^−1^. A progressive increase in surface area was observed in the order ZnO (2.88 m^2^ g^−1^) < Ag-ZnO (6.97 m^2^ g^−1^) < Au-ZnO,^16,34^ reflecting the impact of noble metal deposition on the surface structure. This trend was further supported by a substantial increase in total pore volume, from 0.024 cm^3^ g^−1^ for ZnO to 0.123 cm^3^ g^−1^ for Au-ZnO.^10,43^

Concurrently, the average pore diameter shows a moderate increase from 32.70 nm for pure ZnO to 40.12 nm for Ag-ZnO, with Au-ZnO displaying an intermediate value of 36.06 nm. All the samples display mesoporous characteristics, consistent with the trend reported by Onkani *et al.*^34^ The pore size distribution profiles, derived using the Barrett–Joyner–Halenda (BJH) method, are presented in the inset of Fig. 10. A detailed summary of surface area, pore volume, and pore diameter for all samples is provided in Table 2.

**Table 2:** Details of BET surface area, total pore volume, and average pore diameter of ZnO, Ag-ZnO and Au-ZnO determined from N_2_ adsorption–desorption isotherms

Prepared materials	BET surface area (m^2^ g^−1^)	Avg. pore diameter (nm)	Pore volume (cm^3^ g^−1^)
ZnO	2.8809	32.70	0.024
Ag-ZnO	6.9687	40.12	0.069
Au-ZnO	13.643	36.06	0.123

### 3.8. Photocatalytic degradation studies

#### 3.8.1. Photocatalytic degradation of tetracycline hydrochloride (TC)

The photocatalytic performance of pristine ZnO nanostructures and noble metal-decorated composites (Au-ZnO and Ag-ZnO) was systematically evaluated for tetracycline hydrochloride (TC) degradation under visible light irradiation. TC degradation was monitored *via* UV-vis spectrophotometry by tracking the characteristic absorption maximum at *λ*_max_ = 358 nm.^20^

Initial experiments involved dispersing 20 mg of each photocatalyst in 50 mL of TC solution (2.0 × 10^−3^ M), followed by a 30-minute dark equilibration period to establish adsorption–desorption equilibrium. During this phase, negligible TC adsorption was observed, confirming that subsequent concentration changes were attributable to photocatalytic degradation rather than physical adsorption processes. Time-resolved UV-vis spectra of Au-ZnO, Ag-ZnO and ZnO (Fig. S14a–c) revealed a systematic decline in the absorbance intensity accompanied by a subtle red shift upon visible light exposure. This spectral evolution indicates progressive TC mineralization, while the red shift suggests π–π stacking interactions between TC molecules and surface-active sites (oxygen vacancies and hydroxyl groups) on the ZnO-based catalysts. These interactions enhance π-conjugation and facilitate interfacial charge transfer processes crucial for efficient degradation.^50^

**Fig. 11:**
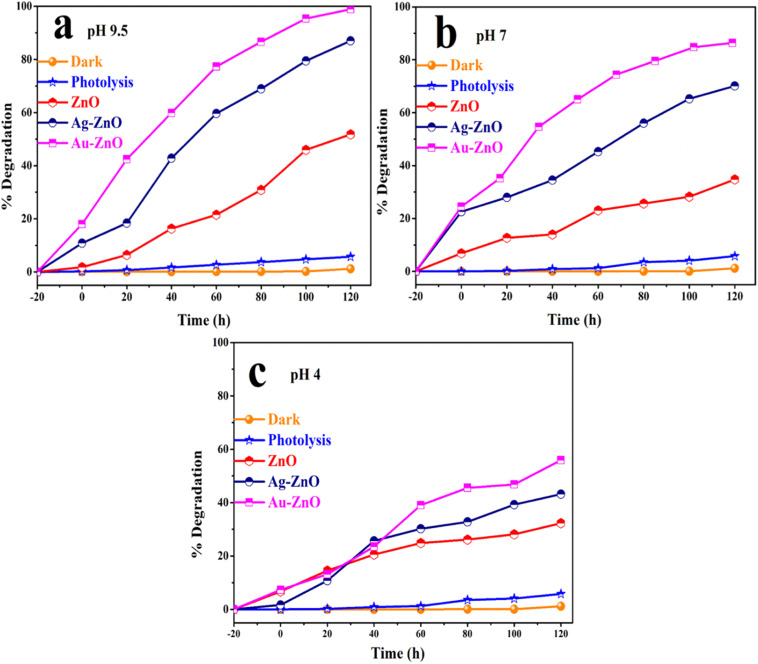
Photocatalytic degradation efficiency of tetracycline under visible light using ZnO, Au-ZnO, and Ag-ZnO at different pH values: (a) pH 9.5, (b) pH 7.0, and (c) pH 4.0. Control and dark experiments are included for comparison [catalyst: 20 mg, TC: 2 × 10^−3^ M, time: 120 min. at room temperature].

**Fig. 12:**
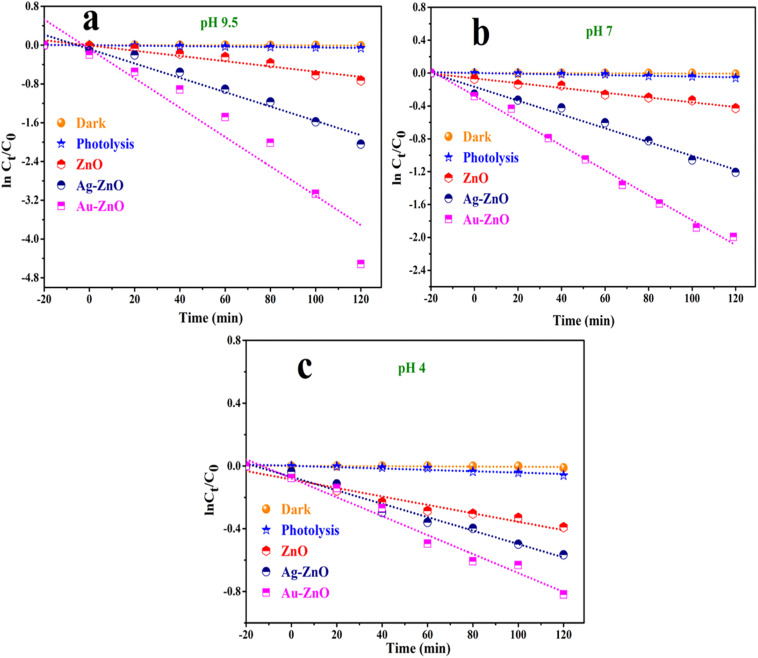
(a–c) Photodegradation kinetics of TC at pH 9.5, 7.0, and 4.0 using ZnO, Ag-ZnO, and Au-ZnO, showing ln(*C*_*t*_/*C*_0_) *vs.* time plots confirming pseudo-first-order kinetics [catalyst: 20 mg, TC: 2 × 10^−3^ M, time: 120 min. at room temperature].

Comparative degradation studies conducted under different pH conditions (Fig. 11a–c) demonstrated that both Au-ZnO and Ag-ZnO NCs significantly outperformed pristine ZnO, confirming the beneficial impact of noble metal decoration. The temporal concentration profiles (*C*_*t*_/*C*_0_*vs.* time) exhibited exponential decay behavior (Fig. S15a–c), characteristic of pseudo-first-order kinetics under the employed experimental conditions. Kinetic analysis through ln(*C*_*t*_/*C*_0_) *vs.* time plots (Fig. 12) yielded linear relationships, from which apparent rate constants were determined and are summarized in Table S2. The enhanced photocatalytic activity of the metal-decorated samples correlates with improved visible light absorption, enhanced charge separation efficiency, and the synergistic effects of plasmonic enhancement.

To unequivocally confirm the necessity of both the photocatalyst and light, comprehensive control experiments were conducted. Photolysis in the absence of any catalyst yielded a mere 5.78% degradation after 120 minutes, while experiments conducted in the dark without any catalyst resulted in only 1.17% degradation. These results, shown in Fig. 11a–c, definitively establish that the observed degradation is exclusively photocatalytic in nature. The profound influence of environmental parameters was explored by systematically evaluating the effect of solution pH on photocatalytic efficiency. Given that pH directly modulates pollutant ionization, catalyst surface charge, and reactive oxygen species (ROS) generation pathways, degradation experiments were conducted at three representative pH levels: 4.0, 7.0, and 9.5. As compellingly illustrated in Fig. 11a–c, photocatalytic activity consistently increased with alkalinity across all tested catalysts. At pH 9.5, Au-ZnO exhibited the highest photocatalytic activity, achieving an impressive 99% degradation of TC within 120 minutes. Ag-ZnO followed with 87% degradation, significantly outperforming pristine ZnO, which achieved 51% degradation.

Kinetic analysis under these optimized alkaline conditions was further refined using the Langmuir–Hinshelwood (L–H) model, a widely accepted framework for describing heterogeneous photocatalysis involving surface-bound species. At low pollutant concentrations, the L–H model conveniently simplifies to a pseudo-first-order expression:^5^

6 ln(*C*_*t*_/*C*_0_) = −*kt*

where the initial and time-dependent concentrations of TC are indicated by *C*_0_ and *C*_*t*_, respectively, and the apparent rate constant is represented by *k*. Linear plots of ln(*C*_*t*_/*C*_0_) *versus* time (Fig. 12a–c) rigorously confirmed the applicability of this kinetic model across all catalytic systems. The detailed kinetic parameters (intercept, slope, and *R*^2^) for TC degradation at pH 9.5, 7, and 4 and under dark, photolysis, and photocatalytic conditions for ZnO, Ag-ZnO, and Au-ZnO are presented in Table S2, obtained from linear fitting of the ln(*C*_*t*_/*C*_0_) *versus* time plots. The calculated rate constants at pH 9.5 underscore the pronounced enhancement achieved through noble metal decoration: 0.0303 ± 0.0039 min^−1^ for Au-ZnO, 0.01481 ± 0.0012 min^−1^ for Ag-ZnO, and 0.0054 ± 0.0006 min^−1^ for pristine ZnO. These values indicate approximately 6-fold and 3-fold enhancements for Au-ZnO and Ag-ZnO, respectively, over pristine ZnO. These substantial improvements are primarily attributed to several synergistic effects induced by noble metal decoration, including localized surface plasmon resonance (LSPR) effects, significantly improved charge carrier separation, and increased surface reactivity.^19^ A complete set of degradation percentages and kinetic parameters under varying pH conditions is comprehensively provided in Table S3. This observed pH-dependent behavior not only reinforces the critical role of chemistry of reaction medium in dictating photocatalytic efficiency but also establishes a foundational understanding for subsequent investigations into reactive oxygen species formation, charge carrier dynamics, and catalyst stability across diverse environmental regimes.

#### 3.8.2. Parametric optimization for enhanced degradation

Building upon the established photocatalytic behavior, further mechanistic insights were gleaned by systematically evaluating crucial operational parameters that govern catalyst–pollutant interactions. The solution pH proved to be a critical determinant, profoundly influencing photocatalyst surface charge, tetracycline speciation, adsorption capacity, and the generation of reactive oxygen species. Degradation experiments conducted at pH 4.0, 7.0, and 9.5 (Fig. 11) consistently showed photocatalytic performance improving with increasing alkalinity. This enhancement was particularly pronounced for Au-ZnO, which achieved 99% TC removal at pH 9.5 compared to 55.93% at pH 4.0, while Ag-ZnO demonstrated 87% degradation efficiency at pH 9.5 *versus* 43.20% at pH 4.0. In contrast, pristine ZnO exhibited substantially lower photocatalytic activity under all pH conditions.

**Fig. 13:**
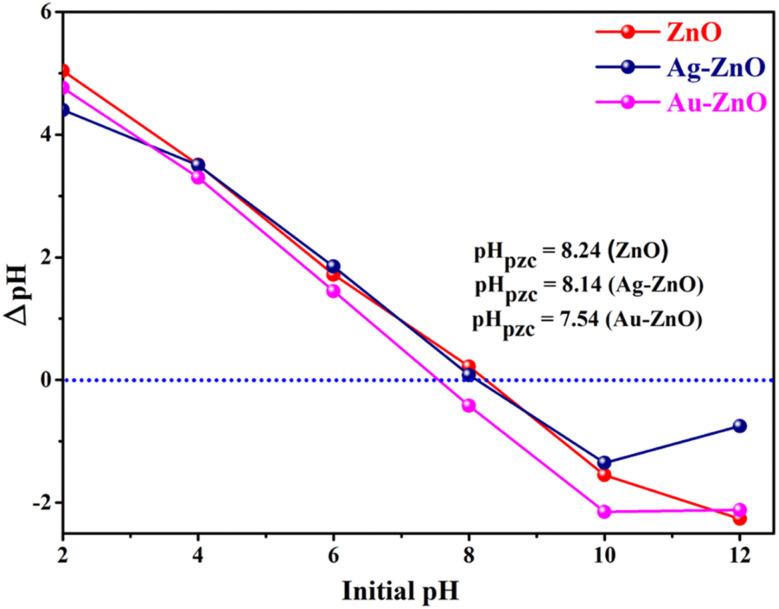
Determination of point of zero charge (pH_pzc_) for ZnO and Ag-ZnO, and Au-ZnO nanocomposites.

This pH-dependent behavior is closely linked to the surface charge characteristics of the photocatalysts, as inferred from their point of zero charge (pH_pzc_) values: 8.24 for ZnO, 8.14 for Ag-ZnO, and 7.54 for Au-ZnO (Fig. 13).^34^ At pH values above their respective pH_pzc_, the catalyst surfaces acquire a negative charge, facilitating favorable electrostatic interactions with anionic tetracycline species, which predominate above pH 7.7 based on p*K*_a_ values of TC (3.3, 7.7, and 9.7).^34,51,52^ This enhanced electrostatic attraction promotes stronger surface adsorption of TC, thereby improving charge transfer efficiency and boosting ROS-mediated degradation pathways. In contrast, under acidic conditions, both the catalyst surface and TC species are positively charged or neutral, leading to electrostatic repulsion and reduced adsorption.^34^ Moreover, proton competition at low pH inhibits access to active sites, further limiting photocatalytic activity. Collectively, these effects underscore the critical role of pH in optimizing photocatalytic degradation through modulation of surface chemistry and interfacial interactions.^15^

Catalyst loading and pollutant concentration were identified as key factors influencing photocatalytic performance. An initial increase in the catalyst dosage enhanced degradation efficiency by providing more active sites for the reaction; however, beyond 20 mg, the efficiency declined due to particle agglomeration, which reduced the effective surface area, and increased solution turbidity, which limited light penetration to the catalyst surface. To further assess the sensitivity of our catalysts, photocatalytic degradation of TC was evaluated at an elevated concentration of 3 × 10^−3^ M (1.5-fold increase) at a fixed catalyst dosage of 20 mg. Comparative evaluation showed that pure ZnO degraded only 17.21% of the pollutant, whereas Ag-ZnO and Au-ZnO achieved higher degradation efficiencies of 30.31% and 35.42%, respectively, after 120 minutes of visible-light irradiation (Fig. S16). These values are markedly lower than those obtained under optimized conditions, clearly demonstrating the negative impact of higher pollutant concentration on degradation performance.

The reduced efficiency at elevated tetracycline levels can be attributed to multiple factors: (i) saturation of the catalyst's active sites, (ii) shielding of the catalyst surface from light by excess pollutant molecules, and (iii) quenching of reactive oxygen species (ROS), including hydroxyl (˙OH) and superoxide radicals (˙O_2_^−^), by the surplus tetracycline molecules. These findings unequivocally demonstrate that alkaline pH, moderate catalyst dosage, and low-to-moderate initial TC concentrations are paramount for achieving optimal visible-light degradation in real-world applications.^34,52^

#### 3.8.3. Mass spectrometric analysis and the proposed degradation pathway of TC

**Fig. 14:**
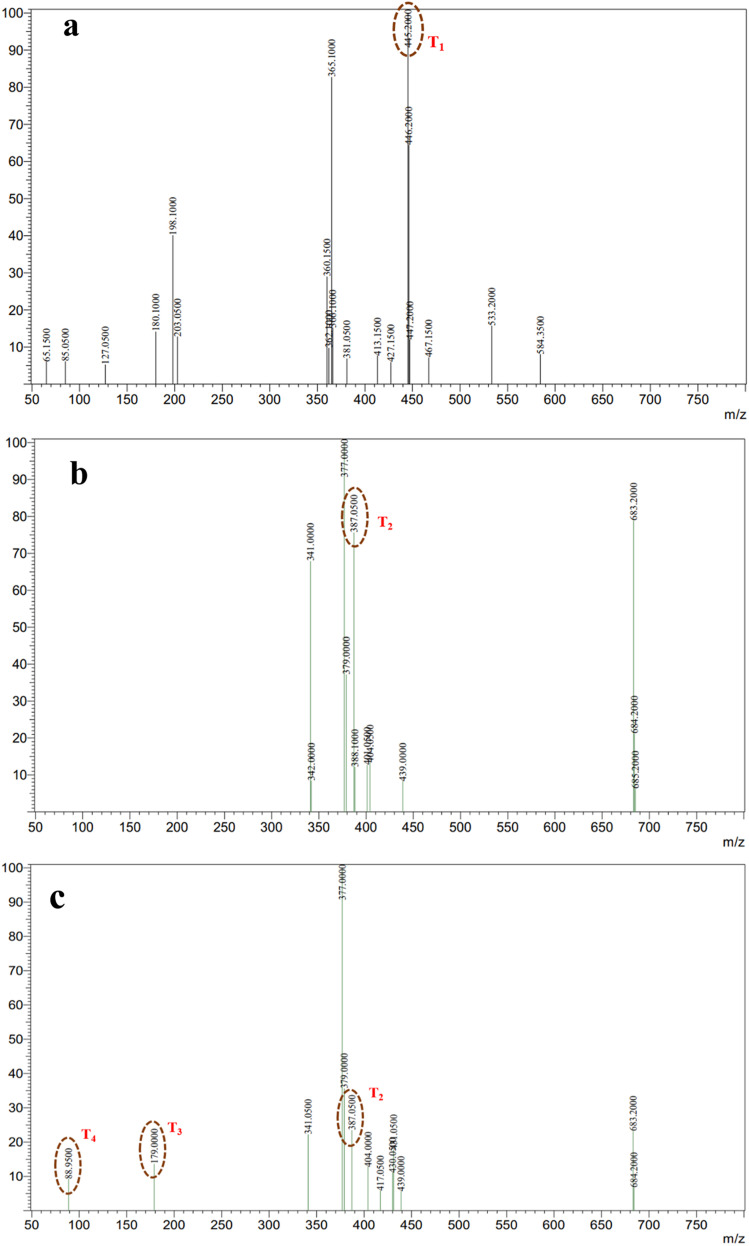
Mass spectra of tetracycline during the degradation process: (a) blank TC solution, (b) after 60 min, and (c) after 120 min of photocatalytic treatment using Ag-ZnO NCs.

The photocatalytic degradation mechanism of tetracycline over Ag-ZnO was elucidated through electrospray ionization mass spectrometry (ESI-MS), with representative mass spectra presented in Fig. 14. The mass spectrum of the blank TC solution (Fig. 14a) displays the characteristic molecular ion peak at *m*/*z* = 445.20 (T_1_), confirming the intact parent compound structure. Following 60 min of photocatalytic irradiation under visible light (Fig. 14b), the parent ion intensity decreased substantially, concurrent with the emergence of new fragment ions at *m*/*z* = 387.05 (T_2_). These intermediates indicate initial demethylation, amide removal, and side-chain cleavage reactions initiated by radical species and photogenerated holes on the Ag-ZnO surface.

**Scheme 2:**
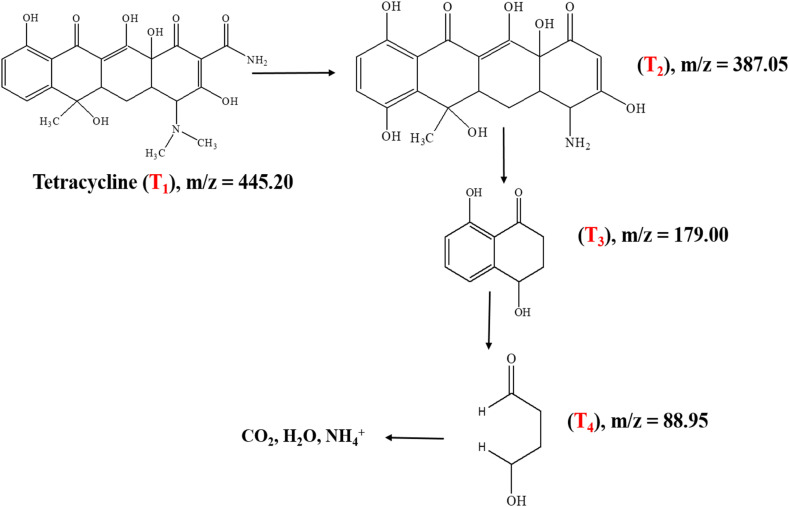
Proposed photocatalytic degradation pathway of tetracycline over Ag-ZnO nanocomposites, constructed based on the intermediate fragments identified from mass spectrometric analysis.

Continued irradiation for 120 min (Fig. 14c) resulted in the appearance of additional low *m*/*z* fragments including *m*/*z* = 179 (T_3_) and *m*/*z* = 88.98 (T_4_), reflecting extensive ring-opening reactions and progressive oxidative breakdown of the tetracycline aromatic structure.^53^ The sequential appearance and subsequent disappearance of these intermediates with increasing irradiation time indicate stepwise degradation proceeding through multiple fragmentation pathways. The proposed degradation mechanism, illustrated in Scheme 2, comprises three distinct stages: (1) initial N-demethylation and functional group removal generating T_2_ and T_3_ intermediates, (2) aromatic ring cleavage and partial oxidation producing smaller fragments, T_4_, and (3) complete mineralization to CO_2_, H_2_O, and inorganic nitrogen species through continued ROS attack. The progressive reduction in the parent ion intensity coupled with the systematic formation of successively smaller fragments collectively demonstrates efficient and complete photocatalytic mineralization of tetracycline under visible-light irradiation using the Ag-ZnO catalyst. The mass-spectrometric results and the proposed degradation pathway are in good agreement with the TC degradation mechanism reported by E. Vijayakumar *et al.*^53^

#### 3.8.4. Methylene blue dye degradation: efficiency and kinetics

**Fig. 15:**
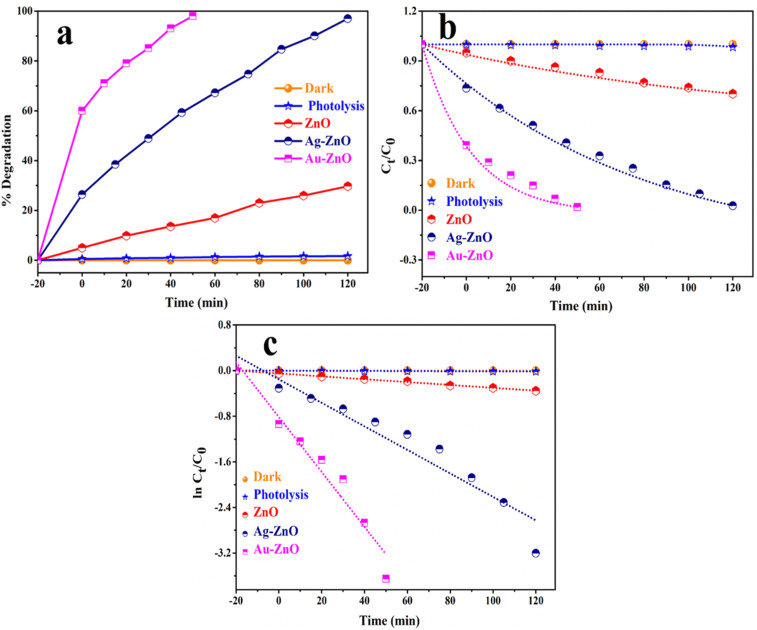
Degradation of MB in the presence of ZnO NSs, Au-ZnO NCs and Ag-ZnO NCs (a) % degradation, (b) *C*_*t*_/*C*_0_*vs.* time (minute) and (c) ln(*C*_*t*_/*C*_0_) *vs.* time (minute) graph under visible light [catalyst: 50 mg, MB: 1 × 10^−5^ M, time: 120 min. at room temperature].

The photocatalytic activities of pristine ZnO, Ag-ZnO and Au-ZnO were also rigorously evaluated for the photodegradation of MB dye (1.0 × 10^−5^ M) under visible light irradiation. MB concentration was monitored by measuring its characteristic absorbance at *λ*_max_ = 660 nm using UV-vis spectroscopy.^40^ Comparative percent degradation and kinetic profiles are depicted in Fig. 15a–c, with detailed quantitative results summarized in Table S4.

To confirm the photostability of MB and the necessity of the catalyst, control experiments were performed. Photolysis under visible light without any catalyst resulted in only 1.68% degradation after 120 minutes, confirming negligible auto-degradation and excellent dye photostability. Adsorption studies performed by stirring the MB solution with each catalyst in the dark for 30 minutes revealed notable differences: Au-ZnO adsorbed ∼60% of the dye, Ag-ZnO ∼26%, and pristine ZnO only ∼5% (Fig. 15a). The observed decrease in the absorbance intensity after dark equilibration is attributed to dye adsorption rather than photodegradation, as no illumination was provided to activate photocatalytic processes. Time-dependent UV-visible absorption spectra during degradation are provided in the SI (Fig. S17a–c).

Upon visible light illumination, a significant enhancement in degradation performance was recorded. As shown in Fig. 15a, Au-ZnO demonstrated the fastest and most efficient photocatalytic response, achieving 98% degradation within just 50 minutes. Ag-ZnO achieved ∼97% degradation over 120 minutes, while pristine ZnO showed only 29.72% degradation under identical conditions. These results align with corresponding *C*_*t*_/*C*_0_*versus* time plots (Fig. 15b), which exhibited an exponential decay in MB concentration, consistent with kinetic modeling. The degradation kinetics adhered to a pseudo-first-order model, as evidenced by the linear relationship in the ln(*C*_*t*_/*C*_0_) *versus* time plots shown in Fig. 15c. The corresponding kinetic parameters, obtained from linear fits of the ln(*C*_*t*_/*C*_0_) *versus* time plots, are summarized in Table S5. The apparent rate constants (*k*) were determined to be 0.00251 ± 0.00006 min^−1^ for pristine ZnO, 0.0206 ± 0.0020 min^−1^ for Ag-ZnO, and 0.0518 ± 0.0048 min^−1^ for Au-ZnO. These values indicate a substantial enhancement in photocatalytic activity upon noble metal decoration, with Au-ZnO exhibiting a ⁓20-fold increase and Ag-ZnO a ⁓9-fold increase in the rate constant compared to bare ZnO. For the Au-ZnO sample, the apparent rate constant calculated after the illumination stage (excluding the dark adsorption period) is 5.2 × 10^−2^ min^−1^, whereas the combined rate constant (dark + light) is 5.175 × 10^−2^ min^−1^. The negligible difference between these two values confirms that the dark adsorption stage contributes minimally to the overall kinetics, and that the photocatalytic rate constant primarily reflects the illumination-driven process. The experimental data exhibited excellent conformity to pseudo-first-order kinetics, as presented in Table S4.

To evaluate the concentration-dependent activity, experiments were conducted at an increased MB dye concentration of 2 × 10^−5^ M (two-fold higher than our optimized concentration) while keeping the catalyst loading constant (Fig. S18). A significant decrease in degradation efficiency was observed, with pure ZnO achieving only 10% degradation and Ag-ZnO reaching 33% under identical conditions. This reduction can be attributed to surface site saturation, competitive adsorption among dye molecules, and light-screening effects. Notably, these findings are consistent with the results obtained for TC degradation, where a similar decline in efficiency was also observed at elevated pollutant concentrations. Overall, the outcomes clearly demonstrate the sensitivity of the catalytic system towards pollutant concentration and align well with the Langmuir–Hinshelwood model, wherein reaction rates are restricted at higher substrate levels. The observed trend in the degradation efficiency and rate (Au-ZnO > Ag-ZnO > ZnO) clearly demonstrates the crucial role of noble metals in boosting photocatalytic performance under visible light.

#### 3.8.5. Mass spectrometric analysis and the proposed degradation pathway of methylene blue

**Fig. 16:**
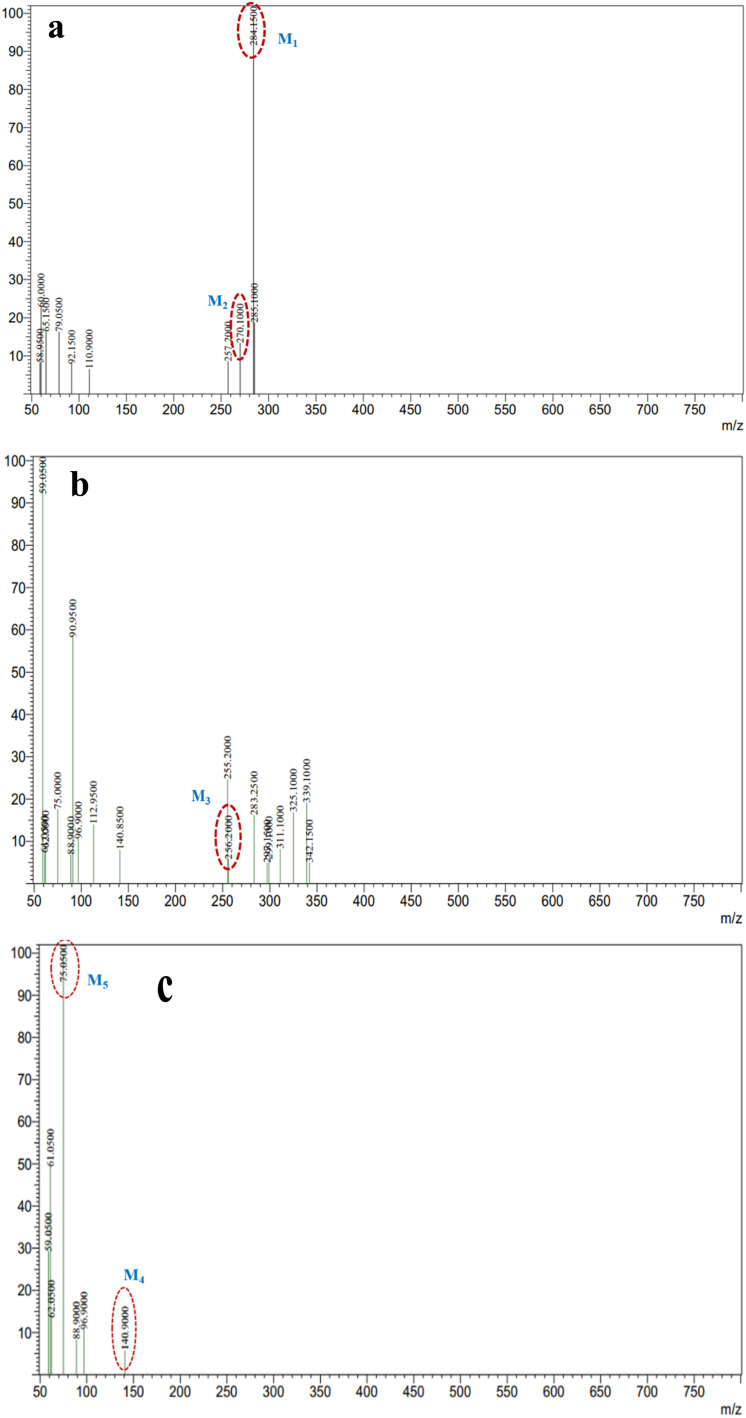
Mass spectra of methylene blue during the photocatalytic degradation process: (a) initial mixture, 0 min. (b) After 60 min, and (c) after 120 min of photocatalytic treatment using Ag-ZnO NCs.

The photocatalytic degradation mechanism of methylene blue over Ag-ZnO NCs was elucidated through ESI-MS, with representative mass spectra presented in Fig. 16. The mass spectrum of the initial MB solution (Fig. 16a) displays the characteristic molecular ion peak at *m*/*z* = 284.15 (M_1_), along with a secondary fragment at *m*/*z* = 270.10 (Azure B, M_2_), confirming the intact parent dye structure. Following 60 min of visible-light irradiation (Fig. 16b), the parent ion intensity decreased substantially with the concurrent emergence of a new intermediate at *m*/*z* = 256.20 (Azure A, M_3_), indicative of sequential N-demethylation and partial disruption of the aromatic chromophoric system through hydroxyl radical-mediated oxidation.

**Scheme 3:**
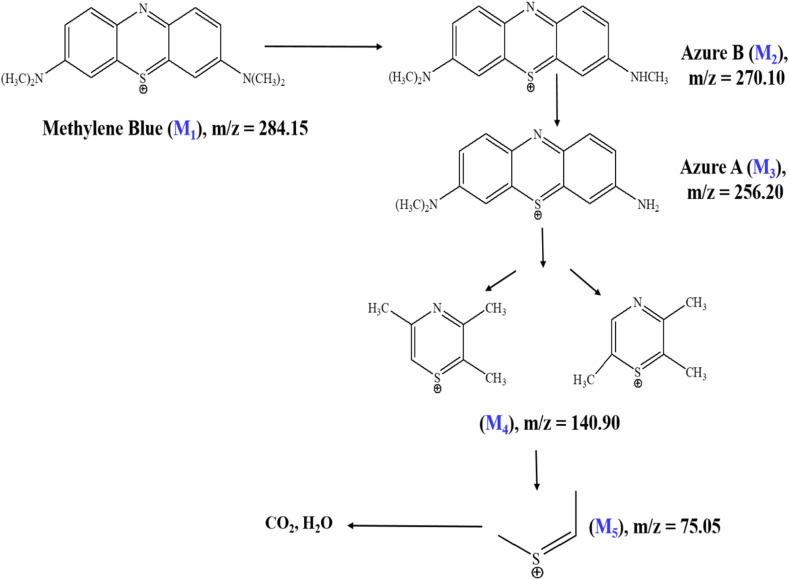
Proposed photocatalytic degradation pathway of methylene blue over Ag-ZnO nanocomposites, constructed based on the intermediate fragments identified from mass spectrometric analysis.

Extended irradiation for 120 min (Fig. 16c) resulted in the appearance of additional low *m*/*z* fragments at *m*/*z* = 140.90 (M_4_) and *m*/*z* = 75.05 (M_5_), reflecting successive aromatic ring-opening reactions and further oxidative cleavage.^54^ The systematic progression from the parent ion to progressively smaller mass fragments indicates stepwise photocatalytic degradation proceeding through distinct transformation stages. These spectral observations support the proposed degradation mechanism illustrated in Scheme 3, which comprises three sequential phases: (1) N-demethylation and partial oxidation of the chromophoric core generating M_3_, (2) aromatic ring-opening and C–N bond cleavage producing M_4_ and M_5_ intermediates, and (3) complete mineralization to CO_2_, H_2_O, and inorganic nitrogen products. The marked reduction in the parent ion intensity coupled with the sequential formation of smaller mass products collectively demonstrates efficient photocatalytic degradation and mineralization of methylene blue under visible-light irradiation using the Ag-ZnO catalyst.^54^

#### 3.8.6. Co-degradation of tetracycline hydrochloride (TC) and methylene blue (MB)

After optimizing the photocatalytic degradation conditions for the individual pollutants, TC and MB, co-degradation experiments were conducted to evaluate the simultaneous removal efficiency of both contaminants under identical conditions. The optimized parameters were used for both pollutants in the combined system: a TC concentration of 2.0 × 10^−3^ M (962 mg L^−1^), MB concentration of 1.0 × 10^−5^ M (3.2 mg L^−1^), total solution volume of 50 mL, catalyst dose of 20 mg, irradiation time of 120 minutes, and pH 7.0. The pH value of 7.0 was selected based on the results of individual degradation experiments. A lower catalyst loading of 20 mg was preferred because higher catalyst concentrations were found to reduce the degradation efficiency, likely due to light scattering and catalyst agglomeration, which decrease the effective active surface area.

**Fig. 17:**
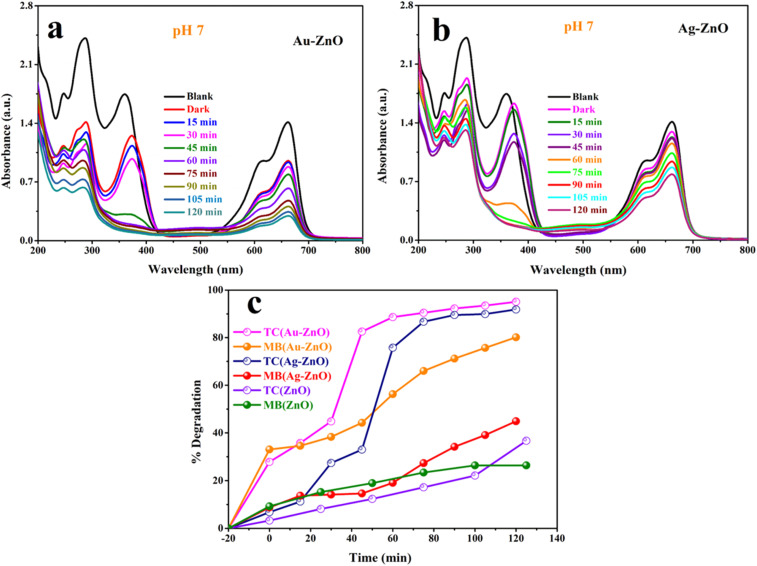
Co-degradation of TC and MB under visible-light irradiation using (a) Au-ZnO and (b) Ag-ZnO nanocomposites, with (c) comparative percentage degradation for Au-ZnO, Ag-ZnO, and ZnO after 120 min [catalyst dose: 20 mg; pH 7].

Under the optimized reaction conditions, both Au-ZnO and Ag-ZnO NCs exhibited excellent photocatalytic activity toward the simultaneous degradation of TC and MB. As shown in Fig. 17, the UV-vis absorption profiles display a gradual decrease in peak intensities over 120 min of visible-light irradiation for Au-ZnO (Fig. 17a) and Ag-ZnO (Fig. 17b), confirming efficient co-degradation of both pollutants. Fig. 17c presents the corresponding percentage degradation of TC (monitored at 358 nm) and MB (monitored at 660 nm), clearly demonstrating the superior performance of the metal-decorated ZnO catalysts in mixed-pollutant systems.^20,40^

The Au-ZnO nanocomposite exhibited the highest photocatalytic performance, achieving 95.04% degradation of TC and 80.00% degradation of MB within 120 min of irradiation. The corresponding pseudo-first-order rate constants were 2.38 × 10^−2^ min^−1^ (TC) and 1.10 × 10^−2^ min^−1^ (MB), as shown in Fig. S19a–c. The Ag-ZnO system exhibited competitive but slightly lower performance, degrading 91.85% of TC and 45.00% of MB under identical conditions, with rate constants of 2.16 × 10^−2^ min^−1^ and 0.40 × 10^−2^ min^−1^. In stark contrast, pristine ZnO displayed substantially diminished activity, achieving only 36.71% TC and 26.40% MB degradation with correspondingly low rate constants of 0.28 × 10^−2^ min^−1^ and 0.20 × 10^−2^ min^−1^. These results underscore the critical role of noble metal decoration in enhancing photocatalytic performance, with Au-ZnO exhibiting approximately 8.5-fold and 5.5-fold improvements in TC and MB degradation rates, respectively, compared to ZnO. The detailed degradation efficiencies and kinetic parameters are summarized in Table S6.

Extended irradiation periods further amplified the performance of Au-ZnO, yielding 98.83% TC and 97.18% MB degradation after 240 min, indicating near-complete mineralization of both pollutants (Fig. S20). In the case of Ag-ZnO, a moderate enhancement was observed upon increasing the catalyst dosage to 50 mg, resulting in 93.67% TC and 50% MB degradation within 120 min. The corresponding UV-vis profiles and kinetic plots (ln *C*_*t*_/*C*_0_) are provided in Fig. S21a and b, respectively.

The co-degradation experiments show that both TC and MB can be simultaneously degraded under visible light using Ag-ZnO and Au-ZnO photocatalysts. Both nanocomposites retain high activity even under mixed-pollutant conditions, demonstrating strong tolerance to competing substrates. This efficient multi-pollutant degradation highlights their superior photocatalytic performance and clear potential for real wastewater treatment containing diverse pharmaceutical and dye contaminants.

The simultaneous photocatalytic degradation of MB and TC over Au-ZnO was analyzed by ESI-MS. The initial solution (Fig. S22a) displays parent ion peaks at *m*/*z* = 284.1, 270.1 (M_1_ and M_2_ for MB) and *m*/*z* = 445.2 (T_1_ for TC). After 60 min of visible-light irradiation (Fig. S22b), both parent peaks decreased significantly with concurrent emergence of intermediates *m*/*z* = 256.15 (M_3_) and *m*/*z* = 387.05, 179 (T_2_ and T_3_), indicating simultaneous degradation of both pollutants. Extended irradiation for 120 min (Fig. S22c) resulted in near-complete disappearance of parent ions and formation of additional low-mass fragments (*m*/*z* = 179, 88.95 corresponding to T_3_ and T_4_) from both degradation pathways. These observations confirm efficient co-mineralization of MB and TC through photogenerated reactive oxygen species, demonstrating that Au-ZnO effectively degrades both pollutants simultaneously, establishing its viability for practical multi-pollutant wastewater treatment.

#### 3.8.7. Photocatalytic mechanism and reactions

**Scheme 4:**
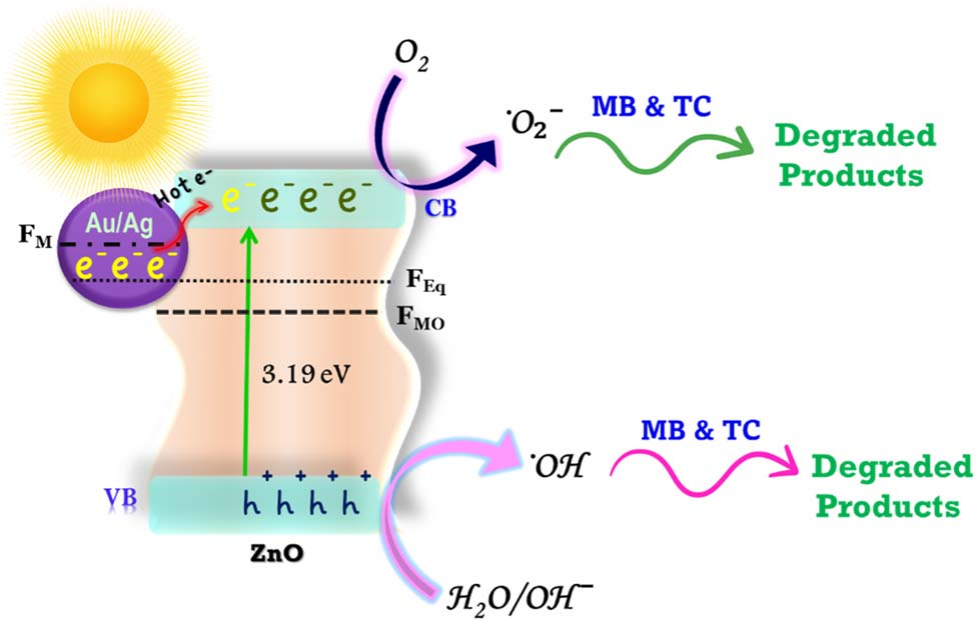
Schematic illustration of the photocatalytic mechanism for MB dye and TC antibiotic degradation over the Au-ZnO and Ag-ZnO NCs under visible light irradiation.

The photocatalytic degradation of TC and MB over Au- and Ag-decorated ZnO NSs under visible light irradiation proceeds through a plasmon-mediated charge transfer mechanism, as schematically depicted in Scheme 4.

Pristine ZnO, characterized by a wide band gap of approximately 3.19 eV (as determined by UV-vis absorption), exhibits intrinsic photocatalytic activity predominantly under ultraviolet irradiation due to the limited overlap of the solar spectrum with its absorption edge.^5^ The Fermi level of ZnO (F_MO_) is intrinsically lower than that of metallic Au or Ag nanoparticles (F_M_).^22^ Upon establishing intimate interfacial contact between ZnO and these noble metals, electrons migrate from the higher F_M_ (metal) to the lower F_MO_ (ZnO) until the Fermi levels equilibrate at a common value (F_Eq_).^19^ This electron transfer results in downward band bending within ZnO near the interface, forming an accumulation-type Schottky junction that plays a decisive role in modulating charge-carrier dynamics.^16,40,52^ The Schottky barrier height is governed by the work function difference between ZnO (*χ* ≈ 5.4 eV) and the respective metals [Au: *χ* ≈ 5.0–5.3 eV; Ag: *χ* ≈ 4.3 eV],^19,55^ with Au producing a more favorable band offset and stronger accumulation field compared to Ag.^22^

Under visible-light irradiation, the noble metal (Au/Ag) nanoparticles exhibit localized surface plasmon resonance (LSPR), a collective coherent oscillation of conduction electrons at the characteristic plasmon frequency.^56,57^ This plasmonic excitation is particularly pronounced for Au and Ag due to their favorable d-band electronic structures and size-dependent optical properties, which enable efficient light absorption across the visible spectrum and generation of energetic hot carriers. The LSPR process generates a non-equilibrium population of hot electrons with energies significantly above the Fermi level (*E*_F_), exceeding those produced by direct band-gap excitation of ZnO alone.^16,24,31^ A substantial fraction of these energetic hot electrons possesses sufficient kinetic energy to surmount the Schottky barrier formed at the Au/Ag-ZnO interface and undergo direct injection into the conduction band (CB) of ZnO, thereby circumventing the low carrier mobility limitations of pristine ZnO. Concurrently, the corresponding holes generated in the noble metal remain localized on its surface, establishing a long-lived spatial separation of charges with electrons in the ZnO CB and holes in the noble metal, which strongly suppresses electron–hole recombination.^41,58^ Furthermore, the high concentration of accumulated electrons in the ZnO CB and the localized hole population on the noble metal surface collectively facilitate the generation of reactive oxygen species (ROS), particularly superoxide radicals (˙O_2_^−^) and hydroxyl radicals (˙OH), through sequential reduction–oxidation reactions.

The injected electrons in the ZnO CB reduce surface-adsorbed O_2_ to generate superoxide radical anions (˙O_2_^−^):^20,58^

7 e^−^(CB) + O_2_ → ˙O_2_^−^

Simultaneously, photogenerated holes (h^+^) on the noble metal oxidize water molecules or hydroxide ions to produce hydroxyl radicals (˙OH):^19^

8 h^+^ + H_2_O → ˙OH + H^+^

9 h^+^ + OH^−^ → ˙OH

Additionally, the generated ˙O_2_^−^ radicals can further react with water to yield more ˙OH radicals:^59^

10 ˙O_2_^−^ + H_2_O → ˙OH + OH^−^ + O_2_

These ROS, particularly ˙O_2_^−^ and ˙OH, possess strong oxidative potential and initiate a cascade of non-selective degradation reactions that effectively break down tetracycline and methylene blue into CO_2_, H_2_O, and inorganic ions under visible light:^28,52^

11 ˙O_2_^−^/˙OH + dye and TC → CO_2_ + H_2_O + inorganic fragments

Comparative photocatalytic studies reveal that Au-ZnO exhibits the highest degradation rate constants for both pollutants, followed by Ag-ZnO, with pristine ZnO showing minimal activity. This performance hierarchy directly reflects the efficiency of hot electron injection and charge retention, which is maximized in Au-ZnO due to its superior F_M_–F_MO_ alignment and enhanced Schottky barrier-mediated suppression of back-electron transfer compared to Ag-ZnO.^22^ To validate the proposed mechanism and determine the primary active species responsible for the enhanced degradation, targeted scavenger experiments were conducted.

#### 3.8.8. Role of active species and the reusability study

To elucidate the photocatalytic degradation mechanism by Ag-ZnO NCs under visible light, the roles of various reactive species were systematically investigated. Trapping experiments were conducted to ascertain the contribution of photogenerated holes (h^+^), electrons (e^−^), hydroxyl radicals (˙OH), and superoxide radicals (˙O_2_^−^). This was achieved by introducing specific scavengers—ethylenediaminetetraacetic acid disodium salt (2Na-EDTA) for h^+^, silver nitrate (AgNO_3_) for e^−^, methanol (CH_3_OH) for ˙OH, and ascorbic acid (C_6_H_8_O_6_) for ˙O_2_^−^.

**Fig. 18:**
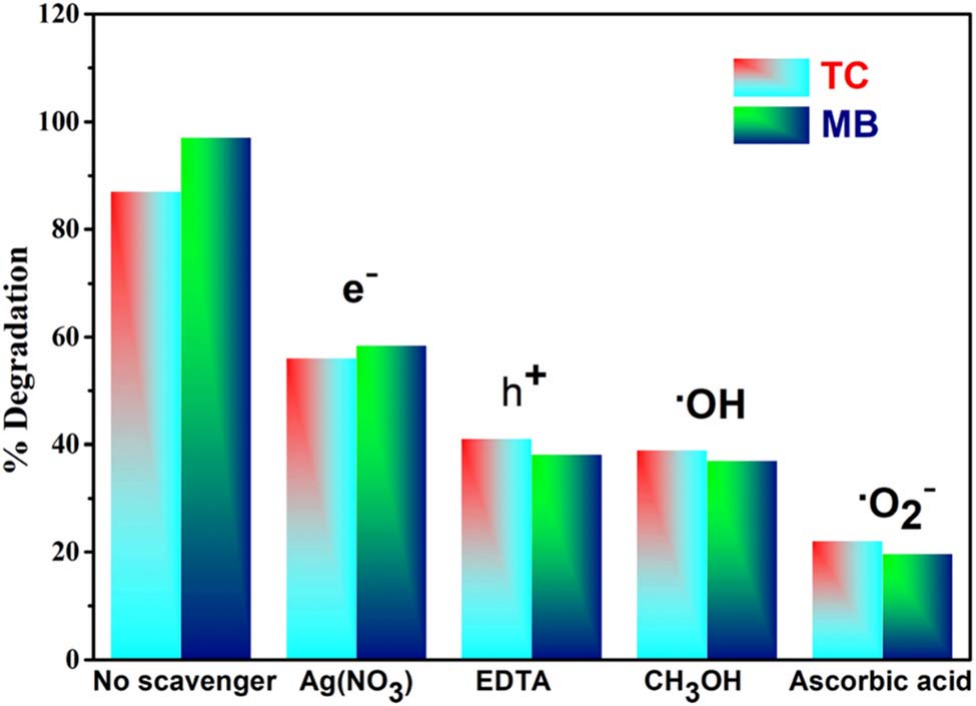
Effect of ascorbic acid, CH_3_OH, 2Na-EDTA, and AgNO_3_ as active scavengers on the degradation of MB dye and TC by using Ag-ZnO NCs [catalyst: 20 mg for TC: 2 × 10^−3^ M, 50 mg for MB: 10^−5^ M, time: 120 min. at room temperature].

The results of the scavenger experiments are depicted in Fig. 18. A negligible reduction in photocatalytic activity was observed upon the addition of AgNO_3_, suggesting a minor role for photogenerated electrons in the direct degradation pathway. The presence of 2Na-EDTA and CH_3_OH induced a moderate suppression of MB degradation, indicating that photogenerated holes (h^+^) and hydroxyl radicals (˙OH) are secondary contributors to the overall process. Most notably, the introduction of ascorbic acid severely inhibited the photocatalytic reaction. The pronounced quenching effect unequivocally indicates that superoxide radicals serve as the dominant oxidative species in the degradation of MB. The established reactivity sequence (˙O_2_^−^ ≫ ˙OH > h^+^ > e^−^) further confirms that the photocatalytic activity of the metal-decorated ZnO NCs is primarily governed by superoxide radicals, as evidenced in Table S7. Scavenging studies by Iqbal *et al.*^60^ and Zhao *et al.*^41^ on MB and reactive black GR degradation, respectively, consistently show ˙O_2_^−^ as the primary oxidative species under visible light, with h^+^ and ˙OH playing secondary roles. The scavenger experiment for TC was conducted using all the specific quenchers, and the results shown in Fig. 18 similarly indicated that superoxide radicals (​˙O_2_^−^) are the primary reactive species responsible for the degradation of TC.^52^

**Fig. 19:**
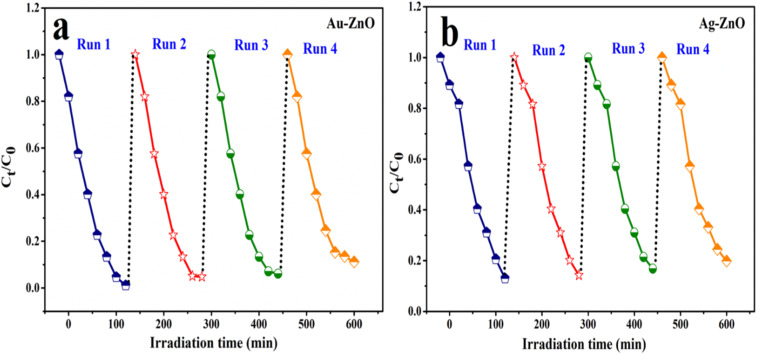
Reusability of the photocatalysts (a) Au-ZnO and (b) Ag-ZnO in degrading TC under visible light over four consecutive cycles.

Beyond elucidating the mechanistic pathway, evaluating the long-term stability and reusability of photocatalysts represents a critical parameter for assessing their practical applicability. The operational durability of metal-decorated ZnO NCs was systematically evaluated through four consecutive photocatalytic cycles for both TC (Fig. 18 and 19a, b) and MB degradation (Fig. S23a and b). For TC degradation, Au-ZnO NCs demonstrated initial degradation efficiencies of 99%, which gradually decreased to 95%, 93%, and 88% over subsequent cycles. Similarly, Ag-ZnO NCs exhibited degradation efficiencies of 87%, 85%, 83%, and 80% over the four cycles, respectively. In MB degradation studies, Ag-ZnO photocatalysts displayed remarkable stability, with degradation efficiency declining minimally from 97% in the first cycle to 87% after four cycles. Au-ZnO NCs also exhibited excellent reusability, retaining 78% degradation efficiency after four cycles compared to the initial 98% performance. The minor loss in activity can be attributed to partial surface fouling, blockage of active sites, and small changes in surface defect states during repeated use. Additionally, the relatively high concentration of TC may further contribute to the observed decline in degradation efficiency over multiple cycles.

Structural analysis of the recycled photocatalysts was carried out to assess their long-term morphological stability and resistance to photocorrosion. HRTEM images recorded after four consecutive photocatalytic cycles (Fig. S24a and b for TC degradation and Fig. S25a and b for MB degradation) show that both Au-ZnO and Ag-ZnO NCs retained their original morphology. The noble metal nanoparticles also remained uniformly dispersed across the ZnO matrix, and no signs of aggregation, lattice distortion, or surface etching were observed, even after repeated exposure to visible light and reactive species. This preserved nanostructure directly supports the excellent reusability and sustained photocatalytic activity of the catalysts.

Post-catalysis XPS analysis further confirms the chemical and electronic stability of the nanocomposites. As shown in Fig. S26 and S27, moderate shifts in the Zn 2p, O 1s, Ag 3d, and Au 4f binding energies were observed upon reuse. For Au-ZnO, the Zn 2p_3/2_ peak shifted from 1021.74 to 1022.44 eV, and the O 1s defect component from 531.31 to 532.29 eV, indicating increased oxygen-vacancy concentration and enhanced metal–support interactions; the Au 4f_7/2_ peak shift (83.54 → 84.17 eV) further supports electron transfer from Au to ZnO. Ag-ZnO exhibited similar trends, with the Zn 2p_3/2_ peak shifting from 1021.30 to 1022.43 eV and the O 1s defect component from 531.34 to 532.67 eV, confirming surface reconstruction and defect formation during photocatalysis. The comparative binding energies summarized in Table S8 verify that both catalysts retain their chemical identity while undergoing beneficial electronic reorganization.

The elemental stability of the noble-metal loading was examined using ICP-MS. Fresh Au-ZnO and Ag-ZnO samples contained 2.04 wt% Au and 1.99 wt% Ag, respectively, confirming successful incorporation of the metals. Post-catalysis ICP-MS analysis of the reused samples showed metal contents of 2.03 wt% Au and 1.98 wt% Ag after four cycles, indicating negligible leaching and demonstrating excellent retention of the noble-metal loading during repeated photocatalytic operation.

A comparative assessment (Table 3) against previously reported noble metal-supported oxide photocatalysts further highlights the superior photocatalytic efficiency of the synthesized systems for degrading MB and TC under visible light. Collectively, these findings establish Au-ZnO and Ag-ZnO as promising, recyclable, and scalable materials for practical environmental remediation applications.

**Table 3:** Comparison of the photocatalytic performance of Au-ZnO and Ag-ZnO nanocomposites with previously reported catalysts for TC and dye degradation

Catalyst	Type of irradiation	Pollutants	% Degradation efficiency	Completion time	Ref.
Ag/ZnO	400 W lamp visible light	MB	∼99%	4 h	19
Au/ZnO	Metal halide lamp (500 W) visible light	MB	60%	2 h	54
Ag-ZnO	Two horizontally fitted 8 W ‘black light’ UV radiation	MB	45%	160 min	61
Ag-TiO_2_	Visible light (500 W)	MB	79.8%	150	62
10%Ag-TiO_2_	200 W tungsten filament bulb, visible light	MB	82.84%	240 min	59
		MO	81.24%	180 min	
3 wt% Ni:ZnO	9W UV lamp	MB	94%	240 min	26
		TC	78%		
0.3-Au-TiO_2_/PVDF	Xenon lamp 300 W	TC	75%	120 min	20
Au-TiO_2_	300 W Xenon lamp visible light	TC	73%	120 min	24
Au-ZnO	12 W white light-emitting diode (LED) lamp source	TC	55%	90 min	21
2.5 weight % Ag/ZnO	24 W visible white LED light	TC	48%	120	34
**Au-ZnO**	**100 W tungsten filament bulb, visible light**	**MB**	**98%**	**50 min**	**Present work**
		**TC**	**99%**	**120 min**	
**Ag-ZnO**		**MB**	**97%**	**120 min**	
		**TC**	**87%**	**120 min**	

## 4. Conclusion

Ag- and Au-decorated ZnO nanostructures were synthesized through a facile wet-chemical route and thoroughly characterized to elucidate their structural features. Their photocatalytic performance was evaluated for the degradation of TC, MB, and their mixed system under visible-light irradiation. Mass spectrometric analysis confirmed the degradation pathways and the formation of key intermediates for both pollutants. This study demonstrates that noble-metal-modified ZnO photocatalysts can efficiently degrade TC and MB simultaneously, with Au-ZnO showing superior activity due to its stronger plasmonic response and more effective charge separation. Both Ag-ZnO and Au-ZnO retain structural stability and catalytic performance over multiple cycles, highlighting their durability and practical applicability. The tetracycline concentration used in this study (962 mg L^−1^) represents high-strength pharmaceutical wastewater rather than typical environmental levels; therefore, the findings are most relevant to pharmaceutical and hospital effluent treatment. Overall, these results underscore the promise of noble-metal-ZnO nanostructures as robust, solar-responsive photocatalysts for addressing complex, multi-component wastewater systems and provide a useful framework for designing next-generation materials for environmental remediation.

## Author contributions

A. K. performed the synthesis, characterization, formal analysis, and investigation of the photocatalytic systems and wrote the original draft of the manuscript. S. C. assisted with data analysis and reaction monitoring. M. D. S. and R. K. M. contributed to manuscript review, editing and corrections. P. K. contributed to the characterization work. P. F. supervised the research work and reviewed and edited the manuscript.

## Conflicts of interest

The authors declare that they have no known financial or personal conflicts of interest that could have influenced the work presented in this paper.

## Supplementary Material

NA-008-D5NA00878F-s001

## Data Availability

Additional data will be made available from the corresponding author upon request. All data supporting this study are included in the article and supplementary information (SI). Supplementary information is available. See DOI: https://doi.org/10.1039/d5na00878f.

## References

[cit1] Bisht K., Kumar G., Dutta R. K. (2022). Ind. Eng. Chem. Res..

[cit2] Min F., Wei Z., Yu Z., Xiao Y., Guo S., Song R., Li J. (2022). Dalton Trans..

[cit3] Lin J., Ye W., Xie M., Seo D. H., Luo J., Wan Y., Van der Bruggen B. (2023). Nat. Rev. Earth Environ..

[cit4] Yu J., Zhang C., Yang Y., Su T., Yi G., Zhang X. (2023). Phys. Chem. Chem. Phys..

[cit5] Jadoun S. (2024). ACS ES&T Water.

[cit6] Wang C., Lin C. Y., Liao G. Y. (2020). J. Water Process Eng..

[cit7] Liu T., Zhao W., Meng S., Dong B., Shi N., Shi W. (2025). J. Mater. Chem. C.

[cit8] Srivastava S. K. (2024). RSC Appl. Interfaces.

[cit9] Benmammar F., Ayadi A., Maifi L., Chari A., Agroui K. (2024). J. Sol-Gel Sci. Technol..

[cit10] Mohammed W., Matalkeh M., Al Soubaihi R. M., Elzatahry A., Saoud K. M. (2023). ACS Omega.

[cit11] Tichapondwa S. M., Newman J. P., Kubheka O. (2020). Phys. Chem. Earth.

[cit12] Mei W., Lu H., Dong R., Tang S. S., Xu J. (2024). ACS Appl. Nano Mater..

[cit13] Taha K. K., Mustafa M. M., Ahmed H. A. M., Talab S. (2019). Z. Naturforsch. A.

[cit14] Yangjeh A. H., Pournemati K. (2024). Crit. Rev. Environ. Sci. Technol..

[cit15] Meena P. L., Surela A. K., Chhachhia L. K., Meena J., Meena R. (2025). Nanoscale Adv..

[cit16] Singh S. (2022). Mater. Today Commun..

[cit17] Kumari P., Pande S., Fageria P. (2023). Environ. Sci. Pollut. Res..

[cit18] Sanakousar M. F., Vidyasagar C. C., Jimenez-Perez V. M., Jayanna B. K., Shridhar A. H., Prakash K. (2021). J. Hazard. Mater. Adv..

[cit19] Ansari S. A., Khan M. M., Ansari M. O., Lee J., Cho M. H. (2013). J. Phys. Chem. C.

[cit20] Yan M., Wu Y., Liu X. (2021). J. Alloys Compd..

[cit21] Ahmed S. A., Hasan M. N., Altass H. M., Bera A., Alsantali R. I., Pan N., Alzahrani A. Y. A., Bagchi D., Al-Fahemi J. H., Khder A. S., Pal S. K. (2022). ACS Appl. Nano Mater..

[cit22] Fageria P., Gangopadhyay S., Pande S. (2014). RSC Adv..

[cit23] Busila M., Musat V., Alexandru P., Romanitan C., Brincoveanu O., Tucureanu V., Mihalache I., Iancu A. V., Dediu V. (2023). Int. J. Mol. Sci..

[cit24] Wang C., Wu Y., Lu J., Zhao J., Cui J., Wu X., Yan Y., Huo P. (2017). ACS Appl. Mater. Interfaces.

[cit25] Agalya S., Krishna Veni K., Nehru L. C., Eswaramoorthy N. (2025). J. Sol-Gel Sci. Technol..

[cit26] Shkir M., Palanivel B., Khan A., Kumar M., Chang J. H., Mani A., AlFaify S. (2022). Chemosphere.

[cit27] Zhou Y., Chen G., Yu Y., Yan C., Sun J., He F. (2016). J. Mater. Chem. A.

[cit28] Stanley R., Jebasingh J. A., Stanley P. K., Ponmani P., Shekinah M. E., Vasanthi J. (2021). Optik.

[cit29] Mostafa A. M., Mwafy E. A. (2020). J. Mater. Res. Technol..

[cit30] Juneja S., Madhavan A. A., Ghosal A., Moulick R. G., Bhattacharya J. (2018). J. Hazard. Mater..

[cit31] Zhu X., Wang J., Yang D., Liu J., He L., Tang M., Feng W., Wu X. (2021). RSC Adv..

[cit32] Senthilkumar N., Ganapathy M., Arulraj A., Meena M., Vimalan M., Potheher I. V. (2018). J. Alloys Compd..

[cit33] Mahala C., Sharma M. D., Basu M. (2020). ACS Appl. Nano Mater..

[cit34] Onkani S. P., Akpotu S. O., Diagboya P. N., Mtunzi F., Osabohien E. (2024). ACS Sustainable Resour. Manage..

[cit35] Boukhoubza I., Khenfouch M., Achehboune M., Leontie L., Galca A. C., Enculescu M., Carlescu A., Guerboub M., Mothudi B. M., Jorio A., Zorkani I. (2020). Nanomaterials.

[cit36] Sahu D., Panda N. R., Acharya B. S., Panda A. K. (2014). Opt. Mater..

[cit37] Zamiri R., Rebelo A., Zamiri G., Adnani A., Kuashal A., Belsley M. S., Ferreira J. M. F. (2014). RSC Adv..

[cit38] Vu A. T., Pham T. A. T., Do X. T., Tran V. A., Le V. D., Truong D. D., Nguyen T. H., Nguyen M. V. (2021). Adsorpt. Sci. Technol..

[cit39] Ye Z., Miao F., Tao B., Zang Y., Chu P. K. (2021). Ionics.

[cit40] Verma S., Rao B. T., Jayabalan J., Rai S. K., Phase D. M., Srivastava A. K., Kaul R. (2019). J. Environ. Chem. Eng..

[cit41] Zhao X., Su S., Wu G., Li C., Qin Z., Lou X., Zhou J. (2017). Appl. Surf. Sci..

[cit42] Gurusamy V., Krishnamoorthy R., Gopal B., Veeraravagan V., Neelamegam P. (2016). Inorg. Nano-Met. Chem..

[cit43] Alzahrani E. A., Nabi A., Kamli M. R., Albukhari S. M., Althabaiti S. A., Al-Harbi S. A., Khan I., Malik M. A. (2023). Water.

[cit44] Kim K. J., Kreider P. B., Chang C. H., Park C. M., Ahn H. G. (2013). J. Nanopart. Res..

[cit45] Sun M., Wang M., Ge C., Huang J., Li Y., Yan P., Wang M., Lei S., Bai L., Qiao G. (2023). Sens. Actuators, B.

[cit46] Huang J., Zhou J., Liu Z., Li X., Geng Y., Tian X., Du Y., Qian Z. (2020). Sens. Actuators, B.

[cit47] Zhu X., Wang J., Yang D., Liu J., He L., Tang M., Feng W., Wu X. (2021). RSC Adv..

[cit48] Tsai Y. T., Chang S. J., Ji L. W., Hsiao Y. J., Tang I. T., Lu H. Y., Chu Y. L. (2018). ACS Omega.

[cit49] Adhikari S., Banerjee A., Eswar N. K. R., Sarkar D., Madras G. (2015). RSC Adv..

[cit50] Jin C., Li W., Chen Y., Li R., Huo J., He Q., Wang Y. (2020). Ind. Eng. Chem. Res..

[cit51] Rajamanickam D., Shanthi M. (2016). Arabian J. Chem..

[cit52] Onkani S. P., Diagboya P. N., Akpotu S. O., Mtunzi F. (2025). Ind. Eng. Chem. Res..

[cit53] Vijayakumar E., Raj M. G., Narendran M. G., Preetha R., Mohankumar R., Neppolian B., Bosco A. J. (2022). ACS Omega.

[cit54] Rauf M. A., Meetani M. A., Khaleel A., Ahmed A. (2010). Chem. Eng. J..

[cit55] Fauzia V., Yudiana A., Yulizar Y., Dwiputra M. A., Roza L., Soegihartono I. (2021). J. Phys. Chem. Solids.

[cit56] Hang D. R., Islam S. E., Chen C. H., Sharma K. H. (2016). Chem.–Eur. J..

[cit57] Saravanan R., Karthikeyan N., Gupta V. K., Thirumal E., Thangadurai P., Narayanan V. (2013). Mater. Sci. Eng. C..

[cit58] Shu J., Wang Z., Xia G., Zheng Y., Yang L., Zhang W. (2014). J. Chem. Eng..

[cit59] Komaraiah D., Radha E., Sivakumar J., Reddy M. V. R., Sayanna R. (2020). Opt. Mater..

[cit60] Iqbal S., Bahadur A., Javed M., Hakami O. (2021). Mater. Sci. Eng. B..

[cit61] Pathak T. K., Kroon R. E., Swart H. C. (2018). Vacuum.

[cit62] Zhao Z. J., Hwang S. H., Jeon S., Hwang B., Jung J. Y., Lee J., Park S. H., Jeong J. H. (2017). Sci. Rep..

