# Supplementary material for: Enhanced visible-light photocatalysis by Au and Ag decorated ZnO for the simultaneous degradation of tetracycline and methylene blue
Source: Nanoscale Adv. 2026 Jan 6;8(5):1686–711. doi: 10.1039/d5na00878f (PMC12880110; doi:10.1039/d5na00878f)
Supplement: NA-008-D5NA00878F-s001 [file NA-008-D5NA00878F-s001.pdf]

Supplementary Information (SI) for Nanoscale Advances.
This journal is © The Royal Society of Chemistry 2026

Supplementary Information

Enhanced Visible-light Photocatalysis by Au and Ag decorated ZnO for the Simultaneous Degradation of Tetracycline and Methylene Blue

Asha Kumawat,[a] Sunil Chhichholiya,[a] Mamta Devi Sharma,[b] Poonam Kumari,[a] Rajesh Kumar Meena,[c] Pragati Fageria*[a]

[a]Centre of Advanced Studies, Department of Chemistry, University of Rajasthan, Jaipur, 332004, India

[b]Department of Clean Energy and Fuel Cell, Dr. Bansi Dhar Institute, Gurugram, Haryana 122003, India

[c]Department of Chemistry, Kalindi College, University of Delhi, India

**E-mail: pragati.fageria@gmail.com*

SI1. Materials

All chemicals utilized were of an analytical reagent (AR) grade and were employed without further purification. Zinc nitrate hexahydrate [$Zn(NO_3)_2 \cdot 6H_2O$] and urea (NH_2CONH_2, extra pure) were procured from SD Fine-Chem Ltd. Hydrogen tetrachloroaurate (III) hydrate ($HAuCl_4 \cdot 3H_2O$) was obtained from Thermo Scientific. Silver nitrate ($AgNO_3$) and glucose ($C_6H_{12}O_6 \cdot H_2O$) were purchased from Alfa Aesar. Hydrazine monohydrate ($NH_2NH_2.H_2O$ 98%), methylene blue ($C_{16}H_{18}N_3SCl$), tetracycline hydrochloride ($C_{22}H_{24}N_2O_8.HCl$), methanol (CH_3OH), disodium ethylenediaminetetraacetic acid ($C_{10}H_{14}N_2Na_2O_8.2H_2O$), and ascorbic acid ($C_6H_8O_6$) were also obtained from commercial sources and used as received. Deionized (DI) water was used in all experiments. All reactions were carried out at room temperature unless otherwise specified.

SI2. Characterization tools

The optical properties of the synthesized nanocomposites were evaluated using a UV–Visible spectrophotometer (PerkinElmer, Lambda 1050+) over the wavelength range of 200–800 nm, with $BaSO_4$ employed as the reference standard. The crystalline structure of the samples was examined using X-ray diffraction (XRD) on a PANalytical's X'Pert Powder diffractometer equipped with Cu Kα radiation (λ = 1.5406 Å) operated at 40 mA and 45 kV. Diffraction patterns were recorded in the 2θ range of 20°–80° with a step size of 0.03° and a scan speed of 0.06° s^{-1}. Fourier-transform infrared (FTIR) spectra were obtained using a PerkinElmer Spectrum (version 10.4.00) in the range of 400–4000 cm^{-1}, employing KBr pellet methodology to identify functional groups. X-ray photoelectron spectroscopy (XPS) was performed on a Thermo Scientific spectrometer using a monochromatized Al Kα X-ray source, providing insights into the elemental composition, oxidation states, and chemical environments of the surface species.

The surface morphology and textural features of the photocatalysts were characterized by field emission scanning electron microscopy (FESEM, Apreo LoVac, FEI). Energy-dispersive X-ray spectroscopy (EDS) and elemental mapping were conducted using the same instrument at an accelerating voltage of 127 eV to confirm elemental composition and spatial distribution. High-resolution transmission electron microscopy (HRTEM) was performed using a Tecnai G2 20 S-TWIN (FEI) operating at 200 kV to investigate the internal structure, crystallinity, and lattice spacing of the synthesized nanocomposites. The specific surface area and pore volume were determined from nitrogen adsorption–desorption isotherms using

Brunauer–Emmett–Teller (BET) analysis on a Microtrac BEL (BELSORP mini II) instrument. Raman analysis was carried out using HORIBASCI Raman instrument (model no LabRAM HR EVO). Mass analysis was performed using a Shimadzu LCMS-8040 triple-quadrupole mass spectrometer equipped with ESI and APCI ionization sources. The solution pH was measured using a Labman pH meter (Model LMPH-10).

SI3: Salt addition method for determining the pH_{pzc}

The Point of Zero Charge (pH_{pzc}) for the pure ZnO, Au-ZnO, and Ag-ZnO catalysts was determined using the salt addition method with a 0.1 M NaCl background electrolyte solution. In brief, 30 mg of catalyst was added to 30 mL of 0.1 M NaCl in various sealed vessels. The initial pH (pH_i) of each suspension was adjusted to range from 2 to 12 using 0.1 M HCl and 0.1 M NaOH solutions. The suspensions were then agitated in a shaker for 24 hours at room temperature to ensure equilibrium. The final pH (pH_f) of each suspension was recorded. The pH_{pzc} was determined graphically by plotting the change in pH ($\Delta pH = pH_i - pH_f$) against the initial (pH_i); the pH_i at which ΔpH equals zero is defined as the pH_{pzc}.

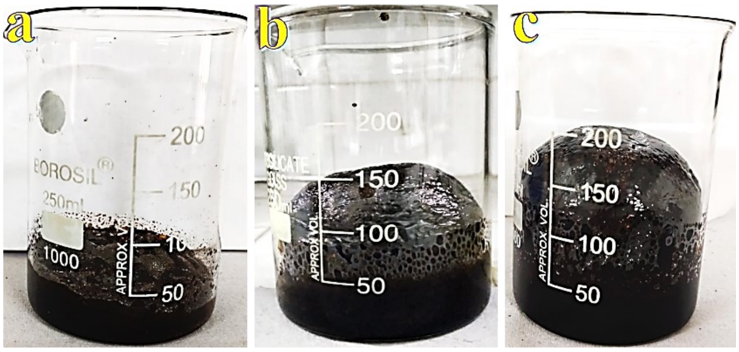

Fig. S1: Photographs of samples containing urea, glucose, and $Zn(NO_3)_2{\cdot}6H_2O$ after pre-calcination at (a) 120°C, (b) 140°C, and (c) 160°C for 6 h to form carbon foam

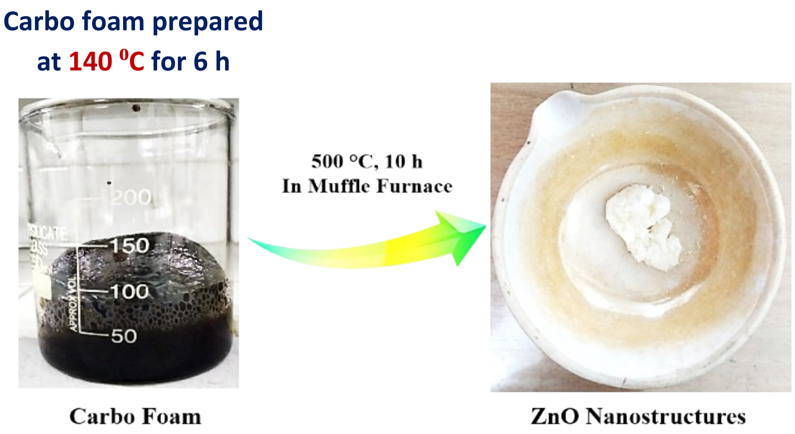

Fig. S2: ZnO nanostructure prepared through calcination at 500 °C for 10 hours

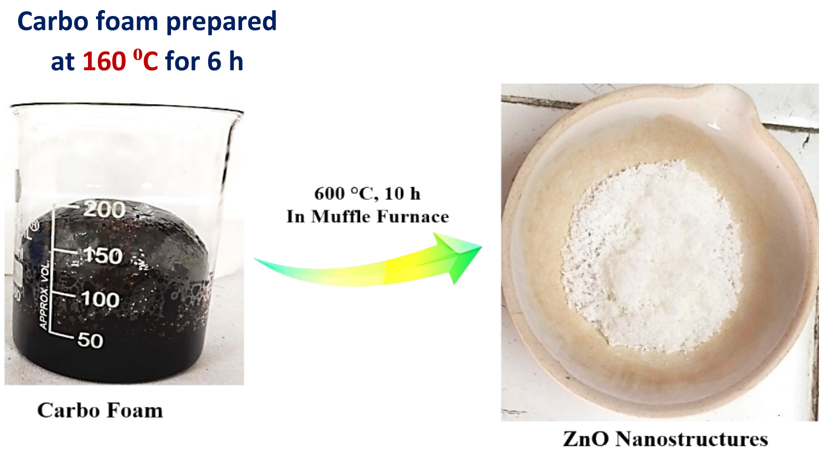

Fig. S3: ZnO nanostructures synthesized by calcination at 600 °C for 10 hours (our optimized method)

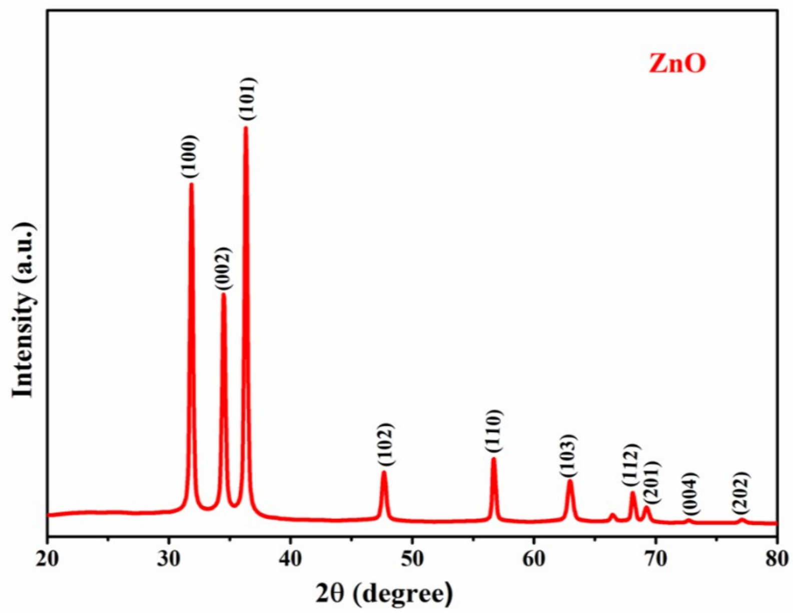

Fig. S4: PXRD of pure ZnO nanostructures (calcination at **600 °C** for 10h)

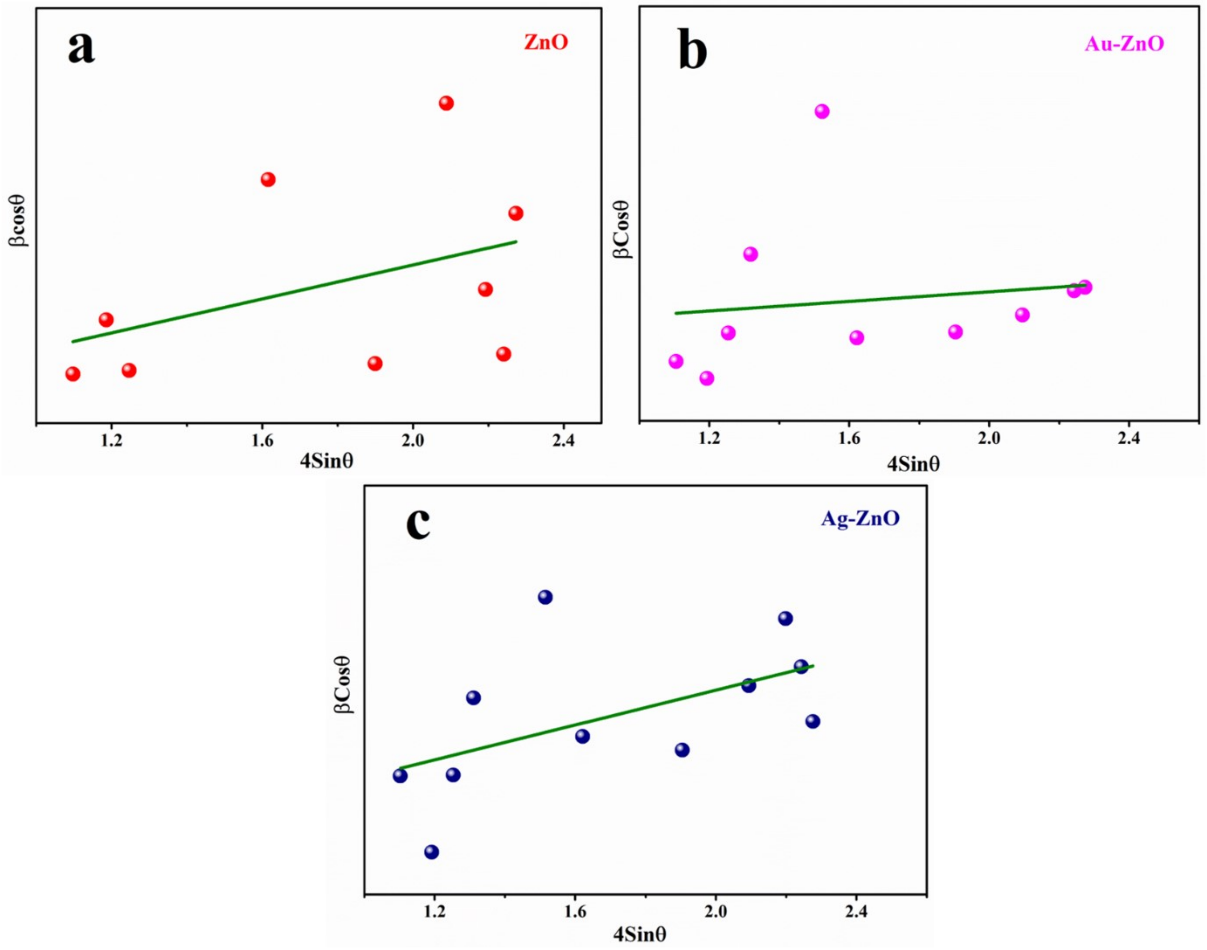

Fig. S5: Williamson-Hall plots of (a) pure ZnO NSs, (b) Au-ZnO NCs, and (c) Ag-ZnO NCs

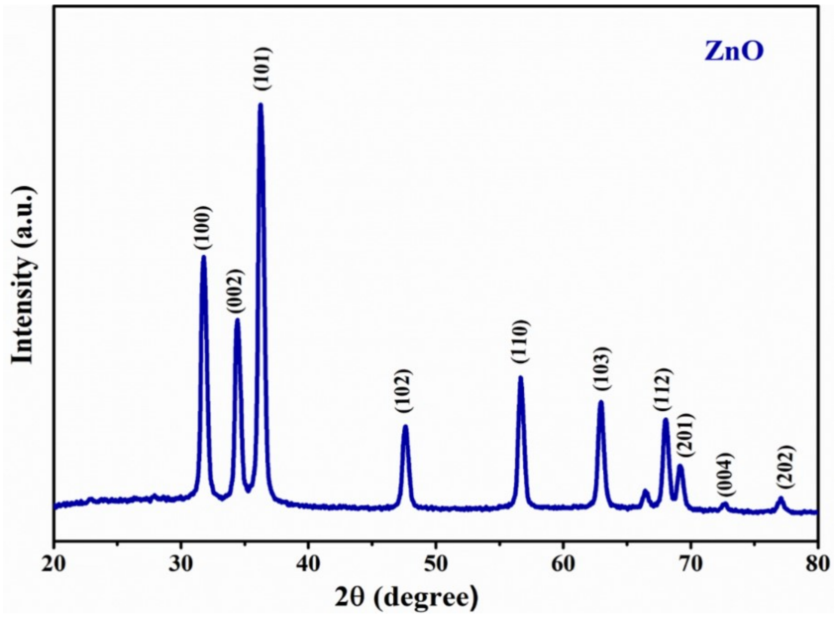

Fig. S6: PXRD of ZnO nanostructures prepared via calcination at **500 ^{o}C** for 10 h

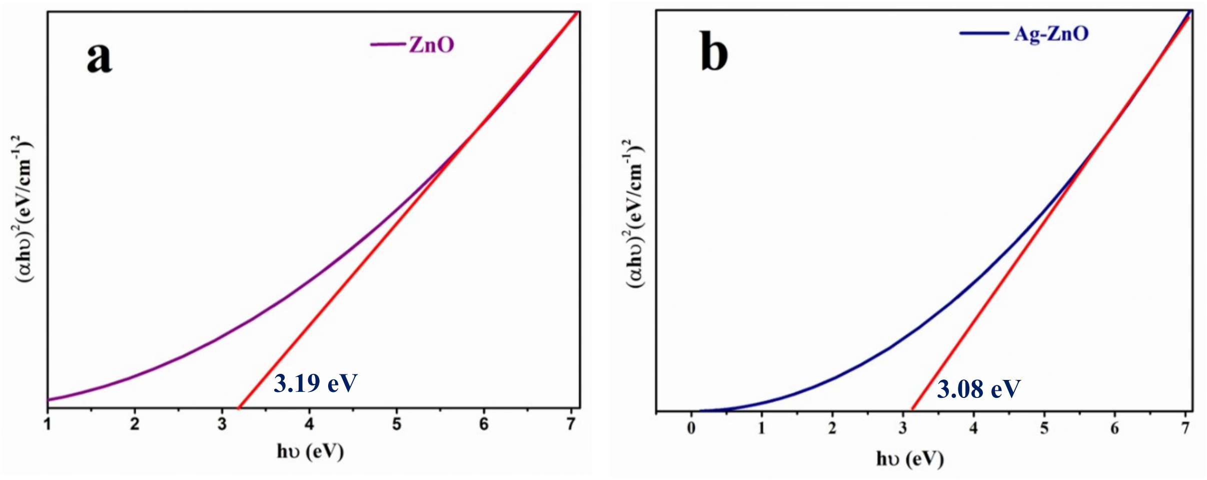

Fig. S7: Tauc plot for estimating the optical band gaps of (a) pure ZnO (b) Ag-ZnO by using UV–Vis diffuse reflectance spectra

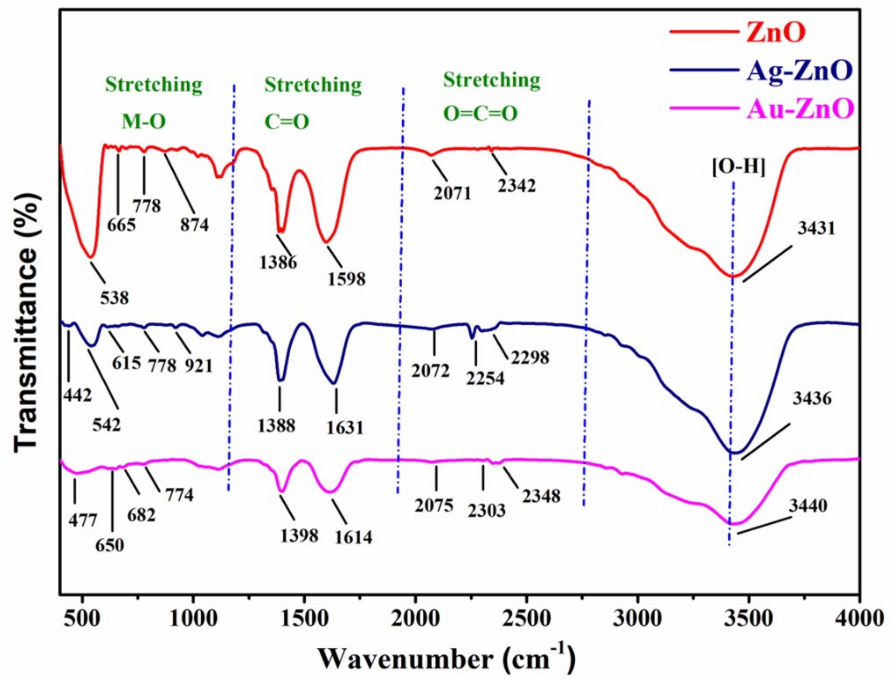

Fig. S8: FTIR spectra of ZnO, Ag-ZnO, and Au-ZnO

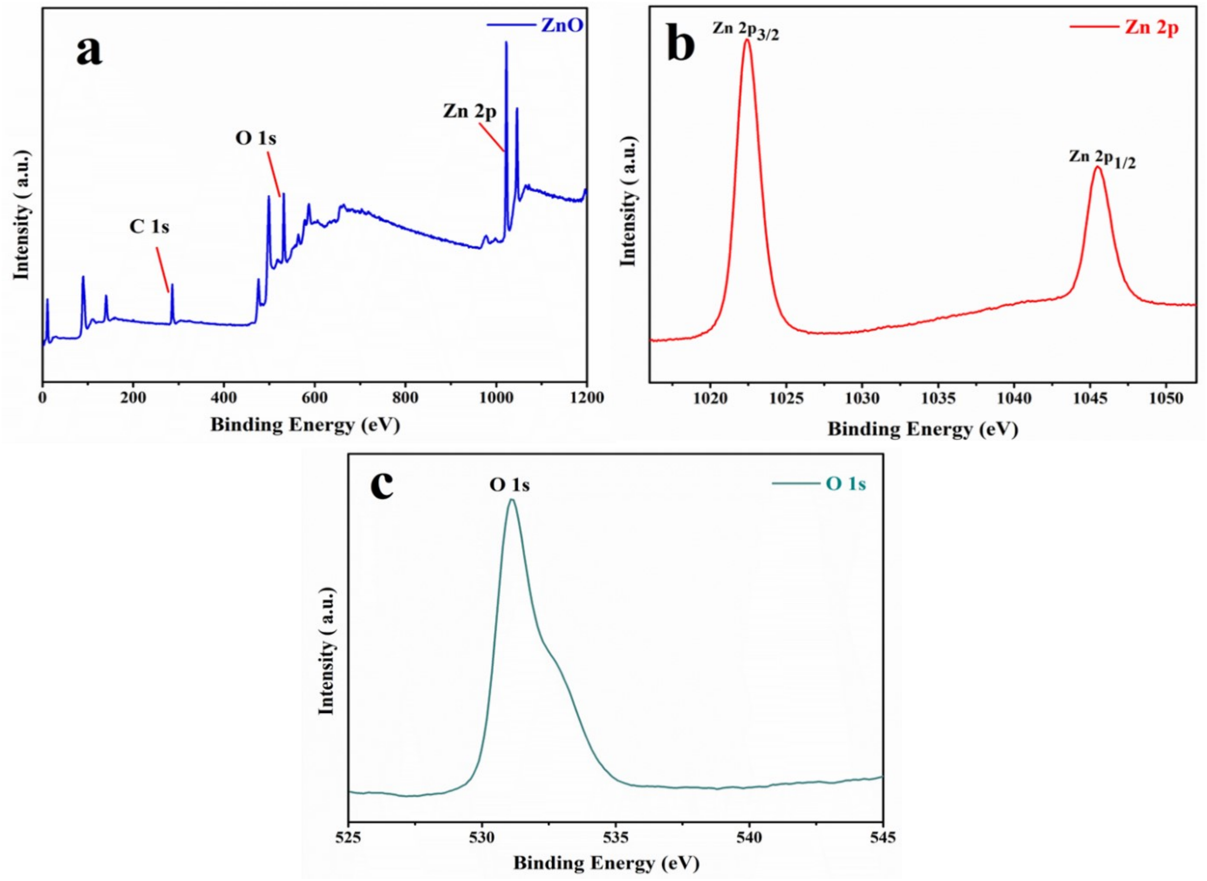

Fig. S9: XPS analysis of bare ZnO: (a) survey spectrum confirming Zn and O as primary elements; (b) high-resolution Zn 2p spectrum showing Zn^{2+} oxidation state; (c) O 1s spectrum indicating Zn–O bonds

Table S1: High-resolution XPS binding energies (eV) of Zn, O, C, Au, and Ag in ZnO, Au-ZnO, and Ag-ZnO NCs

Elements	Peak	ZnO (eV)	Au-ZnO (eV)	Ag-ZnO (eV)
**Zn**	$2p_{3/2}$	1021.34	1021.74	1021.30
	$2p_{1/2}$	1044.44	1044.89	1044.31
**O**	1s (Lattice O)	530.50	530.50	530.03
	1s (Defect O)	531.90	531.31	531.34
**C**	1s	284.80	284.82	284.56
**Au**	$4f_{7/2}$	-	83.54	-
	$4f_{5/2}$	-	87.25	-
**Ag**	$3d_{5/2}$	-	-	367.32
	$3d_{3/2}$	-	-	373.34

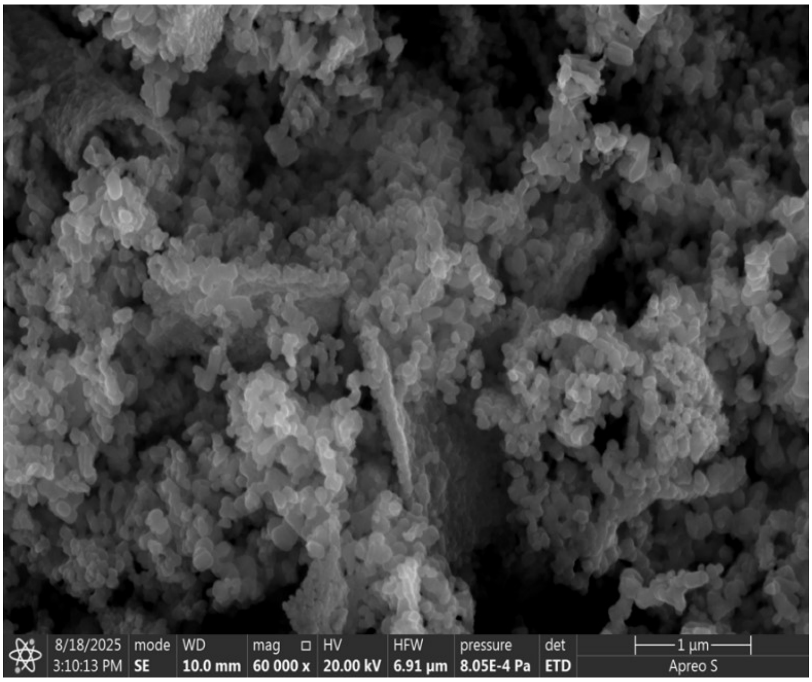

Fig. S10: FESEM images of ZnO nanostructures prepared via pre-calcination at 160 °C followed by calcination at 600 °C for 15 h (extended duration)

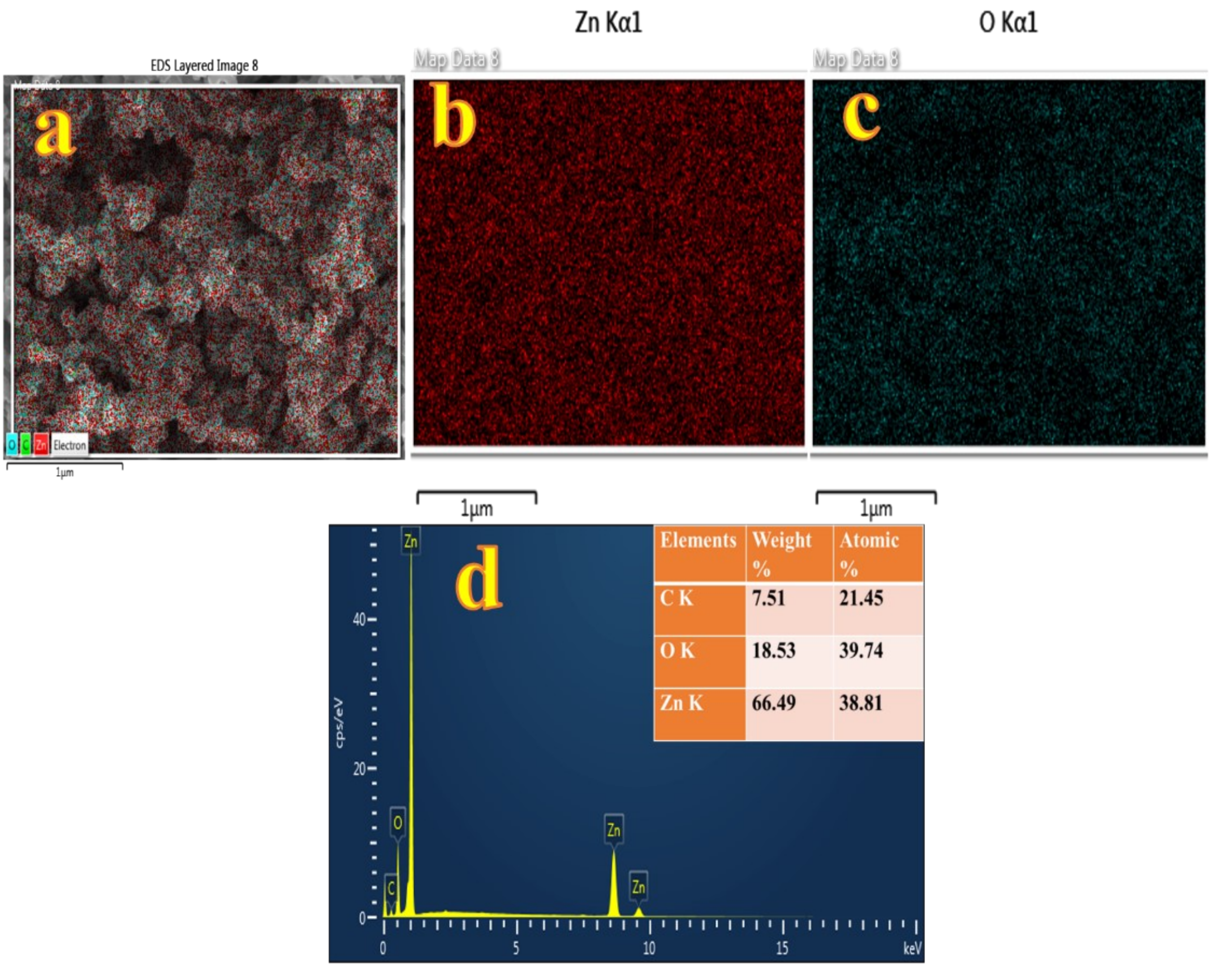

Fig. S11: EDS analysis of pure ZnO nanostructures: (a) Combined elemental overlay of Zn and O showing homogeneous distribution (b) Zn elemental map (c) O elemental map, and (d) EDS spectrum with inset table showing atomic percentages

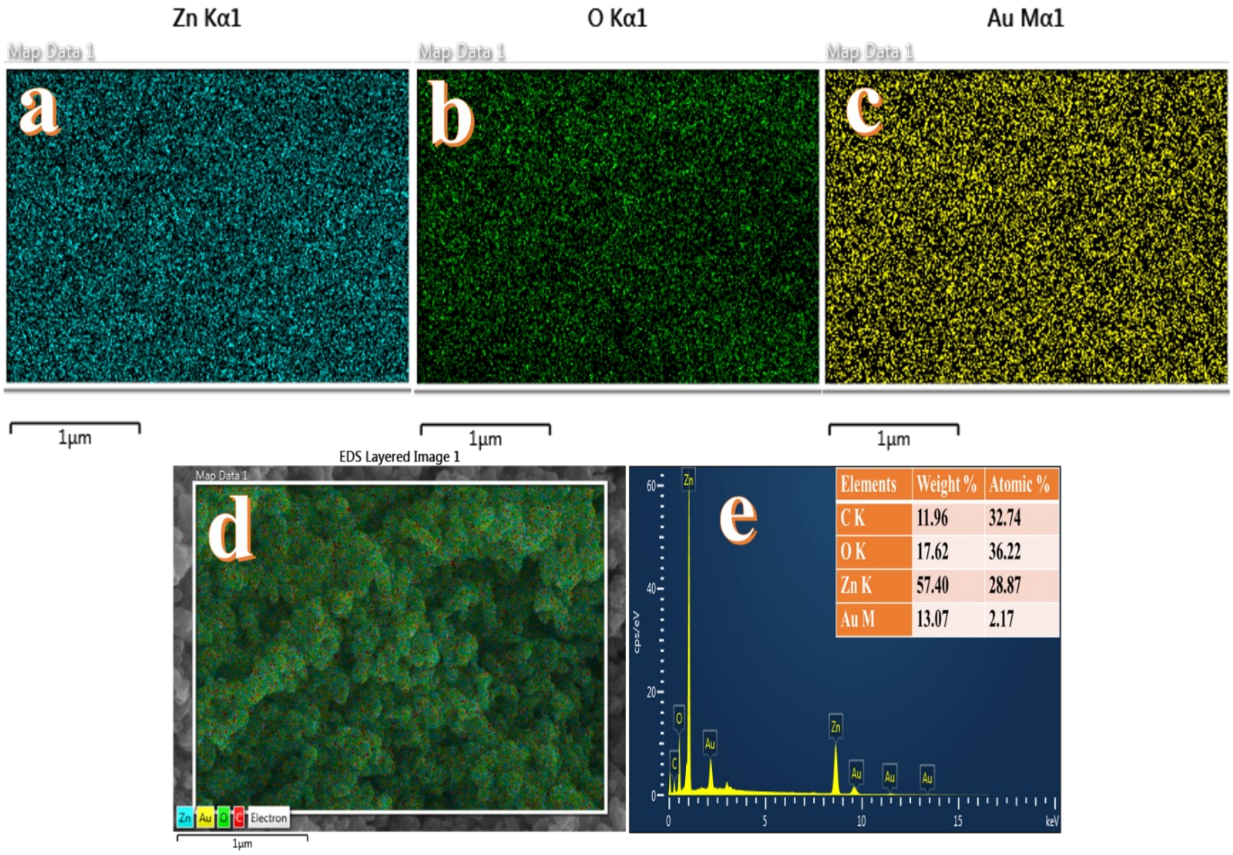

Fig. S12: EDS analysis of Au-ZnO NCs: (a) Zn elemental map, (b) O elemental map, (c) Au elemental map, (d) combined overlay showing co-distribution of Zn, O, and Au, and (e) EDS spectrum with elemental compositions

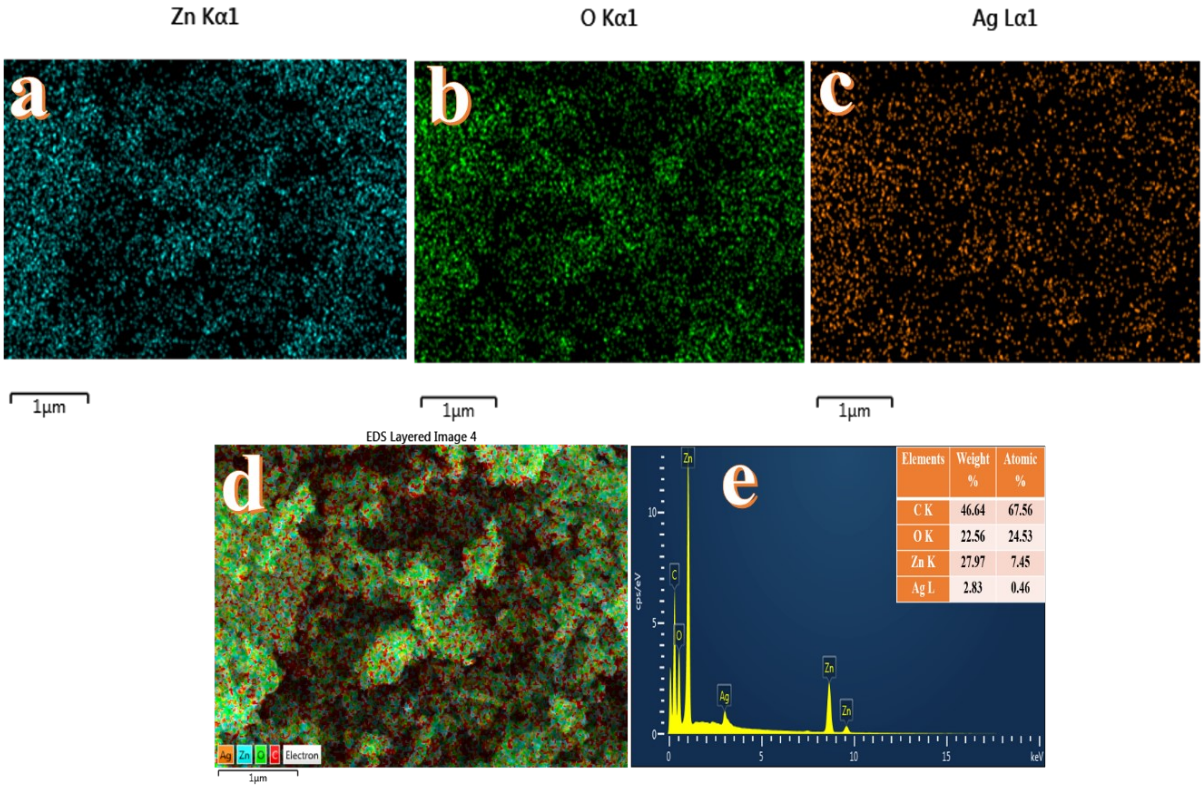

Fig. S13: EDS analysis of Ag-ZnO NCs: (a) Zn elemental map, (b) O elemental map (c) Ag elemental map (d) overlay of Zn, O, and Ag elemental distribution, and (e) EDS spectrum with corresponding atomic percentages (inset)

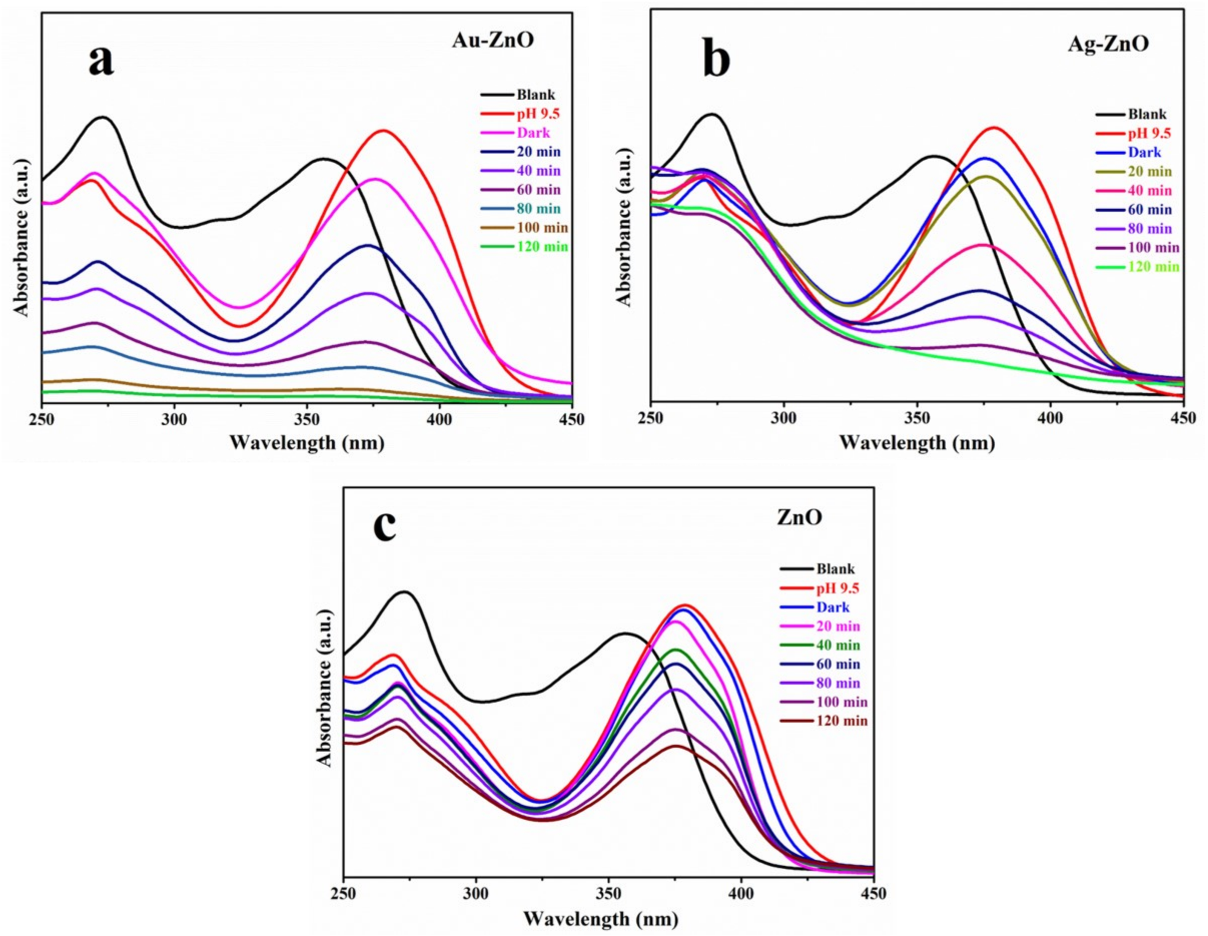

Fig. S14: UV-Vis spectra for degradation of TC using (a) Au-ZnO NCs and (b) Ag-ZnO NCs (c) ZnO under visible light at 9.5 pH

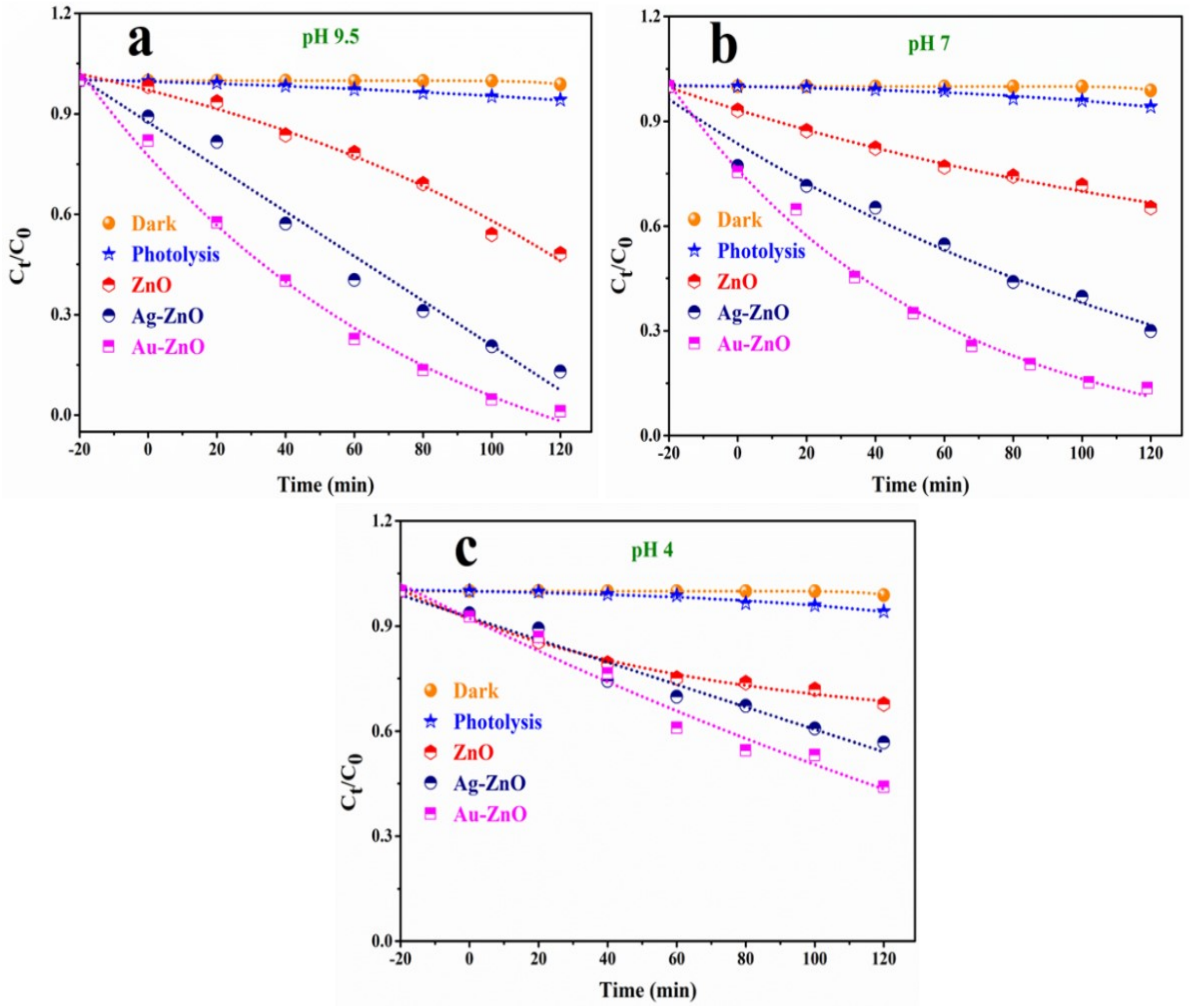

Fig. S15: Exponential degradation (C_t/C_0) of TC in the presence of ZnO, Au-ZnO and Ag-ZnO at different solution pH value (**a-c**)

Table S2. Reaction kinetic parameters (intercept, slope, and R^2) for TC degradation at varying pH values (9.5, 7, and 4) under dark, photolysis, and photocatalytic conditions using ZnO, Ag–ZnO, and Au–ZnO nanocomposites. All parameters were obtained from linear fits of *ln*(C_t/C_o) versus time plots.

pH - 9.5	Intercept		Slope		Statistics
	Value	Standard Error	Value	Standard Error	Adj. R-Square
ln(Ct/Co)	3.53613E-4	0.00158	-5.46329E-5	2.32471E-5	0.39252
Photolysis	-0.00256	0.00202	-4.46934E-4	2.97522E-5	0.96978
ZnO	-0.00764	0.04144	-0.0054	6.1106E-4	0.91669
Ag-ZnO	-0.08183	0.07931	-0.01481	0.00117	0.95791
Au-ZnO	-0.0781	0.26201	-0.0303	0.00386	0.8963

pH -7	Intercept		Slope		Statistics
	Value	Standard Error	Value	Standard Error	Adj. R-Square
Dark	8.44811E-4	0.00177	-5.19719E-5	2.60292E-5	0.29907
Photolysis	0.00154	0.00432	-4.34086E-4	6.36242E-5	0.86679
ZnO	-0.06406	0.01048	-0.00291	1.54592E-4	0.98053
Ag-ZnO	-0.167	0.03233	-0.0084	4.76701E-4	0.97791
Au-ZnO	-0.27427	0.03033	-0.01516	4.50172E-4	0.99299

pH - 4	Intercept		Slope		Statistics
	Value	Standard Error	Value	Standard Error	Adj. R-Square
Dark	8.44811E-4	0.00177	-5.19719E-5	2.60292E-5	0.29907
Photolysis	0.00154	0.00432	-4.34086E-4	6.36242E-5	0.86679
ZnO	-0.08591	0.01566	-0.0027	2.30851E-4	0.95082
Ag-ZnO	-0.06949	0.01889	-0.00428	2.78569E-4	0.97106
Au-ZnO	-0.07808	0.02685	-0.00604	3.95858E-4	0.97066

Table S3. Photodegradation efficiency and pseudo-first-order rate constants (*k*) for TC degradation using Au-ZnO, Ag-ZnO and ZnO at different pH values under visible light irradiation.

S. N.	Nanomaterials	Initial pH	% Degradation	Rate Constant (k in min^{-1})	Time (min)
1.	Au-ZnO	**9.5**	99	3.03×10^{-2}	**120 min**
2.	Ag-ZnO		87	1.48×10^{-2}	
3.	ZnO		51	5.4×10^{-3}	
4.	Au-ZnO	**7**	80	1.52×10^{-2}	
5.	Ag-ZnO		70.10	8.4×10^{-3}	
6.	ZnO		35.72	2.91×10^{-3}	
7.	Au-ZnO	**4**	55.93	6.04×10^{-3}	
8.	Ag-ZnO		43.20	4.28×10^{-3}	
9.	ZnO		32.30	2.70×10^{-3}	

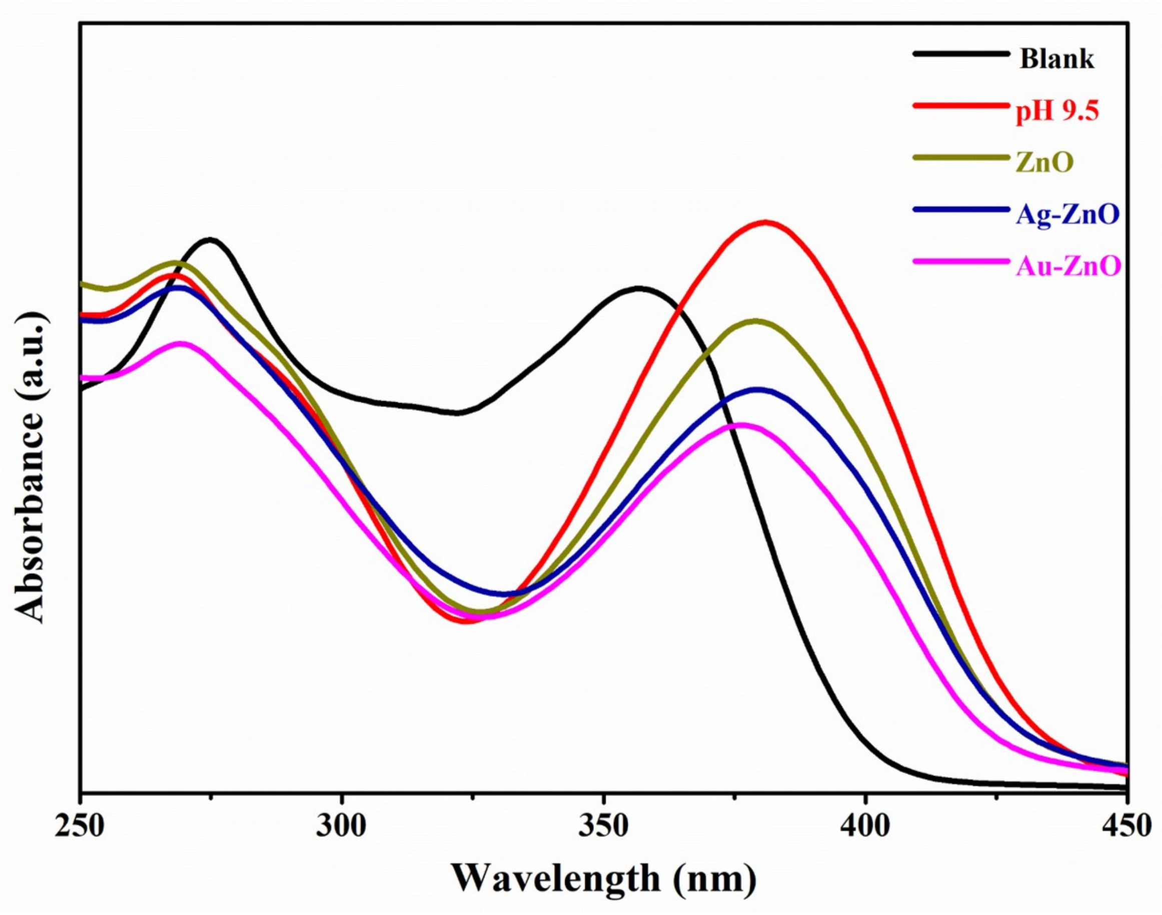

Fig. S16: UV-Vis spectra showing the photocatalytic degradation of TC (**3×10^{-3} M)** over 120 minutes at pH 9.5

Table S4. Pseudo-first order kinetics parameters for Methylene Blue dye (10^{-5} M) degradation using as-synthesized nanomaterials (Catalyst: 50 mg)

S. N.	Nanomaterials	Rate Constant (k min^{-1})	% Degradation	Time (min)
**1.**	Au-ZnO	5.175×10^{-2}	98 %	50
**2.**	Ag-ZnO	2.06×10^{-2}	97 %	120
**3.**	ZnO	0.251×10^{-2}	29.72%	120

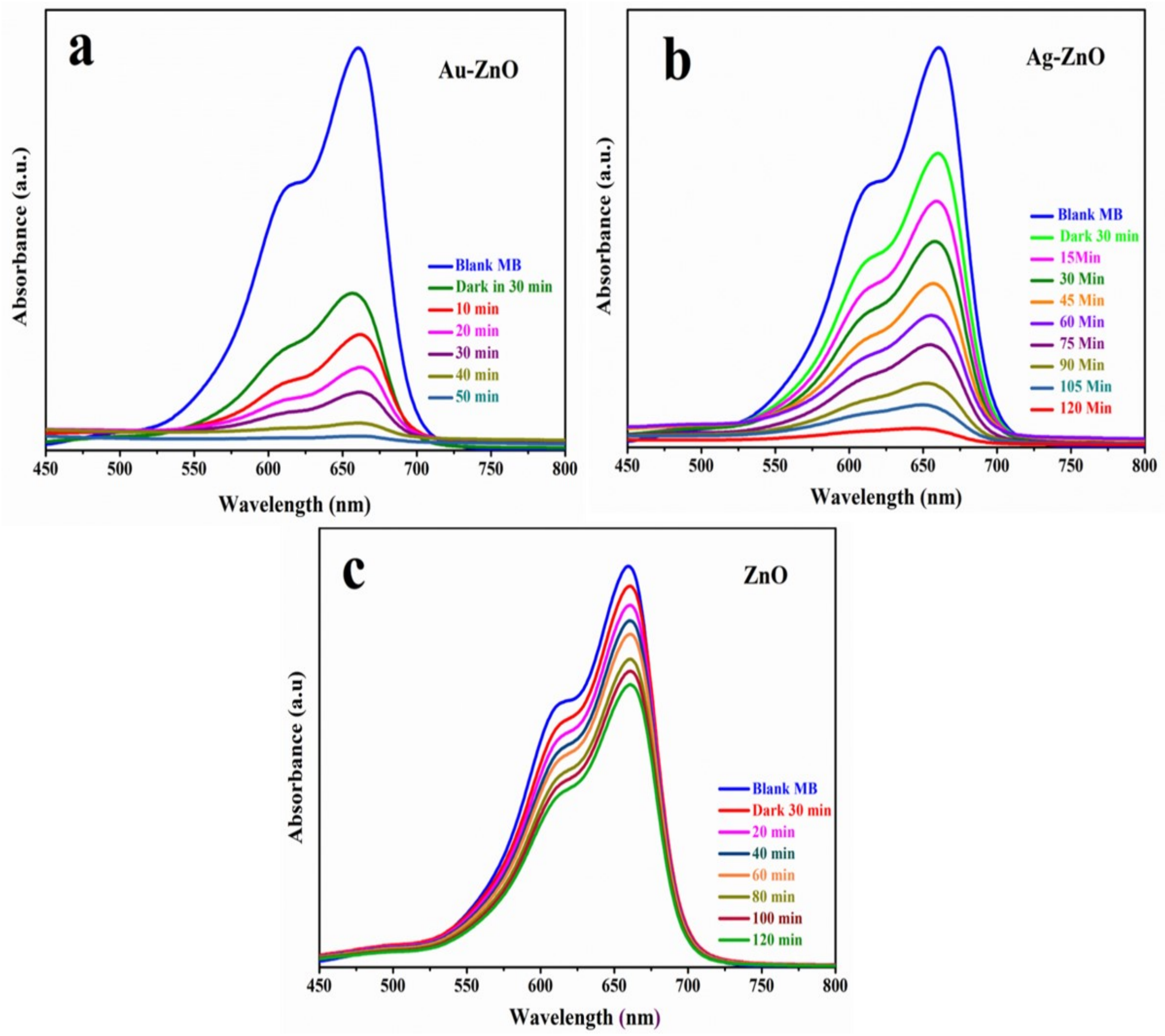

Fig. S17: UV–Vis spectra for the degradation of MB in the presence of (a) Au–ZnO NCs, (b) Ag–ZnO NCs and (c) ZnO nanostructures, under visible light

Table S5. Reaction kinetic parameters for MB degradation at pH 7 under dark, photolysis, and photocatalytic conditions using ZnO, Ag–ZnO, and Au–ZnO nanocomposites

pH - 7	Intercept		Slope		Statistics
	Value	Standard Error	Value	Standard Error	Adj. R-Square
Dark	0	0	0	0	--
Photolysis	-0.00165	5.78285E-4	-1.14757E-4	8.52635E-6	0.9626
ZnO	-0.04976	0.00399	-0.00251	5.88642E-5	0.99618
Ag-ZnO	-0.15194	0.14137	-0.02063	0.00208	0.91557
Au-ZnO	-0.81446	0.13899	-0.05175	0.00479	0.94352

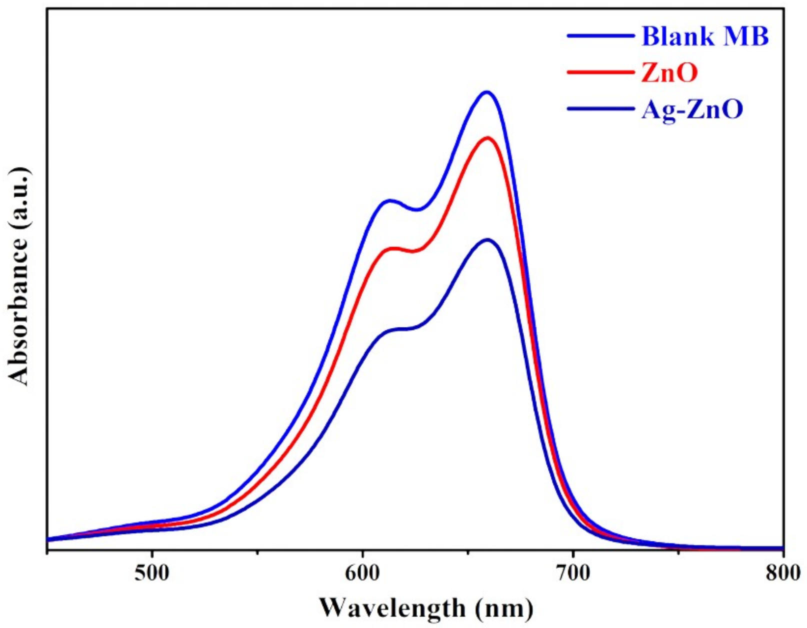

Fig. S18: UV–Vis spectra of MB degradation in the presence of ZnO and Ag–ZnO under visible light; [MB concentration: **2×10^{-5} M**, reaction time: 120 min.]

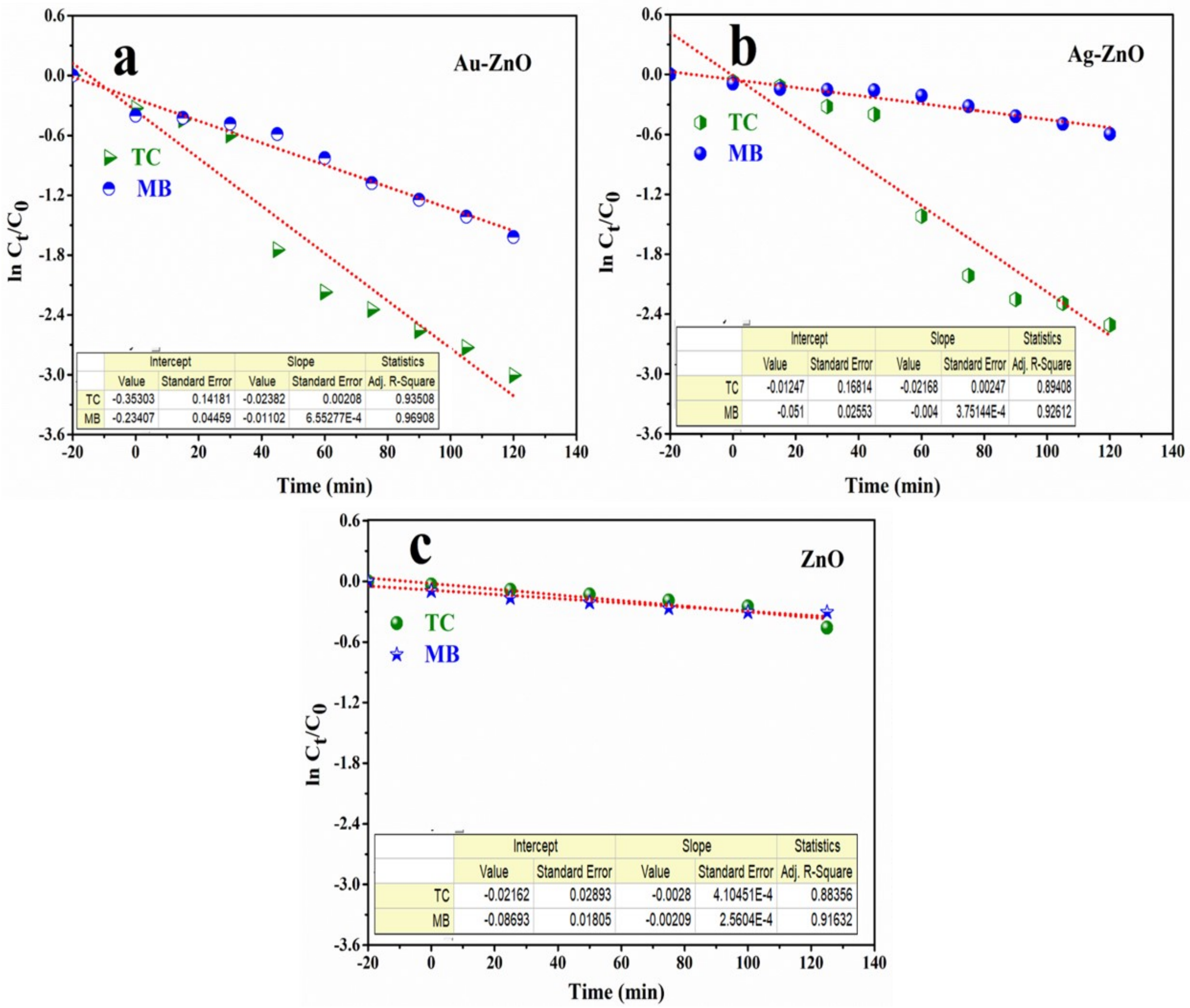

Fig. S19: (a–c) Co-degradation of tetracycline and methylene blue represented through *ln*(C_t/C_o) plots for Au–ZnO, Ag–ZnO, and ZnO photocatalysts, along with their corresponding reaction rate parameters

Table S6. Photocatalytic co-degradation percentages and pseudo-first-order rate constants (*k*) for TC and MB using the as-synthesized catalysts under visible-light irradiation at pH 7

Sample At pH =7	Amount of catalyst	Time taken in degradation	% of TC degraded	% of MB degraded	Rate constant TC	Rate constant MB
Au-ZnO	20 mg	120 min	95.04	80	2.38×10^{-2}	1.10×10^{-2}
Ag-ZnO			91.85	45	2.16×10^{-2}	0.40×10^{-2}
ZnO			36.71	26.4	0.28×10^{-2}	0.20×10^{-2}

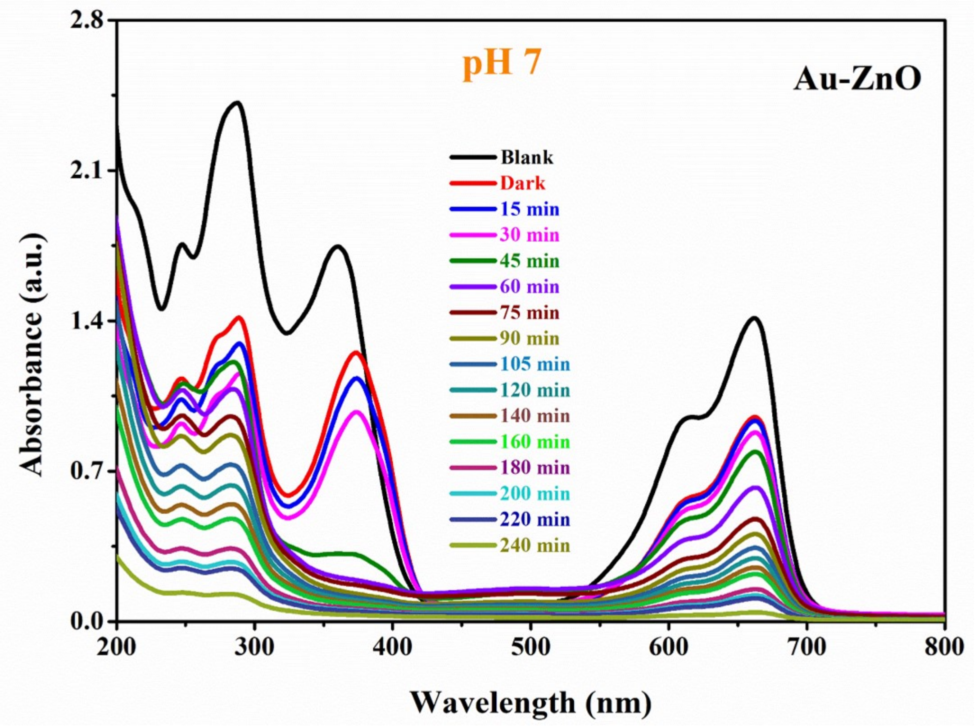

Fig. S20: Co-degradation of TC and MB using Au–ZnO (20 mg) after 240 minutes of visible-light irradiation (pH: 7)

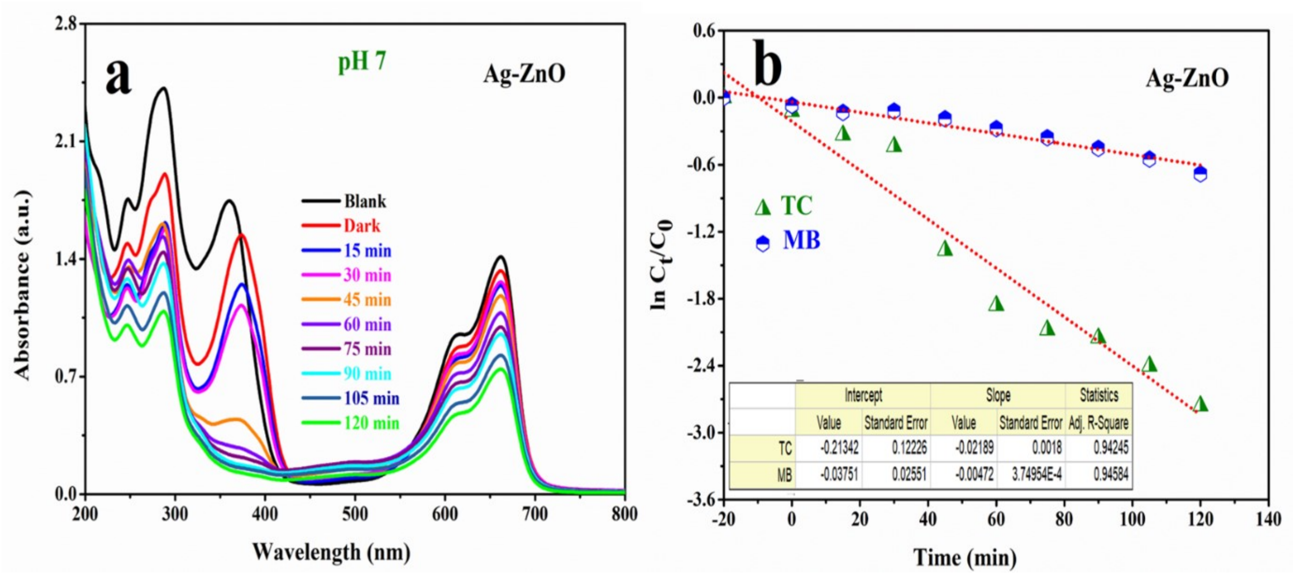

	Intercept		Slope		Statistics
	Value	Standard Error	Value	Standard Error	Adj. R-Square
TC	-0.21342	0.12226	-0.02189	0.0018	0.94245
MB	-0.03751	0.02551	-0.00472	3.74954E-4	0.94584

Fig. S21: Co-degradation of TC and MB using Ag–ZnO [50 mg]: (a) UV–vis absorption profiles and (b) *ln* C_t/C_0 plot with corresponding kinetic parameters

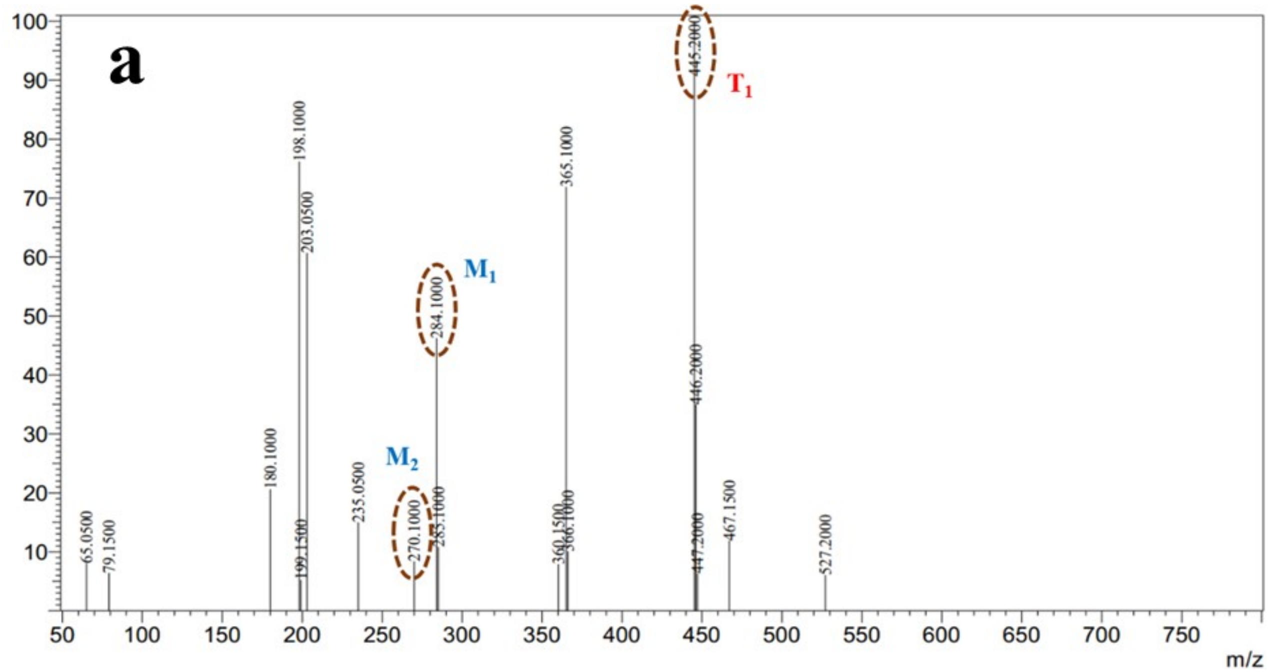
a
T1
M1
M2
65.0500
79.1500
180.1000
198.1000
199.1500
203.0500
235.0500
270.1000
284.1000
285.1000
360.1500
365.1000
366.1000
445.2000
446.2000
447.2000
467.1500
527.2000
m/z

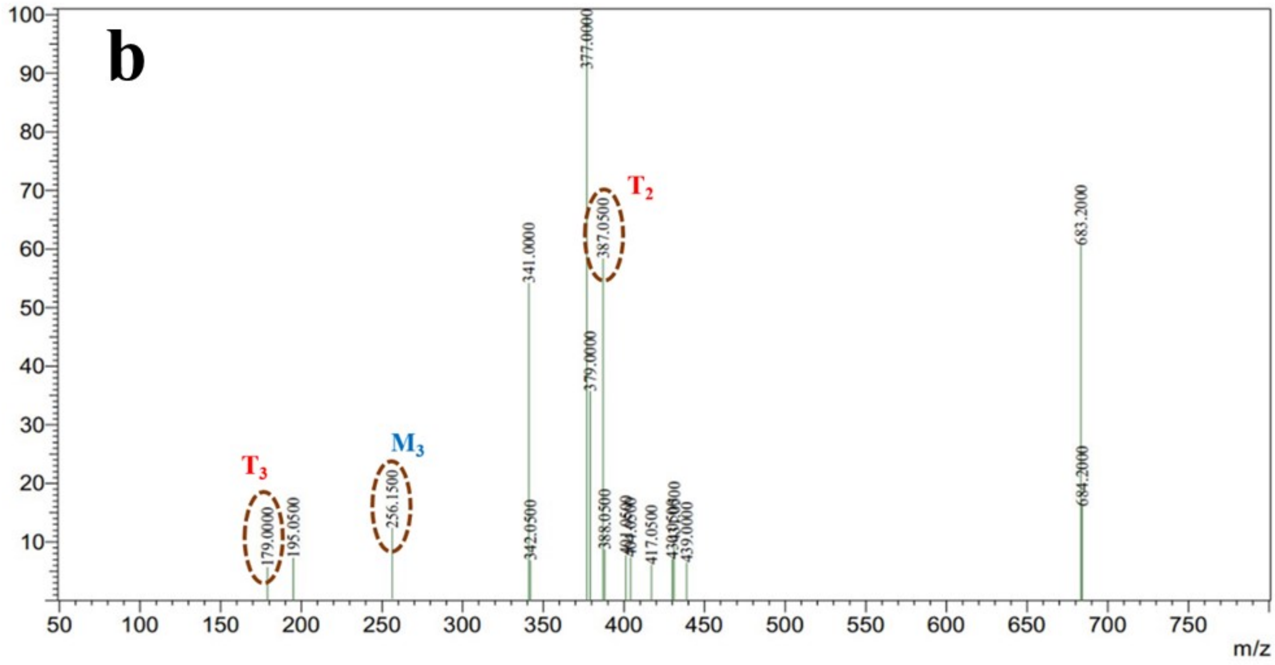
b
T2
T3
M3
179.0000
195.0500
256.1500
341.0000
342.0500
377.0000
379.0000
387.0500
388.0500
417.0500
439.0000
683.2000
684.2000
m/z

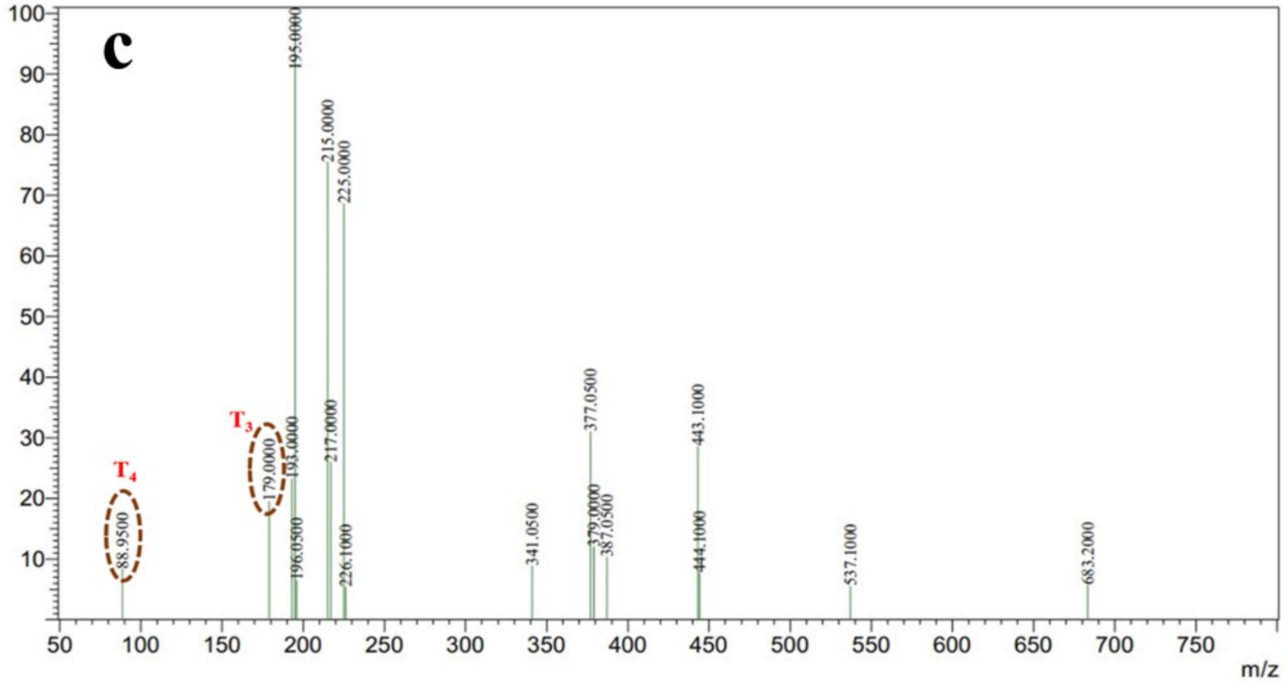

Fig. S22: Mass spectra of the reaction mixture during the co-degradation of tetracycline and methylene blue: (a) initial mixture [0 min], (b) after 60 min of photocatalytic treatment, and (c) after 120 min. The detected m/z fragments corresponding to TC (T_1, T_2, T_3) and MB (M_1, M_2, M_3) intermediates confirm the stepwise breakdown of both pollutants under visible-light irradiation

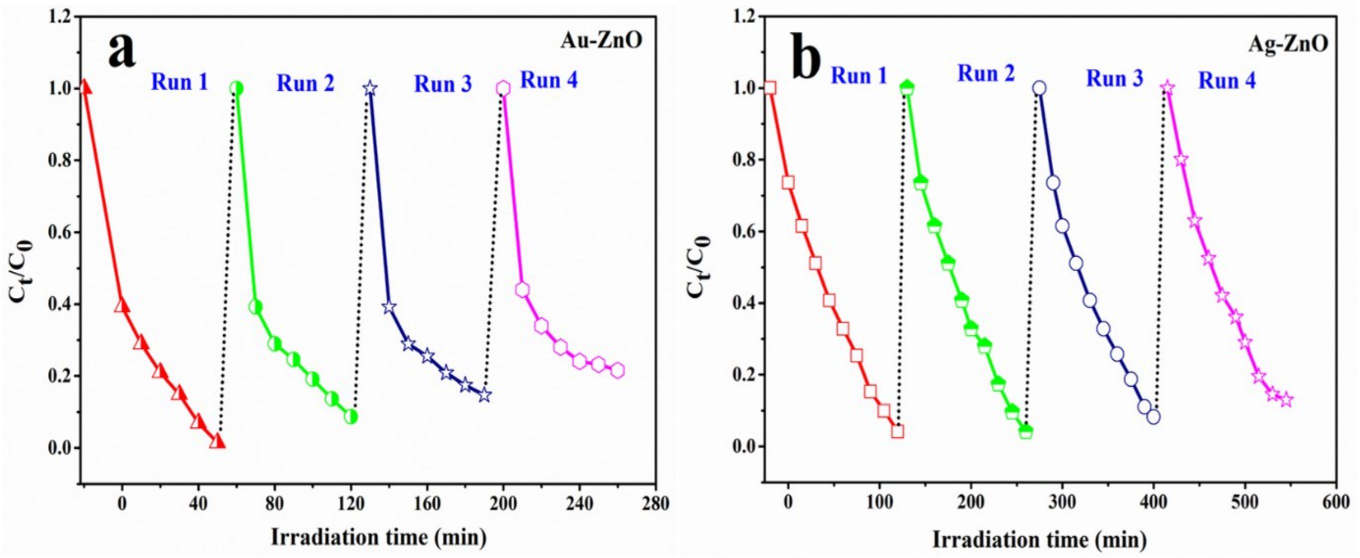

Fig. S23: Reusability of photocatalysts: (a) Au–ZnO and (b) Ag–ZnO for the degradation of methylene blue under visible light over four consecutive cycles

Table S7. Effect of different scavengers on the photocatalytic degradation of MB and TC using Ag-ZnO as a catalyst

**Scavengers used**	**To scavenge**	**% Degradation MB (10^{-5} M)**	**% Degradation of TC ($2x10^{-3}$M)**
**No Scavenger**		97	87
**$AgNO_3$**	e^-	58.44	56
**EDTA**	h^+	38.17	41
**Methanol**	$^{\bullet}OH$	37	39
Ascorbic Acid	$^{\bullet}O_2^-$	*19.66*	*22*

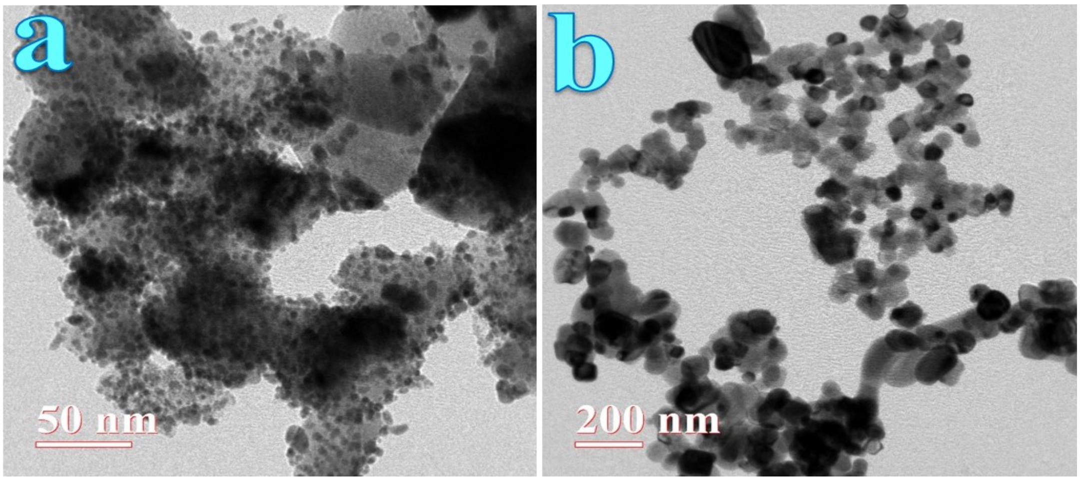

Fig. S24: HRTEM images of post-catalysis (a) Au–ZnO and (b) Ag–ZnO nanocomposites after four reuse cycles for TC degradation

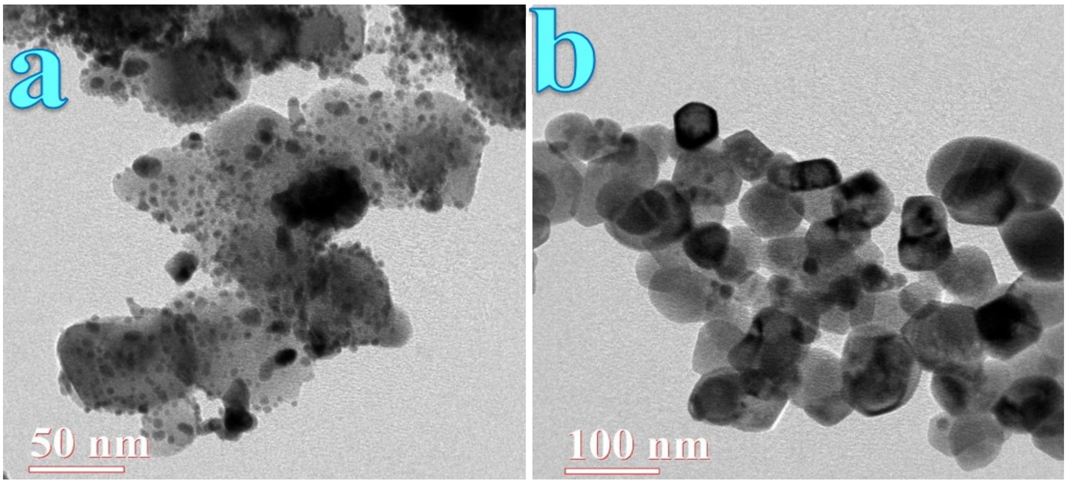

Fig. S25: Post-catalysis HRTEM images of (a) Au–ZnO and (b) Ag–ZnO after four reuse cycles for MB degradation

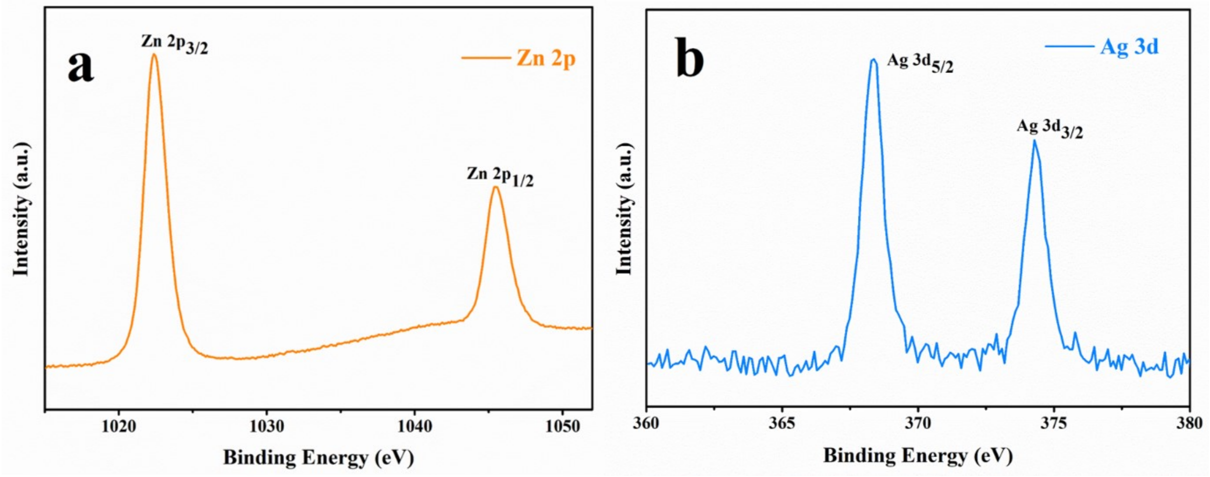

Fig. S26: XPS spectra of Ag–ZnO NCs after four successive photocatalytic cycles for MB degradation, showing (a) Zn 2p and (b) Ag 3d core-level regions

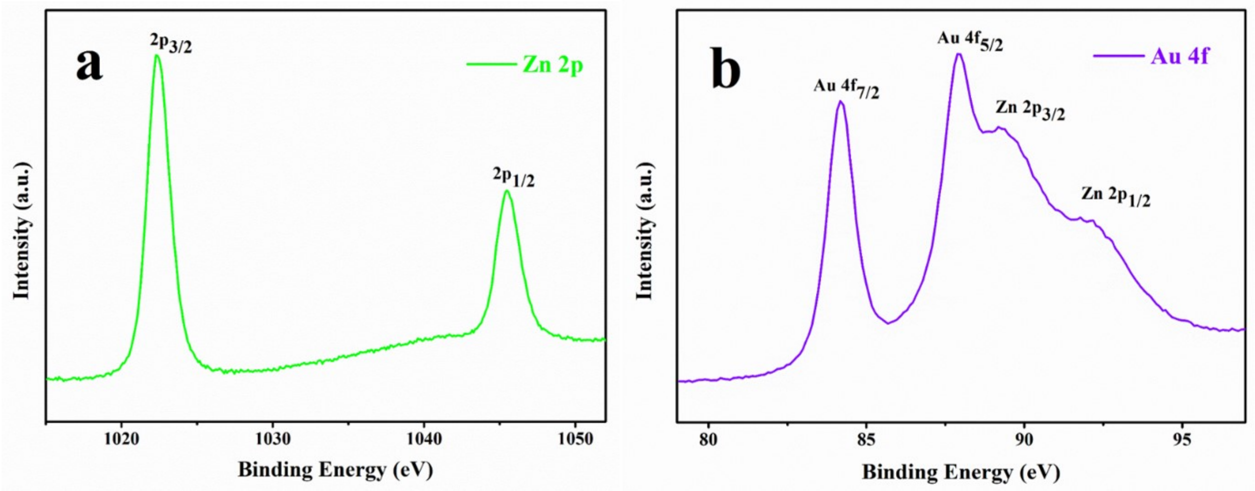

Fig. S27: XPS spectra of Au–ZnO NCs after four successive photocatalytic cycles for MB degradation, showing (a) Zn 2p and (b) Au 4f core-level regions

Table S8. Comparative binding energies (BEs) of Zn, O, Ag, and Au for fresh (before catalysis) and reused (after four photocatalytic cycles) Ag–ZnO and Au–ZnO nanocomposites

**Elements**	**Peak**	**Au-ZnO (eV) Before catalysis**	**Au-ZnO (eV) After-Catalysis**	**Ag-ZnO (eV) Before catalysis**	**Ag-ZnO (eV) After-Catalysis**
**Zn**	$2p_{3/2}$	1021.74	1022.44	1021.30	1022.43
	$2p_{1/2}$	1044.89	1045.47	1044.31	1045.43
**O**	1s (Lattice O)	530.50	531.17	530.03	531.15
	1s (Defect O)	531.31	532.29	531.34	532.67
**Au**	$4f_{7/2}$	83.54	84.17	-	-
	$4f_{5/2}$	87.25	87.89	-	-
**Ag**	$3d_{5/2}$	-	-	367.32	368.34
	$3d_{3/2}$	-	-	373.34	374.12
